# Genome-reduced Aridivitia evolved predation to thrive in Earth’s oligotrophic depths

**DOI:** 10.1038/s41467-026-78342-x

**Published:** 2026-10-14

**Authors:** Xiaonan Wan, Wenlu Feng, Yang Zhang, Tom A. Williams, Gergely J. Szöllősi, Hongyu Dang, Xuejun Yan, James M. Tiedje, Jizhong Zhou, Xun Qian

**Affiliations:** 1 College of Natural Resources and Environment, Northwest A&F University, Yangling, China; 2 Key Laboratory of Plant Nutrition and Agro-environment in Northwest China, Ministry of Agriculture and Rural Affairs, Northwest A&F University, Yangling, China; 3 State Key Laboratory of Soil and Water Conservation and Desertification Control, Northwest A&F University, Yangling, China; 4 Department of Life Sciences, University of Bath, Bath, UK; 5 Model-Based Evolutionary Genomics Unit, Okinawa Institute of Science and Technology Graduate University, Okinawa, Japan; 6 Institute of Evolution, HUN-REN Center for Ecological Research, Budapest, Hungary; 7 Center for Microbial Ecology, Michigan State University, East Lansing, MI USA; 8 Institute for Environmental Genomics, University of Oklahoma, Norman, OK USA

**Keywords:** Bacterial genomics, Microbial ecology, Soil microbiology, Phylogenetics

## Abstract

Deep soils represent one of Earth’s largest yet most enigmatic microbial habitats, where survival strategies under extreme oligotrophy remain poorly understood. Here, we report the Aridivitia group, a globally distributed bacterial lineage enriched in oligotrophic soils and thriving down to 22 m depth across sites in the United States and China. Despite its genome reduction (average size ~2.9 Mb), Aridivitia exhibits high metabolic flexibility in utilizing diverse carbon and energy sources. Cellular, genomic, and metatranscriptomic evidence, including small cell and genome sizes, host-associated metabolism, and the active expression of attachment- and attack-associated surface structures such as Tad pili and pore-forming toxins, indicate a predatory lifestyle in Aridivitia. Ancestral genome reconstruction reveals that Aridivitia originated as free-living mixotrophs, but subsequently lost the ability to fix carbon as they evolved a predatory lifestyle. Collectively, Aridivitia exemplifies a unique evolutionary strategy that reconciles genome reduction with functional versatility, expanding our understanding of microbial adaptation and evolution at the energetic limits of life.

## Abstract (web summary)

This study reveals that the uncultivated bacterial group Aridivitia thrives in oligotrophic deep soils, with reduced genomes, metabolic flexibility, and an evolutionary transition from free-living mixotrophy to predation.

## Introduction

The Earth’s Critical Zone (CZ) is the near-surface layer of the Earth extending from the top of the vegetation canopy to subsurface groundwater, linking the atmosphere, pedosphere, biosphere, hydrosphere, and lithosphere, and supporting the functioning of terrestrial ecosystems^1^. However, for decades, research on soil microorganisms within this system has predominantly focused on biologically active, resource-rich surface soils (0–0.3 m), largely overlooking the vast vertical reaches beneath our feet and leaving deep soils (>0.3 m) as an enigmatic “black box” within the CZ. Compared to surface soils, deep soils generally face severe nutrient and energy limitations, featuring low nutrient availability^2,3^ and chemically stable organic carbon that decomposes and turns over extremely slowly, thereby greatly restricting microbial access^4^. Consequently, deep soils constitute a vast yet long-neglected extreme oligotrophic biosphere. This chronic resource limitation constrains microbial survival, resulting in a significantly lower microbial biomass density per unit volume than in surface soils^5,6^. Nevertheless, owing to their immense spatial volume, the total microbial population in deep soils remains comparable to that in surface soils^7,8^. In this resource-depleted “subsurface desert”, how microorganisms persist, remain active, and maintain ecological function remains an unresolved scientific puzzle, with answers critical for understanding the limits of life and the potential role of deep life in global biogeochemical cycles.

The Aridivitia group (formerly UBA4738; 16S rRNA sequence name 0319-7L14) within Actinomycetota represents a lineage adapted to these oligotrophic environments. This lineage was first discovered as a dominant population in agricultural soil low in organic carbon^9^. However, due to the long-standing absence of cultured representatives, its functional traits remained largely unknown until the Uncultured Bacteria and Archaea (UBA) project released the first metagenome-assembled genomes (MAGs) in 2017^10^. Subsequently, Aridivitia MAGs have been reconstructed from various extreme and oligotrophic environments, including Antarctic desert soils^11^, volcanic hot springs^12^, sponge symbionts^13^, Tibetan Plateau permafrost^14^, and arid desert soils^15^, suggesting a competitive advantage in nutrient-poor ecosystems. Scattered genomic reports indicate the potential for carbon fixation via the Calvin–Benson–Bassham (CBB) cycle^12^, ammonia oxidation^16^, and atmospheric trace gas (H₂ and CO) oxidation^11^. Nevertheless, a systematic analysis of the lineage’s environmental distribution, intra-lineage diversity, metabolic strategies, and evolutionary history is lacking, severely constraining our understanding of its ecological role in oligotrophic ecosystems.

Here, we show that Aridivitia exhibits predominance in oligotrophic deep soils, where it is consistently enriched and highly transcriptionally active down to 22 m depth across 10 profiles from the United States and China. We recovered high-quality MAGs from these profiles and integrated them with public genomes to construct a comprehensive Aridivitia genome resource. Through genome-resolved analyzes and ancestral genome reconstruction, combined with metatranscriptomic data and size-fractionated filtration experiments, we investigated whether Aridivitia possesses specialized strategies for nutrient and energy acquisition and how these strategies evolved through the gain and loss of functional genes during adaptation to extreme oligotrophic conditions. Together, these findings identify a previously underappreciated strategy for microbial success under extreme energy limitation.

## Results

### Global soil distribution of Aridivitia

**Fig. 1:**
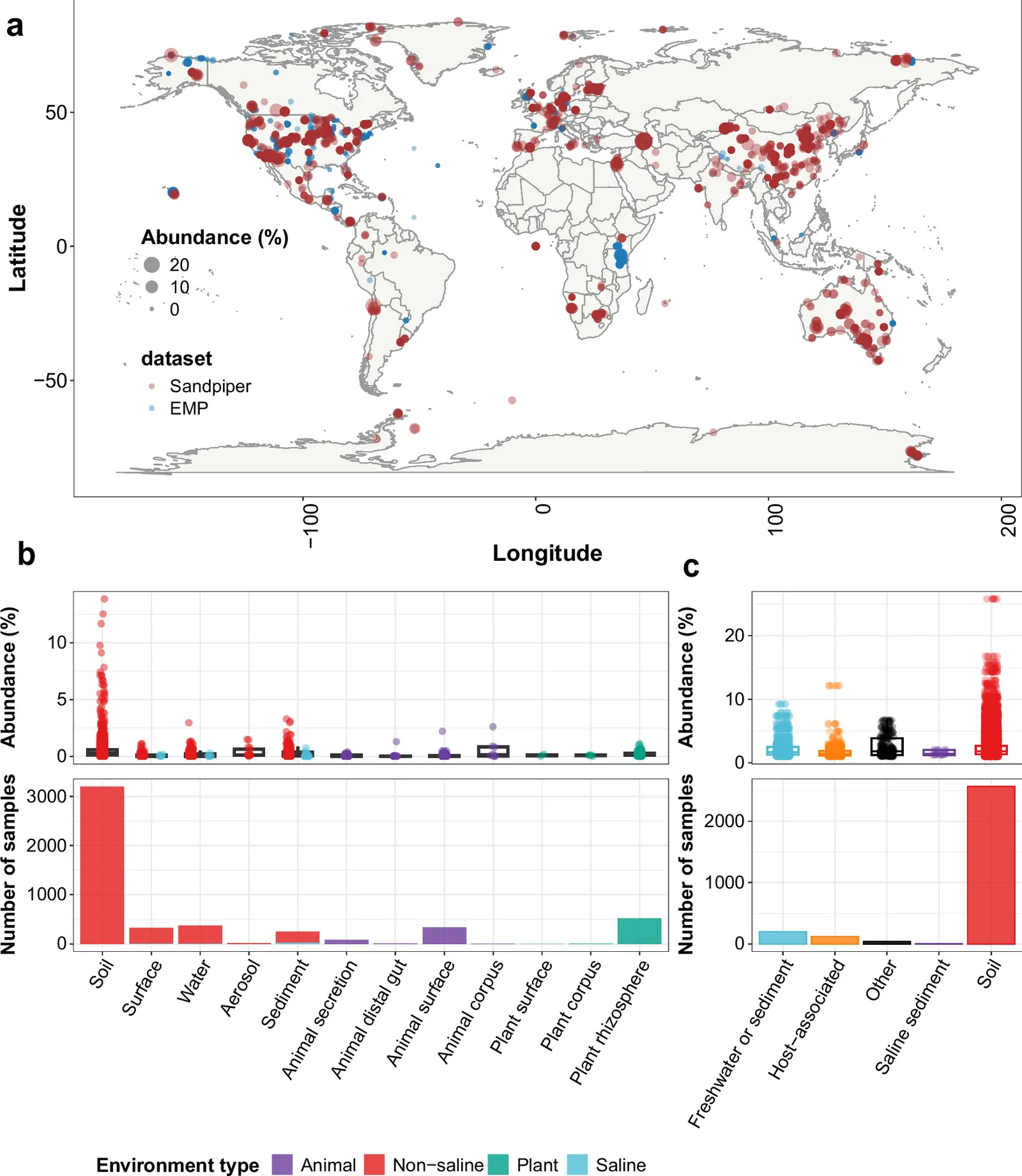
Geographic and environmental distribution of Aridivitia. **a** Global distribution of Aridivitia based on the Earth Microbiome Project (EMP) amplicon and Sandpiper metagenomic datasets. **a** Created using R, ggplot2, and map_data(“world”). Habitat distribution of Aridivitia across different biomes according to (**b**) EMP standards (*n* = 5161 samples) and (**c**) the Sandpiper metagenomic survey (*n* = 2931 samples). Each dot represents an individual sample. In both panels, the upper plots represent relative abundance, calculated as the proportion of reads assigned to Aridivitia within each sample, while the lower plots indicate the number of positive samples. Box plots show the median (center line), the 25th and 75th percentiles (box limits), and whiskers extending to the most extreme values within 1.5× the interquartile range. Source data are provided as a Source data file.

Environmental surveys using amplicon data from the Earth Microbiome Project (EMP)^17^ and global metagenomic datasets from Sandpiper^18^ revealed a global-scale distribution for Aridivitia (Fig. 1a, and Supplementary Note S1). The group was detected in 22% of EMP samples, predominantly in non-saline environments (soil, freshwater, sediments) (Fig. 1b). Among these Aridivitia-positive samples, 62% were from soils, which together contributed 83% of the total Aridivitia reads across the EMP dataset (Fig. 1b). These soil samples spanned diverse biomes, including shrublands, forests, tundra, grasslands, and deserts (Supplementary Fig. 1). This soil predominance was further supported by Sandpiper (Fig. 1c), and 94% of publicly available Aridivitia MAGs were derived from soil environments (Supplementary Fig. 2, and Supplementary Data 1), suggesting that soils are the primary habitat of Aridivitia.

### Aridivitia exhibits an oligotrophic preference and **in situ** activity in deep soils

**Fig. 2:**
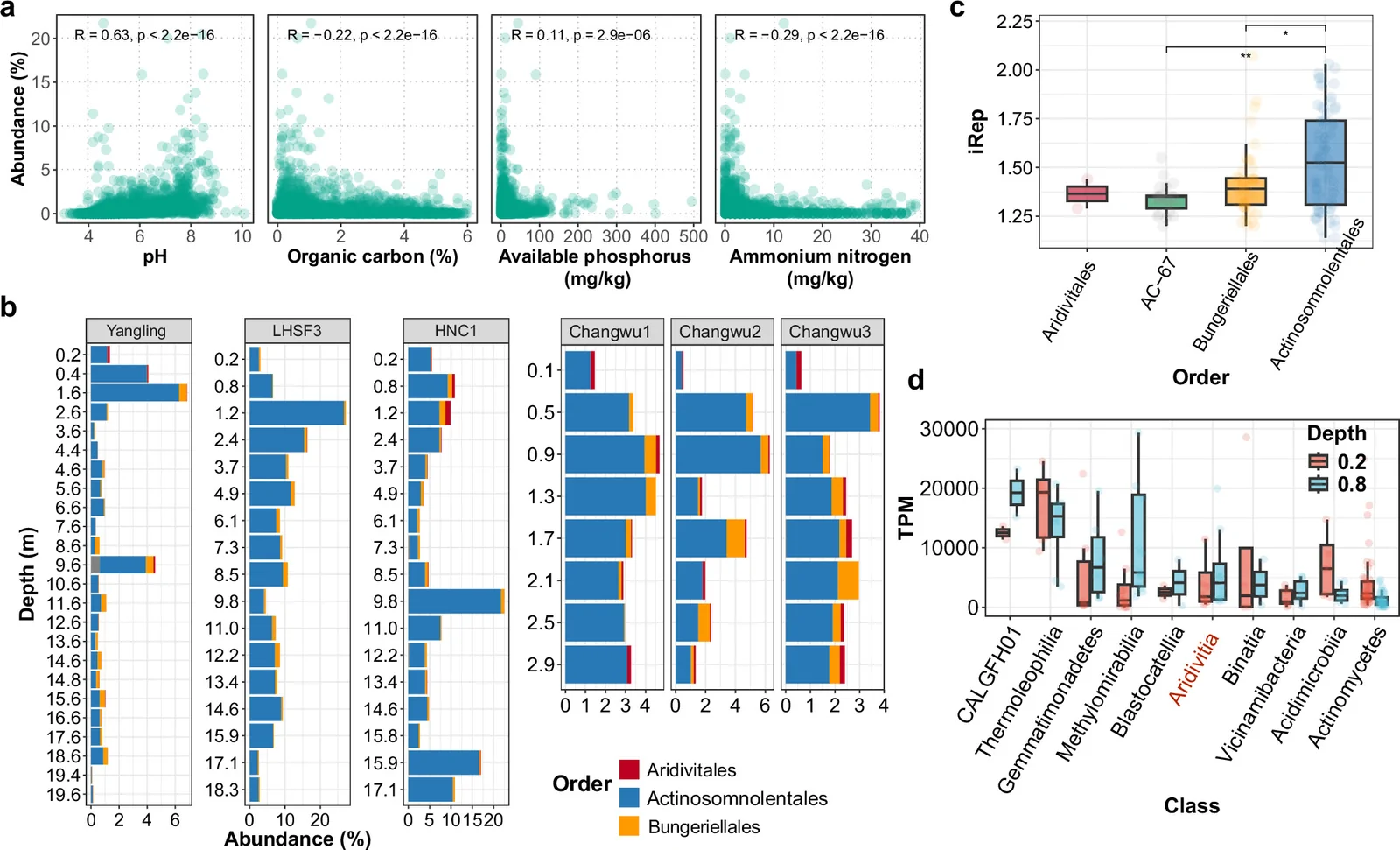
Predominance and high activity of Aridivitia in deep oligotrophic soils. **a** Spearman correlations between the relative abundance of Aridivitia and environmental factors across the Biomes of Australian Soil Environments (BASE) project. Statistical significance was assessed using a two-sided Spearman’s rank correlation test. Correlation coefficients (*R*) and exact *P* values are shown in the panels. **b** Relative abundance of Aridivitia across soil profiles from different sampling sites. Additional profiles are shown in Supplementary Fig. 5. **c** In situ replication index (iRep) across different orders within Aridivitia (*n* = 196 MAGs recovered in this study). Differences between groups were assessed using a two-sided Wilcoxon rank-sum test. Significant differences were observed between Actinosomnolentales and AC-67 (*P* = 0.00138) and between Actinosomnolentales and Bungeriellales (*P* = 0.0252). **d** Transcripts per million (TPM) of the ten most transcriptionally active microbial classes in surface (0.2 m) and deep (0.8 m) soil layers (*n* = 105 MAGs). In **c** and **d**, dots represent individual MAGs. Box plots show the median (center line), the 25th and 75th percentiles (box limits), and whiskers extending to 1.5 × the interquartile range. Source data are provided as a Source data file.

The relative abundance of Aridivitia lineages varies across soil types (Supplementary Fig. 1). To determine the environmental drivers, we analyzed two independent soil datasets from Australia^19^ and Europe^20^ (Supplementary Note S1). The results revealed an ecological preference of Aridivitia: its relative abundance was positively correlated with pH and significantly negatively correlated with organic carbon, ammonium nitrogen, and total nitrogen (all *p* < 0.001) (Fig. 2a, and Supplementary Fig. 3), suggesting that it is relatively more abundant in nutrient-poor, neutral-to-alkaline soils.

This oligotrophic preference was further supported by sampled soil profiles from China and the United States. In these profiles, the average organic content in surface soils was approximately 3.6-fold higher than in deep soils (Supplementary Fig. 4), while deep soils contained only 0.27 ± 0.10% organic carbon on average, indicating substantial resource limitation. Aridivitia occurred across all depths but showed clear enrichment in deep soils, reaching relative abundance up to 20% of total reads (Fig. 2b, and Supplementary Fig. 5). To assess whether this enrichment in deep soils is widespread, 1032 amplicon samples from soil profiles in eight independent studies were compiled and reanalyzed, also revealing consistently higher relative abundance in deep soils (Supplementary Data 2, Supplementary Fig. 6, and Supplementary Note S1). Beyond distribution, Aridivitia exhibited active in situ growth in deep soils. The average Index of Replication (iRep) calculated from metagenomic data was 1.46, suggesting that approximately half of Aridivitia cells were actively replicating (Fig. 2c). Metatranscriptomic analysis of the Yangling profile similarly placed Aridivitia among the top 10 most transcriptionally active lineages, with its median relative transcriptional contribution at 0.8 m being 2.3-fold higher than at 0.2 m (Fig. 2d). Together, these findings suggest that Aridivitia’s oligotrophic preference drives its relative abundance and activity in deep soils, supporting robust metabolic and proliferative capabilities.

### Genomic diversity and reductive evolution

**Fig. 3:**
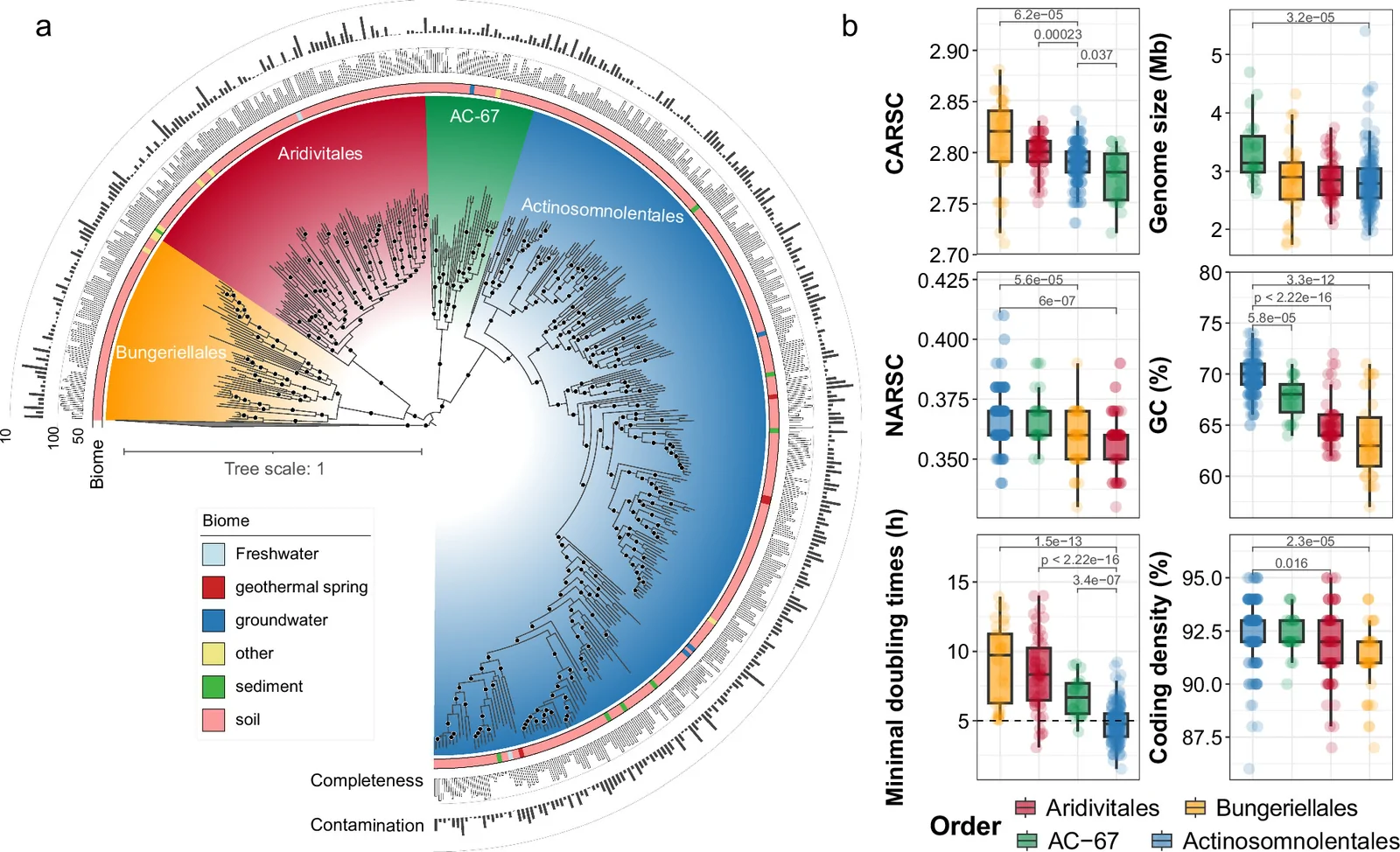
Phylogenetic diversity and genomic features of Aridivitia. **a** Phylogenetic tree of 423 species. The four orders are displayed in distinct colors, and each leaf contains annotations for its source biome, genome completeness, and contamination. **b** Genomic features among the four orders within Aridivitia, including estimated genome size, GC content, coding density, predicted minimum doubling time, carbon atoms per amino-acid-residue side chain (C-ARSC), and nitrogen atoms per amino-acid-residue side chain (N-ARSC). Dots represent individual MAGs (*n* = 254). Box plots show the median (center line), the 25th and 75th percentiles (box limits), and whiskers extending to 1.5× the interquartile range. Significant differences between groups were assessed using two-sided Wilcoxon rank-sum tests, with exact *P* values shown in the figure. Source data for (**b**) are provided as a Source data file.

We reconstructed 248 Aridivitia MAGs from soil profiles and integrated them with 710 public genomes, yielding the most comprehensive collection for this group to date (Supplementary Data 1, and Supplementary Note S1). Species-level dereplication produced 423 non-redundant representatives (85 from this study), averaging 83% completeness and 2.1% contamination, with 52 meeting the MIMAG high-quality standards (>90% completeness, <5% contamination, the presence of 5S, 16S, and 23S rRNA genes, and at least 18 tRNAs)^21^ (Supplementary Data 3). We constructed a phylogenetic tree based on 120 conserved bacterial single-copy marker genes to infer the phylogenetic placement of these species (Fig. 3a). Combined with average amino acid identity (AAI) and average nucleotide identity (ANI) (Supplementary Data 4 and 5), we resolved the phylogenetic affiliation and classification of this lineage. The 423 species (Supplementary Data 3) were classified into 4 orders, 12 families (2 novel), and 137 genera (12 novel).

Aridivitia genomes exhibited genomic features adapted to carbon-limited environments, including reduced genome sizes (2.89 ± 0.52 Mb), high GC content (68 ± 3%), and high coding density (Fig. 3b, and Supplementary Data 6). This average genome size is notably smaller than the predicted effective mean of ~4.74 Mb for soil bacteria^22^. Within this group, Actinosomnolentales dominates soil communities, accounting for an average of 87% of the total relative abundance (Fig. 2b, and Supplementary Fig. 7), and displays significantly higher iRep values and transcriptional activity than other orders (Fig. 2c, and Supplementary Fig. 8). Compared with other orders within Aridivitia, this dominant order has undergone genome reduction, featuring the smallest genomes, highest GC content and coding density, and amino-acid side-chain compositions that reduce carbon but increase nitrogen usage (Fig. 3b). Moreover, it is the only order within the group predicted to have rapid growth potential, with a minimal doubling time (MDT) of 4.8 ± 1.4 h (Fig. 3b, and Supplementary Data 6). Overall, these genomic features of Aridivitia suggest a high degree of adaptation to resource-limited soil habitats.

### Flexible strategies for carbon and energy acquisition in Aridivitia

**Fig. 4:**
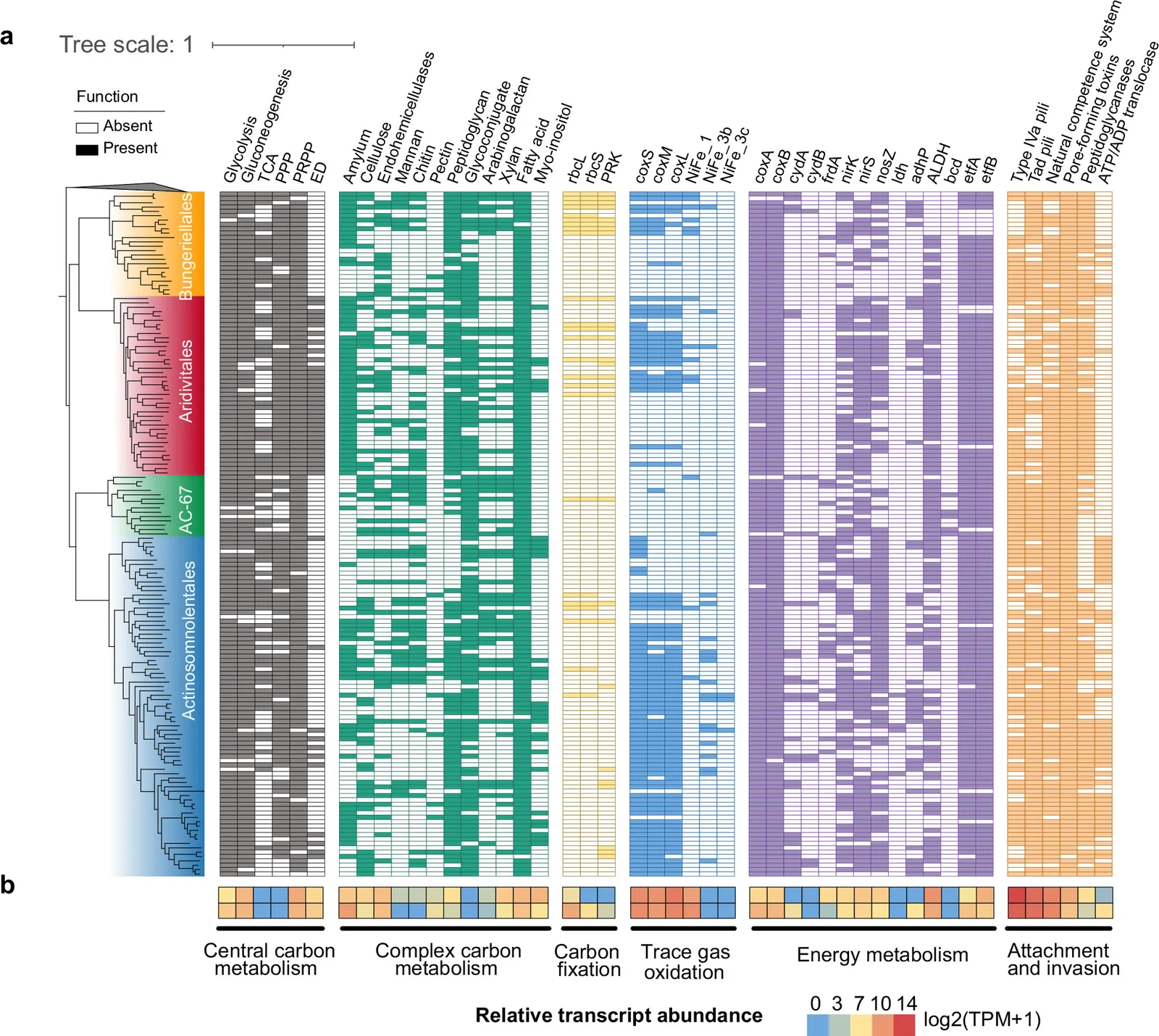
Metabolic potential and transcriptional activity of Aridivitia. **a** Distribution of functional genes and metabolic pathways across Aridivitia. The presence (colored) or absence (white) of pathways and marker genes involved in central carbon metabolism, complex carbohydrate degradation, carbon fixation, trace gas oxidation, energy metabolism, as well as attachment and invasion is shown. **b** Transcriptional expression levels of major metabolic pathways. The heatmap displays the relative transcript abundance of each functional module across two metatranscriptomic samples (0.2 m and 0.8 m).

Aridivitia MAGs encode relatively complete central carbon metabolic pathways, including glycolysis/gluconeogenesis, the tricarboxylic acid (TCA) cycle, the pentose phosphate pathway (PPP), and the phosphoribosyl pyrophosphate biosynthesis pathway, indicating broad carbon metabolic potential (Fig. 4a, and Supplementary Data 7). Their genomes encode diverse carbohydrate-active enzymes (CAZymes; average 57 per genome across 112 families), with glycoside hydrolases (GHs) being the most abundant,

suggesting the potential to degrade complex polysaccharides such as starch, cellulose, hemicellulose, mannan, and chitin (Fig. 4a, and Supplementary Data 8). The group also shows the potential to utilize proteinaceous substrates, encoding an average of 140 peptidase genes per MAG across 163 families (Supplementary Data 9). Nearly all genomes encode β-oxidation enzymes for fatty acid degradation, and inositol degradation genes were detected in the orders Actinosomnolentales and Aridivitales (Fig. 4a). Metatranscriptomic data indicated in situ expression of these pathways, underscoring the ability to utilize diverse organic compounds (Fig. 4b).

Some Aridivitia members encode the genomic potential for carbon fixation. RuBisCO (*rbcL*) for CO₂ fixation via the CBB cycle is encoded in 24 MAGs (Fig. 4a), primarily concentrated in the Bungeriellales and Aridivitales orders, and rarely present in the orders Actinosomnolentales and AC-67. Pathways for energy acquisition from inorganic gases are common in this group. Approximately two-thirds of the MAGs encode carbon monoxide dehydrogenase (*coxLMS*) for CO oxidation, showing high expression (Fig. 4b). In addition, 32 MAGs encode group 1, 3b, or 3c [NiFe]-hydrogenases for H₂ utilization (Fig. 4a, and Supplementary Data 11). Seven MAGs co-encode group 1h [NiFe]-hydrogenase (*hhyL*) and type IE RuBisCO (Supplementary Fig. 9, and Supplementary Data 11), suggesting the potential for atmospheric chemosynthesis by coupling atmospheric H₂ oxidation to CO₂ fixation^23,24^.

Regarding energy metabolism, nearly all Aridivitia MAGs encode cytochrome c oxidase (*coxA* and *coxB*), supporting aerobic respiration (Fig. 4a). Twenty-nine MAGs, primarily within Actinosomnolentales (21/29), encode high-affinity bd oxidases (*cydA* and *cydB*) for microoxic conditions. Beyond aerobic respiration, this lineage also exhibits a diverse capacity for anaerobic respiration using various terminal electron acceptors. The presence of fumarate reductase (*frdA*), nitrite reductases (*nirK* and *nirS)*, and nitrous oxide reductase (*nosZ*) indicates the potential to utilize fumarate, NO₂⁻, and N₂O as terminal electron acceptors (Fig. 4a). Members of this group may also acquire energy via fermentation. Lactate dehydrogenase (*ldh*) was identified in four species, while alcohol or aldehyde dehydrogenases (*adhP* and *ALDH*) were detected in 22 species, suggesting potential for lactate, other organic acid, or alcohol metabolism (Fig. 4a). Additionally, Bcd/EtfAB complexes were found in a subset of the AC-67 order (5/14), which may enhance energy efficiency under anaerobiosis (Fig. 4a). Aridivitia members also broadly encode diverse pathways involved in nitrogen and sulfur cycling (Supplementary Fig. 10, and Supplementary Note S2). In summary, these metabolic features suggest that Aridivitia possesses multiple flexible strategies for carbon and energy acquisition.

### Genomic evidence for a predatory lifestyle in Aridivitia

**Fig. 5:**
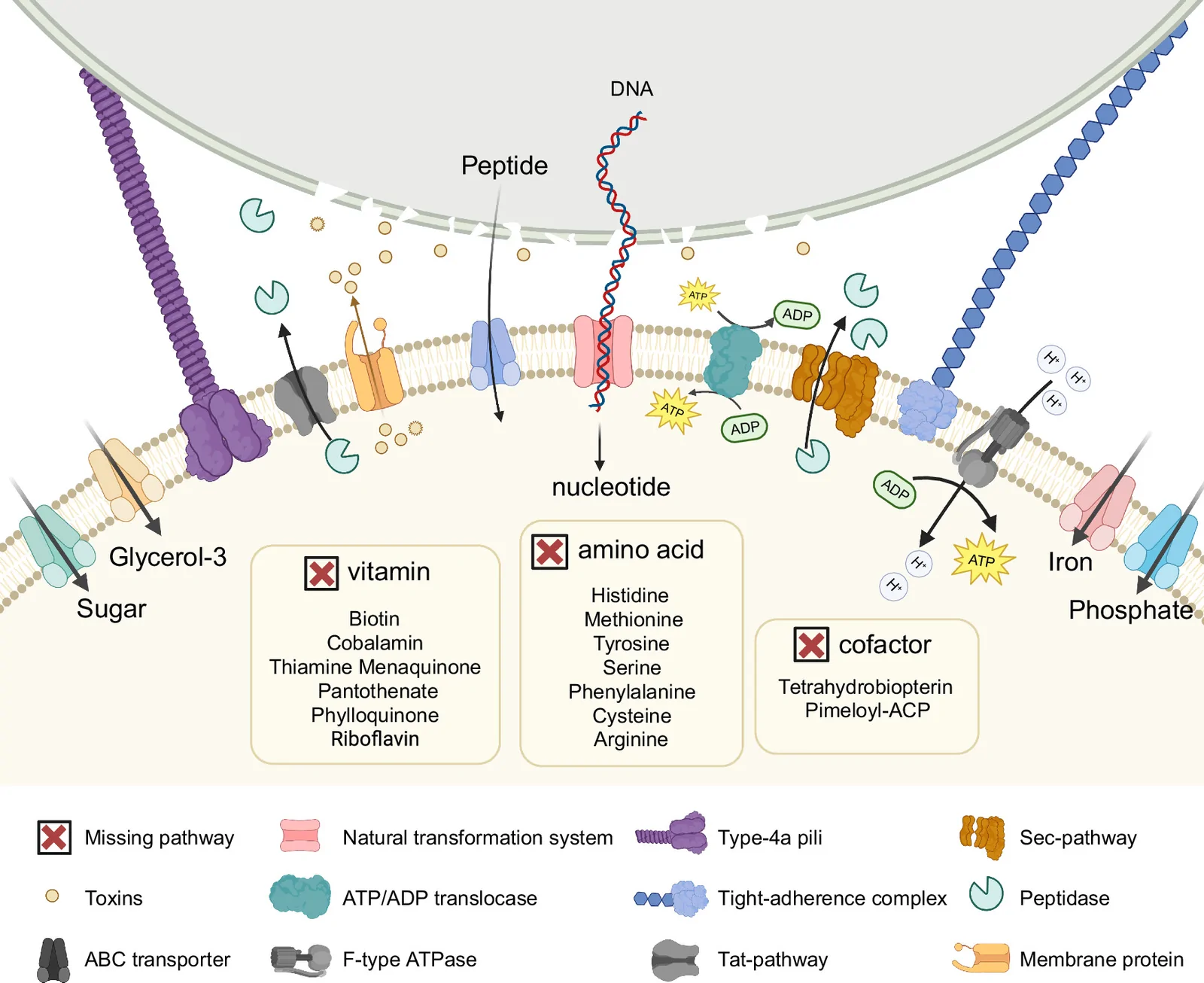
Summary of genomic evidence supporting a predatory lifestyle for Aridivitia. Aridivitia genomes combine hallmarks of host association (such as auxotrophies for vitamins, amino acids, and essential cofactors) with a suite of genes likely supporting a predatory lifestyle, including Type IVa and Tad pili for host cell attachment, pore-forming toxins, the Sec and Tat secretion systems, extracellular peptidases, ABC transporters, and ATP/ADP translocase. Created in BioRender. Wanxn, W. (2026) https://BioRender.com/mkn1jz3.

Aridivitia genomes encode cell surface structures consistent with a predatory lifestyle (Figs. 4a and 5, and Supplementary Data 12). Most species (108/157) encode type IVa pili gene clusters (Fig. 5). This pilus is a critical attachment structure used by predatory bacteria, such as *Bdellovibrio bacteriovorus*, to invade prey cells; strains lacking this structure fail to complete predation^25–27^. We also identified a complete Tad pili system (Fig. 5), which facilitates tight adherence to prey and contact-dependent killing^28,29^. Nearly all Aridivitia genomes encode the Sec secretion (Sec) and Twin-Arginine Translocation (Tat) systems, which mediate protein translocation across the cell membrane, potentially facilitating the export of predatory enzymes^30^ (Fig. 5). Regarding prey lysis, all members encode at least one family of pore-forming toxins (Hemolysin III, TlyA, or HP2-20) that facilitate prey cell lysis by forming membrane pores (Fig. 5). These toxins are also present in *Vampirococcus lugosii*^31^, a Candidate Phyla Radiation (CPR) bacterium with a predatory lifestyle, suggesting a potentially similar predatory strategy. Over 70% of species encode peptidoglycan lyases (Fig. 5), which directly degrade the bacterial cell wall. Furthermore, Aridivitia genomes encode a high density of secreted peptidases (average 7.8 per Mb), significantly exceeding the bacterial average (5.84 per Mb)^32^ (Fig. 5, and Supplementary Data 13), likely promoting the degradation of proteins released from lysed prey. This includes multiple copies of the M23 metalloprotease pseudoalterin in nearly all MAGs (Supplementary Data 13), which cleaves bacterial cell walls and likely contributes to feeding or defense^33^.

Tandem filtration of our soil samples showed that Aridivitia cells were predominantly retained on the 0.22 μm filter, indicating a cell size primarily within the 0.22–0.45 μm range, comparable to that of typical ultra-small microbes such as CPR and DPANN^34,35^ (Supplementary Fig. 11). Such ultra-small cells are commonly associated with dependence on other microorganisms for survival^36,37^. Consistent with this finding, Aridivitia MAGs exhibit a widespread absence of biosynthetic pathways for multiple essential molecules, including vitamins such as biotin and cobalamin, essential amino acids like methionine, histidine, and arginine, and crucial cofactors including pimeloyl-ACP and tetrahydrobiopterin (Fig. 5, Supplementary Fig. 12, and Supplementary Data 7). This suggests that Aridivitia must obtain these molecules from external sources, most likely prey cells, supporting its potential predatory lifestyle. Most Aridivitia MAGs encode diverse ABC transporters (Fig. 5, and Supplementary Data 12), likely enabling efficient uptake of nutrients released from lysed prey, including sugars, peptides, and phosphates. They also encode a complete natural competence system, facilitating efficient DNA uptake and integration (Fig. 5, and Supplementary Data 12). Notably, ATP/ADP translocases were predominantly identified in MAGs affiliated with Actinosomnolentales, with only rare occurrences in other orders (Fig. 5, and Supplementary Data 12). Given their established role in catalyzing transmembrane ATP/ADP exchange^38^, these enzymes may enable Actinosomnolentales to acquire energy resources from prey cells, potentially providing a key ecological advantage in energy-limited soil environments. We detected expression of key predation-associated genes in the Aridivitia metatranscriptome, including the type IVa pili, Tad pili, pore-forming toxins, and ATP/ADP translocases mentioned above (Fig. 4b). These findings provide evidence for molecular mechanisms of prey attachment, lysis, and invasion within this lineage and support potential specialized nutrient and energy acquisition strategies.

### Evolutionary transition from mixotrophy to predation in Aridivitia

**Fig. 6:**
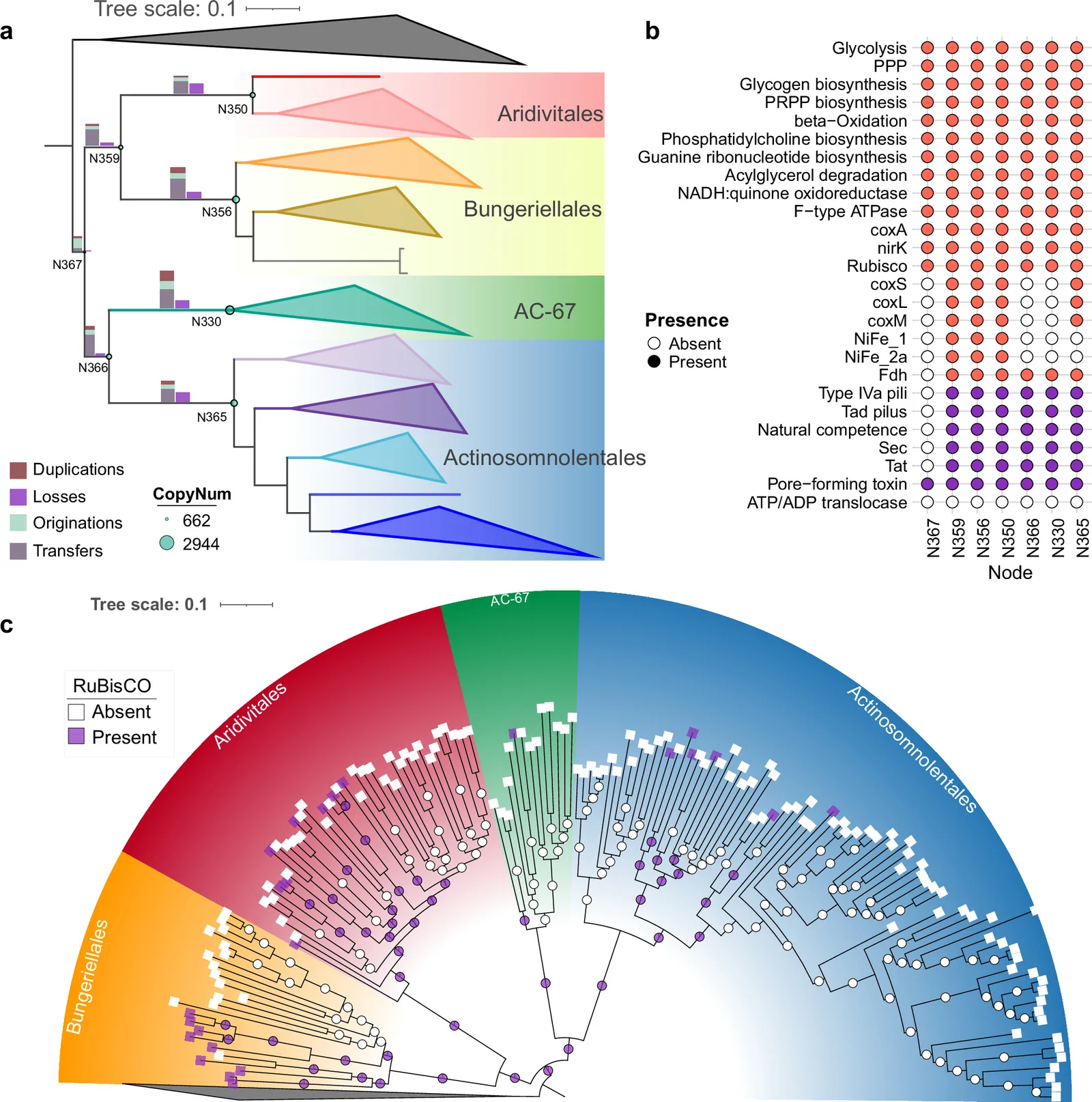
Ancestral reconstruction of gene families in Aridivitia. **a** Gene family evolutionary events at key early ancestral nodes include duplications, originations horizontal gene transfers (HGTs), and losses. Stacked bars along each branch represent the number of each event type, while the size of each node circle reflects the inferred total gene content of the ancestral genome. **b** Presence and absence of metabolic pathways and functional genes at key early ancestral nodes. Functional components related to potential predation or biotic interactions are highlighted in purple, while core and energy metabolism-related functions are highlighted in red. **c** Evolutionary history of ribulose 1,5-bisphosphate carboxylase/oxygenase (RuBisCO, *rbcL*) in Aridivitia. The tree shows the presence (filled shapes) or absence (open shapes) of RuBisCO at ancestral nodes (circles) and extant members (squares). Background colors represent different orders within the lineage.

We used comparative genomics to investigate the evolution of the putative predatory lifestyle in Aridivitia. To do so, we used the gene tree-species tree reconciliation tool ALE^39^ to reconcile gene family trees with the inferred species tree while accounting for gene tree uncertainty (from bootstrap replicates), thereby inferring gene origination, duplication, transfer, and loss (DTL) events as well as ancestral gene copy numbers at each node (Supplementary Data 14). The last common ancestor (LCA) of Aridivitia is inferred to have had a relatively small proteome, which significantly expanded during subsequent evolution (Fig. 6a, and Supplementary Fig. 13). Much of this expansion occurred along the stem lineages of the distinct orders within Aridivitia (Fig. 6a, and Supplementary Fig. 13), mediated both by de novo gene origination and the horizontal acquisition of genes. Quantification of gene content changes revealed 98,280 gene gains and 103,425 gene losses (Supplementary Data 14). Horizontal gene transfer (HGT) accounted for 75% of gene gains, with gene duplication and origination contributing 17% and 7%, respectively. Consequently, gene transfer and loss represent the major processes shaping genome evolution in Aridivitia (Fig. 6a, and Supplementary Fig. 13).

Ancestral genome reconstruction suggests that the LCA of Aridivitia was a free-living mixotrophic microorganism (Fig. 6b, and Supplementary Data 15). The LCA possessed complete core carbon pathways for heterotrophic metabolism, including glycolysis, the PPP, and gluconeogenesis, indicating that growth was primarily supported by exogenous organic carbon sources. Meanwhile, the presence of RuBisCO also suggests potential for CO₂ fixation (Fig. 6b). In terms of energy metabolism, the LCA was inferred to have possessed key components of oxidative phosphorylation, such as NADH:ubiquinone oxidoreductase and F-type ATP synthase (Fig. 6b). Flexible respiration is an ancient trait of the group, as cytochrome c oxidase (*coxA*) and nitrite reductase (*nirK*) are inferred to have been present in the earliest common ancestor (Fig. 6b). Although this ancestor carried pore-forming toxin genes, no structural components associated with adhesion or predation were detected. Thus, these toxins were more likely to function in competition or defense rather than active predation.

A pivotal ecological transition occurred during the diversification of the four ancestral orders. During this period, the clade simultaneously acquired Type IVa and Tad pili systems, natural competence machinery, and Sec/Tat secretion pathways (Fig. 6b), suggesting the emergence of predation- or invasion-related lifestyles. The acquisition of these traits was largely driven by gene origination events (Supplementary Fig. 14), which primarily reflect HGTs from lineages outside the group. The analysis also indicates that metabolic potential expanded concurrently during this stage, including the acquisition of genes for CO oxidation (*coxLMS*), H₂ utilization (hydrogenases), glycogen and amino acid degradation, and formate oxidation (*fdh*) (Fig. 6b), thereby enhancing the ability to derive energy from inorganic gases and degrade complex organic compounds. In subsequent evolution, further changes occurred. RuBisCO, which is inferred to have been widely present in early ancestral nodes, was progressively lost in most descendant lineages and retained only in a minority of extant members, primarily in the early-diverging orders Bungeriellales and Aridivitales (Fig. 6c). This reflects the loss of carbon fixation capability. Most RuBisCO-encoding MAGs (75%) lacked type IVa pili, indicating that lineages retaining autotrophy rarely possess the capacity for predation, suggesting an evolutionary trade-off between autotrophy and predatory traits. Notably, these early-diverging autotrophic lineages exhibited much lower relative abundance across soil datasets compared to the dominant order Actinosomnolentales with predatory traits (Supplementary Fig. 7), suggesting that the shift to predation may have contributed to the ecological success in oligotrophic soils. Additionally, genes encoding ATP/ADP translocases were absent across all early ancestral nodes and were primarily acquired after the divergence of Actinosomnolentales (Fig. 6b, and Supplementary Fig. 15). Together, these results reveal that a subset of lineages within Aridivitia underwent a lifestyle transition from mixotrophy to predation during the diversification of the group.

## Discussion

Deep soils constitute a vast oligotrophic “dark forest” within Earth’s Critical Zone that harbors diverse microbial life, whose survival and evolutionary strategies remain poorly understood. Here, we focus on Aridivitia, a previously overlooked bacterial lineage that is consistently enriched in deep soils and ranks among the most metabolically active groups in this habitat. This lineage provides a valuable model for understanding how microorganisms achieve ecological success under the chronic nutrient limitation of the terrestrial deep biosphere. Although this lineage is globally distributed, it exhibits a preference for oligotrophic soils. We reveal genomic features that support adaptation to such environments in this lineage, particularly in the dominant order Actinosomnolentales, which has undergone genome reduction. The reduced genomes, increased coding density, and ultra-small cell sizes lower non-essential metabolic costs and energy requirements during cell division and growth, resembling those observed in nutrient‑poor epipelagic marine and lacustrine microorganisms as well as CPR lineages^40–44^, suggesting convergent adaptation to energy-limited environments. However, unlike these aquatic oligotrophs that tend to have low GC content and increased proteomic C/N ratios, Actinosomnolentales exhibits high GC content and reduced proteomic C/N ratios alongside genome reduction. This difference reflects contrasting nutrient constraints, with aquatic systems generally being nitrogen-limited whereas soil microbial growth is predominantly carbon-limited^45^. Because GC base pairs have lower C/N ratios, selection for higher GC content and reduced proteomic C/N ratios can reduce cellular carbon demand^42,45^. Together, these optimizations provide the genomic basis for its persistence under nutrient-limited conditions.

Although genome reduction typically results in loss of coding potential⁴², members of Aridivitia exhibit metabolic flexibility, particularly in carbon acquisition and energy metabolism. They lost the capacity to biosynthesize various vitamins and energy-intensive amino acids while retaining the potential to degrade complex organic substrates such as carbohydrates, proteins, lipids, and inositol to capture carbon sources. This metabolic capacity suggests that Aridivitia may contribute to the slow transformation and turnover of buried complex organic carbon, including microbial necromass in deep soils, thereby influencing the long-term dynamics of organic carbon across soil profiles. In addition, their ability to obtain energy by oxidizing atmospheric trace gases (CO and H₂) provides a reliable alternative energy source for survival in energy-limited systems^23^. The expansion of substrate utilization and energy metabolism capabilities during evolution suggests that this metabolic flexibility has been favored by natural selection in resource-constrained environments, enabling flexible switching between organic and inorganic substrates and energy pathways, and thus thriving in resource-limited soils. Similar metabolic features also provide important support for the persistence of dominant microorganisms in other oligotrophic ecosystems, such as glacier forelands, Antarctic deserts, and permafrost^11,46,47^.

Our genomic and transcriptomic evidence further supports another important potential strategy underlying the success of Aridivitia in deep soils: attachment to and predation on other microorganisms. Similar to Bdellovibrio-and-like organisms, Aridivitia broadly encodes and expresses a predation-associated arsenal, including attachment structures, pore-forming toxins, and cell wall-degrading enzymes^33,48^. Successful predation generally requires motility, but rather than relying on flagellar swimming, members of Aridivitia employ type IVa pili-driven twitching, a surface-associated mode of movement better suited to solid environments such as soil^49^. The high in situ transcriptional activity of this lineage further supports a predatory lifestyle, as predatory bacteria in natural environments are typically highly metabolically active^50,51^. The ultra-small cell size of Aridivitia implies that its cells are typically smaller than those of its prey. This characteristic is common among bacterial predators, as a smaller size facilitates attachment to prey surfaces and enables efficient nutrient acquisition^42,52^. This putative predatory lifestyle was not an ancestral trait of the group, but instead emerged during adaptation to oligotrophy, as a free-living mixotrophic ancestor lost carbon fixation capacity while gaining and retaining adhesion and invasion capabilities. Although bacterial predation is often considered energy-intensive, this strategy is common in oligotrophic marine, groundwater and lake systems as well as soils, where the lysis of prey cells can provide predators with access to nutrients and energy that are otherwise scarce^53–55^. Accordingly, such a “hunting” strategy likely provides Aridivitia with an efficient means of acquiring limiting nutrients and energy from neighboring microorganisms, thereby contributing to its ecological success in nutrient-poor deep soils. It should be noted that, in the absence of direct microscopic observations of predator–prey interactions, the predatory behavior of Aridivitia and the identity of its potential prey remain to be experimentally validated. Given their broad distribution and non-specific metabolism, predation is likely opportunistic, targeting a wide range of co-occurring microorganisms in deep oligotrophic soils. Notably, members of the dominant order Actinosomnolentales within this group have evolved ATP/ADP translocases that enable the direct acquisition of ATP from prey cells. This distinctive energy-harvesting mechanism likely explains why this order maintains high metabolic activity, potential for rapid growth, and ecological dominance even under oligotrophic conditions. These findings suggest that extreme resource constraints do not always lead to functional simplification. Instead, they can facilitate the emergence of complex ecological traits, thereby providing alternative solutions for microbial flourishing at the energetic limits of life.

In summary, Aridivitia represents a specialized survival strategy among dominant soil oligotrophs. Its genome reduction, metabolic flexibility, and potential predatory lifestyle likely underpin its ecological success. Combined with its diverse metabolic potential for carbon, nitrogen, and sulfur, these features also suggest that Aridivitia may play important roles in subsurface energy and nutrient cycling, as well as in controlling microbial populations. Our findings advance our understanding of microbial persistence, metabolic activity, and evolution under extreme energy limitation. Given that genomic microdiversity can complicate metagenomic assembly and result in incomplete genomic reconstructions, MAG-based metabolic inferences may not fully capture the functional potential of this lineage^56,57^. Future targeted cultivation under both laboratory and in situ conditions, combined with direct microscopic observations, will help further validate these strategies, especially predation.

## Methods

### Sample collection

Soil samples were obtained from a total of ten soil profiles across Shaanxi, China, and Iowa, USA (Supplementary Fig. 16). These included one 20-m profile from Yangling and three 3-m profiles from Changwu in Shaanxi, China, as well as three 22-m profiles from Hitchcock Nature Center and three 22-m profiles from Loess Hills State Forest in Iowa, USA. Soil profiles in China were sampled at 0.2-m intervals, whereas those in Iowa were sampled at 1.22-m intervals. For each depth interval, a separate soil core was drilled, and only the central portion of the core was retained, discarding the top and bottom sections to minimize contamination. Each soil sample was divided into two portions: one was placed in an insulated container with ice packs and transported to the laboratory as soon as possible, while the other was immediately flash-frozen in liquid nitrogen on site for subsequent RNA extraction. Upon arrival at the laboratory, samples were stored at 4 °C, −20 °C, or −80 °C. More detailed soil sampling procedures were described in our previous study^58^.

### DNA extraction, amplicon and metagenomic sequencing, and data analysis

Total genomic DNA was extracted using the DNeasy PowerLyzer PowerSoil Kit (Qiagen, Germany), with blank extraction controls included to assess potential contamination (Supplementary Note S3, and Supplementary Fig. 17). The extracted DNA was subsequently sent to Biomarker Technologies (Beijing, China) for library preparation and sequencing. For amplicon sequencing, the V4 region of the 16S rRNA gene was amplified using primers 515F (5′-GTGYCAGCMGCCGCGGTAA-3′) and 806R (5′-GGACTACNVGGGTWTCTAAT-3′) for samples from China, whereas the V3–V4 region was amplified using primers 338F (5′-ACTCCTACGGGAGGCAGCA-3′) and 806R (5′-GGACTACHVGGGTWTCTAAT-3′) for samples from Iowa, USA. The amplified products were purified using magnetic beads, followed by PCR-based indexing to add sequencing adapters and sample-specific index sequences before sequencing on the Illumina NovaSeq 6000 platform (2×250 bp). For metagenomic sequencing, libraries were constructed using the VAHTS Universal Plus DNA Library Prep Kit for Illumina (Vazyme, ND617-02) and sequenced on the Illumina NovaSeq X Plus platform (2×150 bp). Amplicon sequences were processed using QIIME2 v2023.7^59^, with taxonomic assignment based on both the Greengenes 13.8 and Greengenes2 databases (to maintain consistency with the historical 0319-7L14 designation while also leveraging the updated taxonomy)^60,61^. For metagenomic analysis, sequencing generated an average of approximately 17 Gb of raw data per sample across 67 metagenomic libraries. Raw reads were quality-filtered using Trimmomatic v0.39^62^, with an average of 96% of paired-end reads retained. The quality-filtered reads were then assembled de novo using MetaSPAdes v3.13.0^63^. Contig binning and optimization were performed using the binning, bin_refinement, and reassemble_bins modules of MetaWRAP v1.3.0^64^. The quality of reconstructed MAGs was assessed using CheckM2 v1.0.2^65^, and only genomes of medium quality or higher (≥50% completeness and ≤10% contamination) were retained^21^. Taxonomic identification of MAGs was conducted using GTDB-Tk v2.4.0^66^. Recruitment rates of the recovered Aridivitia MAGs across all samples were calculated using CoverM v0.7.0^67^ (Supplementary Data 16).

### RNA extraction, metatranscriptomic sequencing, and data analysis

Total RNA was extracted using the RNeasy PowerSoil Total RNA Kit (Qiagen, Germany). The extracted RNA was subsequently sent to Majorbio (Shanghai, China), where rRNA was removed using the Ribo-Zero rRNA Removal Kit. The remaining RNA was fragmented and reverse-transcribed to generate double-stranded cDNA, followed by library construction using the TruSeq™ RNA Sample Prep Kit. Metatranscriptomic (2 × 150 bp) sequencing was performed on the Illumina HiSeq platform, generating approximately 11.3–11.6 Gb of raw data per sample across two metatranscriptomic libraries. Raw reads were quality-filtered and adapter-trimmed using Trimmomatic v0.39^62^, retaining approximately 90% of paired-end reads. Ribosomal RNA (rRNA) reads were subsequently removed using Ribodetector v0.3.2^68^, resulting in non-rRNA reads that accounted for approximately 60.0–66.9% of the raw reads. The resulting non-rRNA reads were aligned to MAGs using Bowtie2 v2.2.5^69^. Gene-level transcriptional activity was quantified using featureCounts^70^, with expression values normalized as transcripts per million (TPM). MAG-level transcriptional activity was estimated by summing reads mapped to all genes within each MAG and normalizing by MAG genome length.

### Environmental distribution and ecological drivers

To determine whether Aridivitia represents a geographically restricted lineage or a globally distributed microbial group, we first assessed its global distribution patterns using two large-scale resources: the EMP^17^ and Sandpiper^18^. For the EMP dataset, taxonomic assignment was performed using the Greengenes 13.8 database^60^; sequences classified as “o__0319-7L14” and their associated metadata were extracted to evaluate abundance and distribution frequency across diverse biomes. The Sandpiper dataset, which provides community composition estimates for global public metagenomes via SingleM^18^, was used to identify samples harboring Aridivitia. Detailed metadata for samples with an Aridivitia relative abundance exceeding 1% were retrieved using Kingfisher v0.4.1 (https://github.com/wwood/kingfisher-download).

After identifying its predominant association with soils, we further investigated the environmental factors associated with Aridivitia distribution using two large-scale soil 16S survey datasets: one from Europe^20^ and the other from the Biomes of Australian Soil Environments (BASE) project^19^. These datasets encompass broad geographic regions and provide comprehensive soil physicochemical measurements, including pH, organic carbon, nitrogen, and phosphorus, enabling analyzes of the associations between soil properties and Aridivitia relative abundance.

Additionally, to evaluate whether the deep-soil enrichment of Aridivitia is a widespread pattern, we downloaded and reanalyzed 16S rRNA gene datasets from eight independent soil profile studies, comprising a total of 1032 samples (Supplementary Data 2). All 16S rRNA gene sequences were processed and analyzed in accordance with the methodologies described above. An overview of all datasets used in this study is provided in Supplementary Note S1.

### Calculating the index of replication (iRep)

For the Aridivitia MAGs obtained in this study with completeness >75% and contamination <5%, in situ replication activity was assessed using iRep v1.10^71^. Metagenomic reads were aligned to each MAG using Bowtie2 v2.2.5 (--reorder)^69^ and the iRep value was calculated based on the decrease in coverage from the origin to the terminus of replication.

### Compilation of Aridivitia MAGs

To construct a genomic catalog for Aridivitia, we downloaded publicly available MAGs from IMG^72^, the SMAG catalogue^73^, GTDB^74^, GEM^75^, and the Tibetan Plateau Microbial Catalog^76^. These MAGs were taxonomically annotated using GTDB-Tk v2.4.0 (R220)^66^, and genomes identified as “c_UBA4738” were retained. The quality of the MAGs was assessed using CheckM2^65^, and 710 MAGs with ≥50% completeness and ≤10% contamination were selected for further analysis. These publicly sourced genomes were combined with 248 MAGs obtained in this study. Dereplication was conducted using dRep v3.2.2^77^ at a 95% ANI threshold, where the highest-quality representative from each redundant cluster was retained to construct a non-redundant genomic catalog comprising 423 Aridivitia MAGs. Potential rRNA genes in these MAGs were identified using Barrnap v0.9 (https://github.com/tseemann/barrnap), while potential tRNA genes were identified using tRNAscan-SE v2.0.12^78^.

### Phylogeny and taxonomy

Phylogenetic reconstruction was performed using the 423 representative MAGs of Aridivitia. Specifically, a set of 120 bacterial single-copy conserved marker genes (bac120) was identified, aligned using the identify and align modules of GTDB-Tk v2.4.0^66^. A maximum-likelihood phylogenetic tree was subsequently inferred using IQ-TREE v2.3.3^79^ under the best-fit model (parameters: -m MFP -B 1000 -alrt 1000). The resulting phylogenetic tree was visualized and annotated via iTOL^80^. Pairwise average AAI and ANI were calculated using CompareM v0.1.2 (https://github.com/dparks1134/CompareM) and FastANI^81^, respectively. Taxonomic ranks within Aridivitia were delineated by integrating GTDB taxonomy (R220), phylogenetic placement, and AAI/ANI values. In the current SeqCode Registry, the class Aridivitia^11^ contains three named orders: Aridivitales^11^ (later subjective synonym Actinopolaridales^82^), Bungeriellales^82^, and Actinosomnolentales^82^. These three lineages correspond to o__CADDZG01, o__JAHWKV01, and o__UBA4738, respectively, within c__UBA4738 in GTDB R220. Later GTDB releases revised the taxonomic circumscription of these clades; for example, in GTDB R232, the lineage corresponding to Actinosomnolentales was promoted to the class level, whereas Aridivitales and Bungeriellales were retained within c__CADDZG01 together with o__AC-67. In this paper, we refer to Aridivitia and this sister class collectively as the Aridivitia group, or simply Aridivitia.

### Genomic features

Genomic feature statistics were based on 254 MAGs with ≥80% completeness and ≤5% contamination. GC content and coding density were obtained from the outputs of CheckM2 v1.0.2^65^. The numbers of carbon atoms per amino-acid-residue side chain (C-ARSC) and nitrogen atoms per amino-acid-residue side chain (N-ARSC) were calculated for each genome using the script get_gc_and_narsc.py^83^. To account for genome incompleteness, total genome size was estimated as follows: Estimated size = (Total bp−(Total bp × contamination))/completeness. Microbial maximal growth rates were predicted from codon usage bias in genes encoding ribosomal proteins using gRodon^84^. Genes encoding ribosomal proteins were identified using Prokka v1.14.6^85^ and used together with the complete gene set as input for gRodon. The predictGrowth function was then used to calculate the MDT, with incomplete genomes set to mode = “partial.” Microorganisms with MDT < 5 h were considered fast-growing^84^.

### Genomic functional annotation

Protein-coding sequences (CDS) in the 157 MAGs of Aridivitia (completeness ≥90%) were predicted using Prodigal v2.6.3^86^. These CDS were annotated with KEGG Orthology identifiers using KofamScan v1.3.0 (--format mapper)^87^. METABOLIC v4.0^88^ was used to assess the completeness of the metabolic pathways in the MAGs. Carbohydrate-active enzymes (CAZymes) were identified using the dbCAN database^89^ with DIAMOND, HMMER, and dbCAN_sub. Peptidases in the genomes were identified by searching the MEROPS peptidase database^90^ using DIAMOND BLASTP (--more-sensitive -e 1e-10 --id 40 --subject-cover 70)^91^. Signal peptides in the coding sequences were detected using SignalP v6.0^92^ to identify potential secreted peptidases. Transport proteins were annotated by performing DIAMOND BLASTP^91^ (--more-sensitive -e 1e-10 --id 50 --subject-cover 70) searches against The Transporter Classification Database (TCDB)^93^. TXSScan^94^ was used to identify genes encoding T1SS–T6SS, T9SS, flagella, Type IV pili, and Tad pili. The presence of biosynthetic pathways for essential amino acids in each MAG was assessed using GapMind^95^. Potential RuBisCO encoding sequences were extracted from the METABOLIC v4.0^88^ analysis and merged with previously reported RuBisCO homologous sequences^24^. Multiple sequence alignment was performed using MAFFT v7.525^96^ with the L-INS-i algorithm. The alignment was then trimmed using TrimAl v1.4^97^ to remove poorly aligned or highly uncertain regions. A phylogenetic tree was constructed using IQ-TREE v2.3.3^79^ and visualized on the iTOL platform^80^. Hydrogenase protein sequences were classified in detail using the HydDB hydrogenase classifier^98^.

### Tandem filtration

To estimate the cell size of Aridivitia, we performed tandem filtration on two fresh soil samples collected from the Yangling profile (0.2 m and 8.6 m depth) and tracked the distribution of Aridivitia across size-fractionated microbial communities. Briefly, 15 g of fresh soil was transferred into a sterile Erlenmeyer flask containing 5 g of sterile glass beads and 100 mL of phosphate-buffered saline (PBS, pH 7.4). The suspension was shaken at 180 rpm for 1.5 h at 4 °C in the dark to facilitate the dissociation of microorganisms attached to soil particle surfaces. After settling for 10 min, the supernatant was collected and subjected to intermittent ultrasonication in an ice bath (20 kHz; 1-min on/off cycles, repeated four times) to further disrupt microbial aggregates. The mixture was then centrifuged at 1000 × *g* for 15 min at 4 °C to remove residual soil particles. The resulting supernatant was sequentially filtered through a series of sterile polycarbonate membranes with decreasing pore sizes (2, 0.8, 0.45, and 0.22 µm). Microbial DNA was extracted from each filtration fraction using the DNeasy PowerLyzer PowerSoil Kit (Qiagen, Germany), and the V4 region of the 16S rRNA gene was amplified and sequenced following the bioinformatic pipeline described previously.

### Ancestral reconstruction

Orthologous groups (OGs) were identified using OrthoFinder v2.5.5^99^ for 157 high-quality genomes of Aridivitia (with completeness ≥90% and contamination ≤5%). Only OGs containing at least four sequences, each exceeding 30 amino acids in length, were retained, resulting in a total of 8410 OGs. MAFFT v7.49^96^ was used to perform multiple sequence alignment for each OG, and low-quality regions were automatically trimmed using TrimAL v1.4^97^. Individual gene trees were inferred using IQ-TREE v2.3.3^79^, with the best-fit model automatically selected by ModelFinder (-m MFP). For each gene family, 1000 ultrafast bootstrap (UFB) replicates were generated (-B 1000 -alrt 1000). A species tree for the 157 high-quality Aridivitia genomes was reconstructed based on 120 bacterial single-copy conserved marker genes, following the same pipeline described previously for phylogenetic construction. ALEobserve was used to compute conditional clade probabilities from the ultrafast bootstrap replicate trees for each of the 8410 gene families. Gene family evolution was then reconciled with the species tree using ALEml_undated, which jointly accounts for gene tree uncertainty and models gene DTL^39^. The fraction_missing parameter for each genome was set based on CheckM2 completeness estimates to correct for genes absent due to incomplete genome assembly rather than true gene loss. Ancestral metabolic reconstruction was performed based on the inference, annotation, and copy number of genes at ancestral nodes. A gene was considered present at an ancestral node if its inferred copy number exceeded a threshold of 0.3.

### Statistics & reproducibility

No statistical method was used to predetermine sample size. Data failing standard quality control were excluded. Randomization and blinding were not applicable because this study was based on environmental samples collected across field locations and soil depths.

### Reporting summary

Further information on research design is available in the Nature Portfolio Reporting Summary linked to this article.

## Supplementary information

Supplementary Information

Description of Additional Supplementary Files

Supplementary Data 1-16

Reporting Summary

Transparent Peer Review file

## Source data

Source Data

## Data Availability

The raw sequencing data generated in this study have been deposited in the NCBI Sequence Read Archive (SRA) under the BioProject accession number PRJNA1417468. The MAGs affiliated with Aridivitia generated and analyzed during this study are publicly available on Figshare with the identifier https://doi.org/10.6084/m9.figshare.31046038. All other data supporting the findings of this study, including Supplementary Data, figures, and other supporting information, are included in the published article and/or its supplementary materials. Source data are provided with this paper.
