# Supplementary material for: Genome-reduced Aridivitia evolved predation to thrive in Earth’s oligotrophic depths
Source: Nat Commun. 2026 Oct 14;17:11624. doi: 10.1038/s41467-026-78342-x (PMC13755416; doi:10.1038/s41467-026-78342-x)
Supplement: Supplementary file 1 [file 41467_2026_78342_MOESM1_ESM.pdf]

Supporting Information for

Genome-reduced Aridivitia evolved predation to thrive in Earth's oligotrophic depths

Authors: Xiaonan Wan[1, 2, 3], Wenlu Feng[1, 2], Yang Zhang[1, 2], Tom A. Williams[4], Gergely J. Szöllősi[5, 6], Hongyu Dang[1, 2], Xuejun Yan[1, 2, 3*], James M. Tiedje[2, 7], Jizhong Zhou[8] and Xun Qian[1, 2, 3*]

1. College of Natural Resources and Environment, Northwest A&F University, Shaanxi 712100, China

2. Key Laboratory of Plant Nutrition and Agro-environment in Northwest China, Ministry of Agriculture and Rural Affairs, Northwest A&F University, Shaanxi 712100, China

3. State Key Laboratory of Soil and Water Conservation and Desertification Control, Northwest A&F University, Yangling, Shaanxi 712100, China

4. Department of Life Sciences, University of Bath, Bath, UK

5. Model-Based Evolutionary Genomics Unit, Okinawa Institute of Science and Technology Graduate University, Okinawa, Japan

6. Institute of Evolution, HUN-REN Center for Ecological Research, H-1121 Budapest, Hungary

7. Center for Microbial Ecology, Michigan State University, East Lansing, MI 48824, USA

8. Institute for Environmental Genomics, University of Oklahoma, Norman, OK 73019, USA

***Corresponding author**: yanxuejun@nwafu.edu.cn (X. J. Yan); qianxun@nwafu.edu.cn (X. Qian)

Supplementary Notes

Note S1: Overview of datasets used in this study

In this study, we integrated newly generated data from our own sampling efforts with multiple publicly available resources to investigate the global distribution, environmental associations, vertical distribution, and genomic strategies of the Aridivitia group.

To determine whether Aridivitia represents a geographically restricted lineage or a globally distributed microbial group, we assessed its global distribution patterns using two large-scale resources: the Earth Microbiome Project (EMP)[1] and Sandpiper[2]. The EMP is an international open-science microbiome initiative aimed at characterizing and understanding microbial diversity across the Earth at a global scale. It integrates 27,751 16S rRNA gene samples from 97 studies spanning seven continents, 43 countries, and 17 environmental categories. Sandpiper is an online microbial community analysis platform developed by Queensland University of Technology, Australia, which provides microbial community composition profiles derived from 248,559 publicly available metagenomes covering 217 habitat types, including marine, lake, freshwater, soil, hot spring, and wetland environments. These datasets revealed that Aridivitia is widely distributed worldwide and is primarily associated with soil habitats.

After confirming its preference for soils, we further investigated the environmental factors associated with Aridivitia distribution using two large-scale soil 16S survey datasets. The first was the European soil dataset organized by the Joint Research Centre (JRC) of the European Commission[3], which covers 715 sampling sites across 24 European countries, with sampling depths 0–20 cm. The second was the Biomes of Australian Soil Environments (BASE) dataset[4], a nationwide soil microbiome survey across Australia comprising more than 1,400 samples collected from over 900 locations at depths of 0–30 cm. Both datasets provide 16S rRNA gene data together with comprehensive soil physicochemical measurements, enabling analyses of the associations between environmental factors and Aridivitia relative abundance. These analyses revealed that Aridivitia is significantly associated with soils containing lower organic carbon content.

Consistent with this preference for low-organic-carbon soils, we observed that Aridivitia is enriched in deeper, carbon-poor soil horizons in the profiles collected in this study. These profiles included one 20-m profile from Yangling and three 3-m profiles from

Changwu, China, as well as three 22-m profiles from Hitchcock Nature Center and three 22-m profiles from Loess Hills State Forest, USA. These profiles generated 16S rRNA gene amplicon, metagenomic, metatranscriptomic, and soil physicochemical data. To evaluate whether this deep enrichment is a general phenomenon, we additionally downloaded and reanalyzed 16S rRNA gene datasets from eight independent soil profile studies, comprising a total of 1,032 samples (Supplementary Table 2). The results consistently showed that Aridivitia is also relatively more abundant in the deep layers of these independent profiles.

To understand how Aridivitia successfully colonizes and maintains predominance in oligotrophic environments such as deep soils, we comprehensively analyzed the 248 MAGs recovered in this study together with publicly available genomes of this lineage. These genomic resources were used to investigate phylogenetic relationships, genomic characteristics, metabolic potential, and evolutionary history.

Note S2: Genomic potential for nitrogen and sulfur cycling in Aridivitia

Genomes of Aridivitia encode diverse genes involved in nitrogen and sulfur cycling (Supplementary Fig. 10). In nitrogen metabolism, their genomes consistently encode a complete assimilatory nitrate reduction pathway capable of reducing nitrate (NO_3^-) to ammonium (NH_4^+). Some members contain genes for dissimilatory nitrite reduction (*nirBD*), which facilitates the conversion of nitrite (NO_2^-) to ammonia (NH_3). In sulfur metabolism, Aridivitia genomes commonly encode pathways for assimilatory sulfate reduction (*sat*) as well as genes involved in dissimilatory sulfite reduction (*sir*), potentially leading to hydrogen sulfide (H_2S) production. Sulfur oxidation potential is indicated by the presence of *sqr*, which catalyzes the oxidation of H_2S to elemental sulfur.

Note S3: Controls for DNA extraction and assessment of potential contamination

To assess potential contamination during DNA extraction, blank controls were included during the DNA extraction process. These controls were processed using the same DNeasy PowerLyzer PowerSoil Kit protocol as the soil samples but without soil input. DNA concentration was measured using the Qubit dsDNA High Sensitivity Assay Kit (Thermo Fisher Scientific), and all blank controls were below the detection limit (<0.005 ng/μL). The V4 region of the bacterial 16S rRNA gene was amplified using primers 515F (5′ -

GTGYCAGCMGCCGCGGTAA-3′) and 806R (5′-GGACTACNVGGGTWTCTAAT-3′). Agarose gel electrophoresis of the blank controls and deep-soil DNA samples showed no detectable amplification products in any blank controls (Supplementary Fig. 17). Quantitative PCR targeting the same 16S rRNA gene region further confirmed that no amplification signal was detected in any blank controls (Cq = NA). Additionally, the blank controls were processed together with soil DNA extracts and sent to Biomarker Technologies (Beijing, China) for library preparation and sequencing. The DNA concentrations of the blank controls were below the requirements for library construction, and no sequencing libraries were generated.

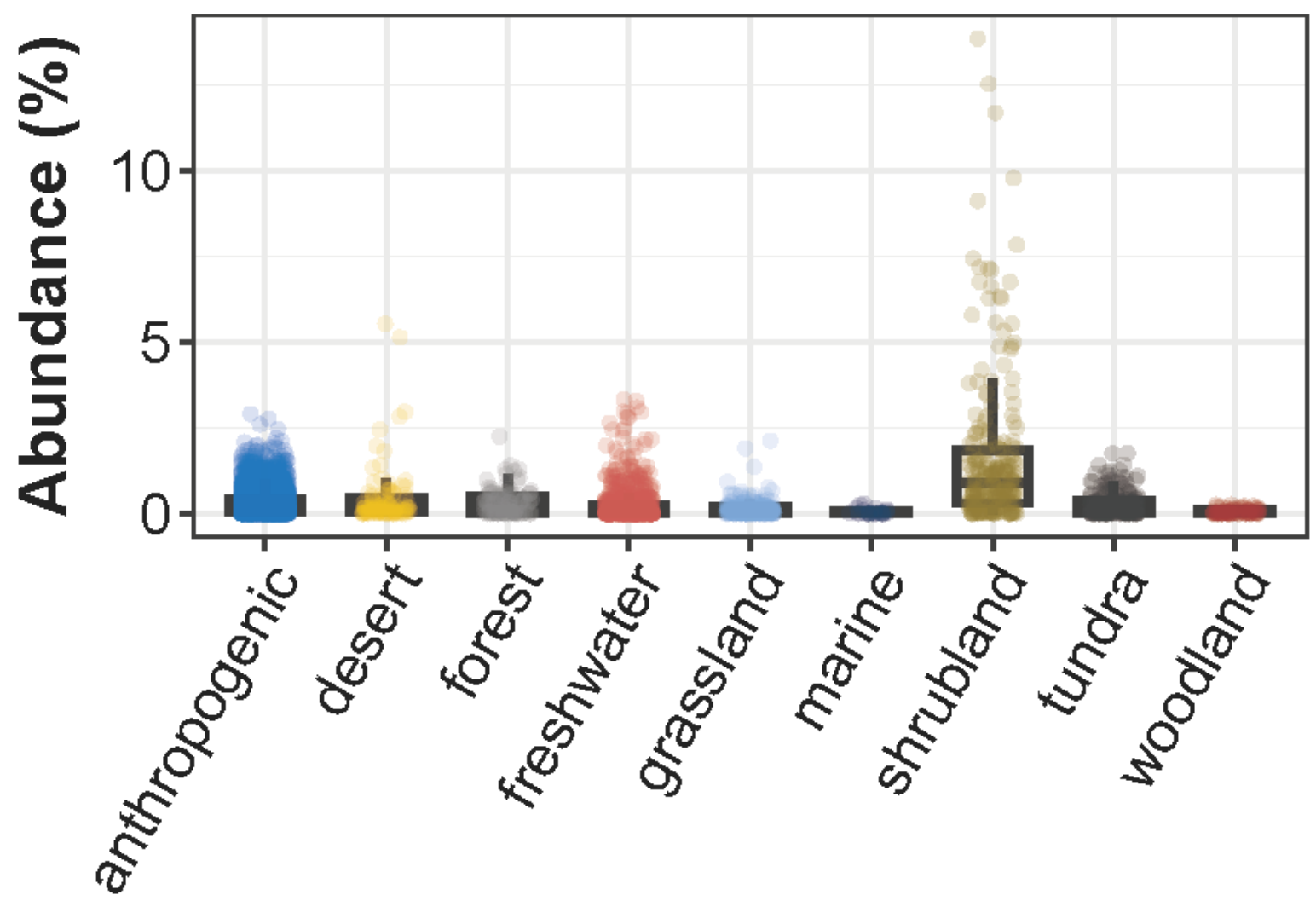

Supplementary Fig 1. Relative abundance of the Aridivitia group in different habitat types of the EMP dataset. Relative abundance was calculated as the proportion of reads assigned to Aridivitia within each sample (n = 5,161 samples). Source data are provided as a Source data file.

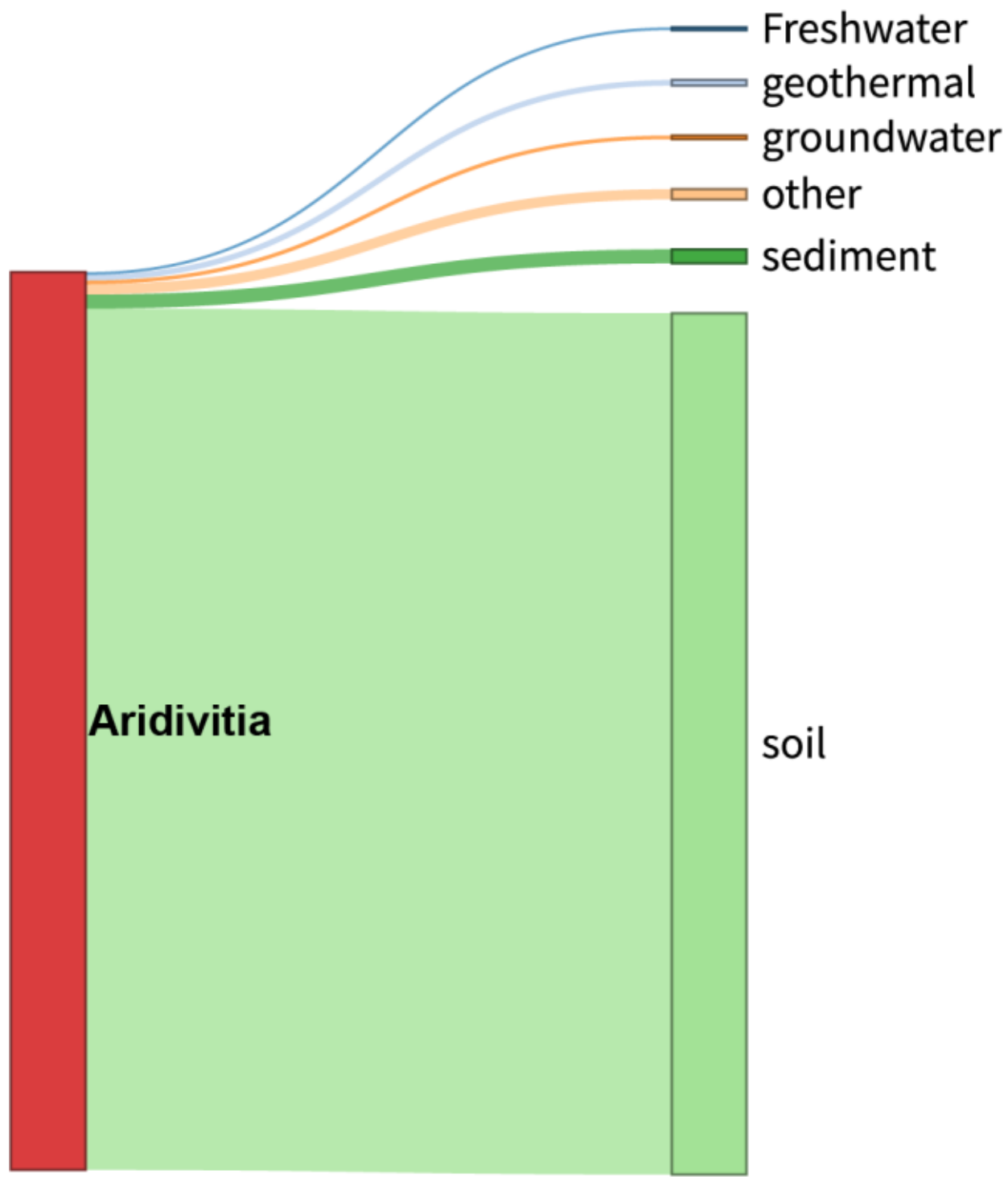

Supplementary Fig 2. Habitat distribution of the Aridivitia genomic collection. Sankey diagram showing that the vast majority of Aridivitia MAGs are derived from soil, with others recovered from freshwater, geothermal, groundwater, and sediment environments.

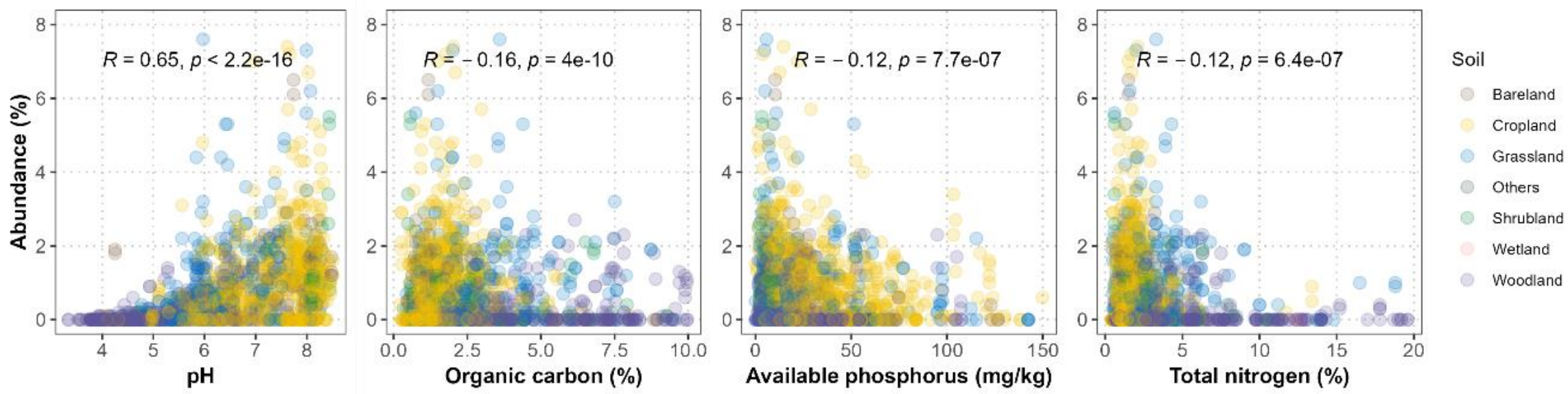

Supplementary Fig 3. Spearman correlations between Aridivitia relative abundance and environmental factors across the European dataset. Points are color-coded by soil habitat type (n = 1,029 samples). Statistical significance was assessed using a two-sided Spearman's rank correlation test. Correlation coefficients (R) and exact P values are shown in the panels. Source data are provided as a Source data file.

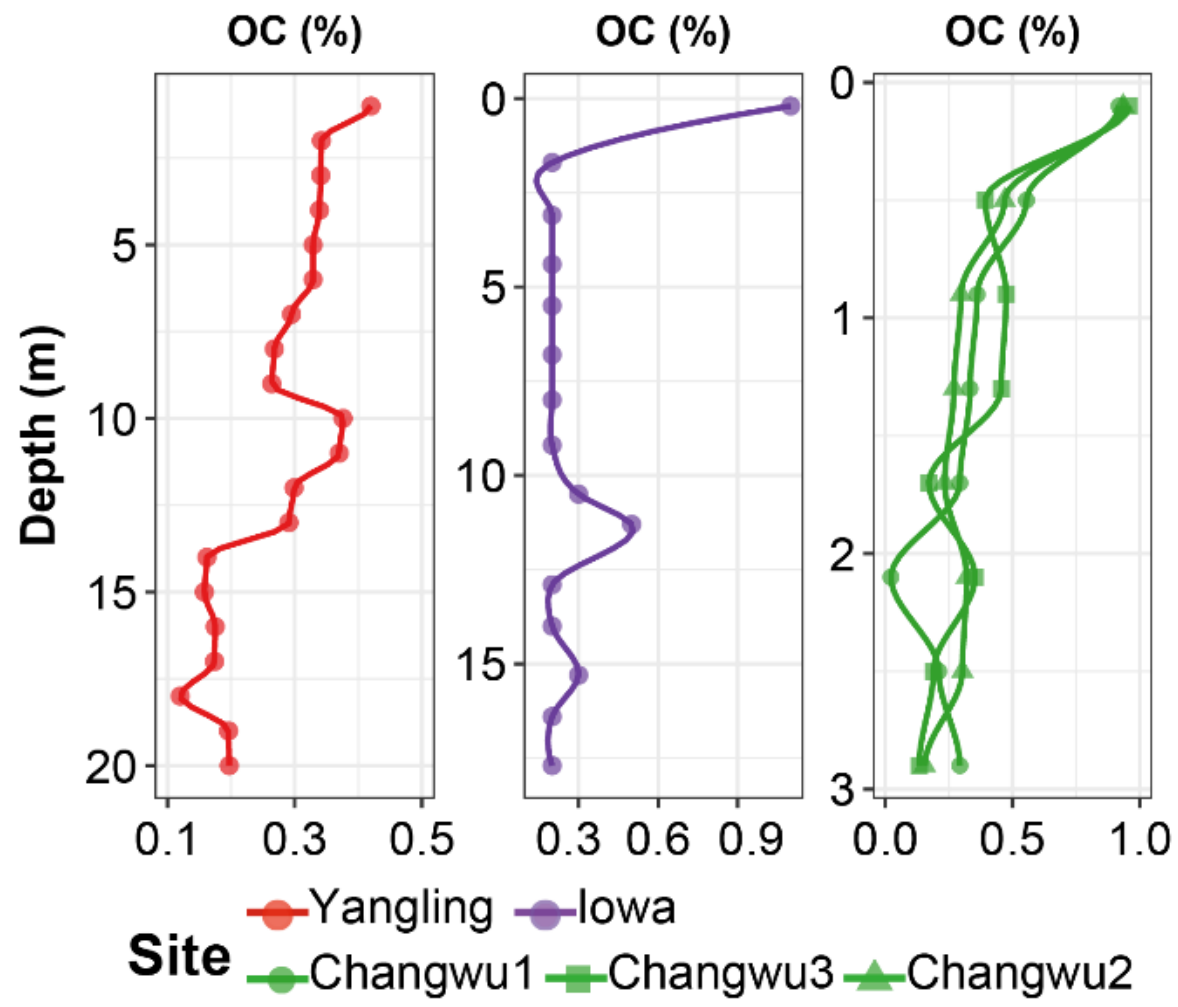

Supplementary Fig 4. Vertical distribution of total organic carbon (OC) content across the sampled soil profiles. Detailed site descriptions and methodologies are available in Feng et al. (2025)[5]. Source data are provided as a Source data file.

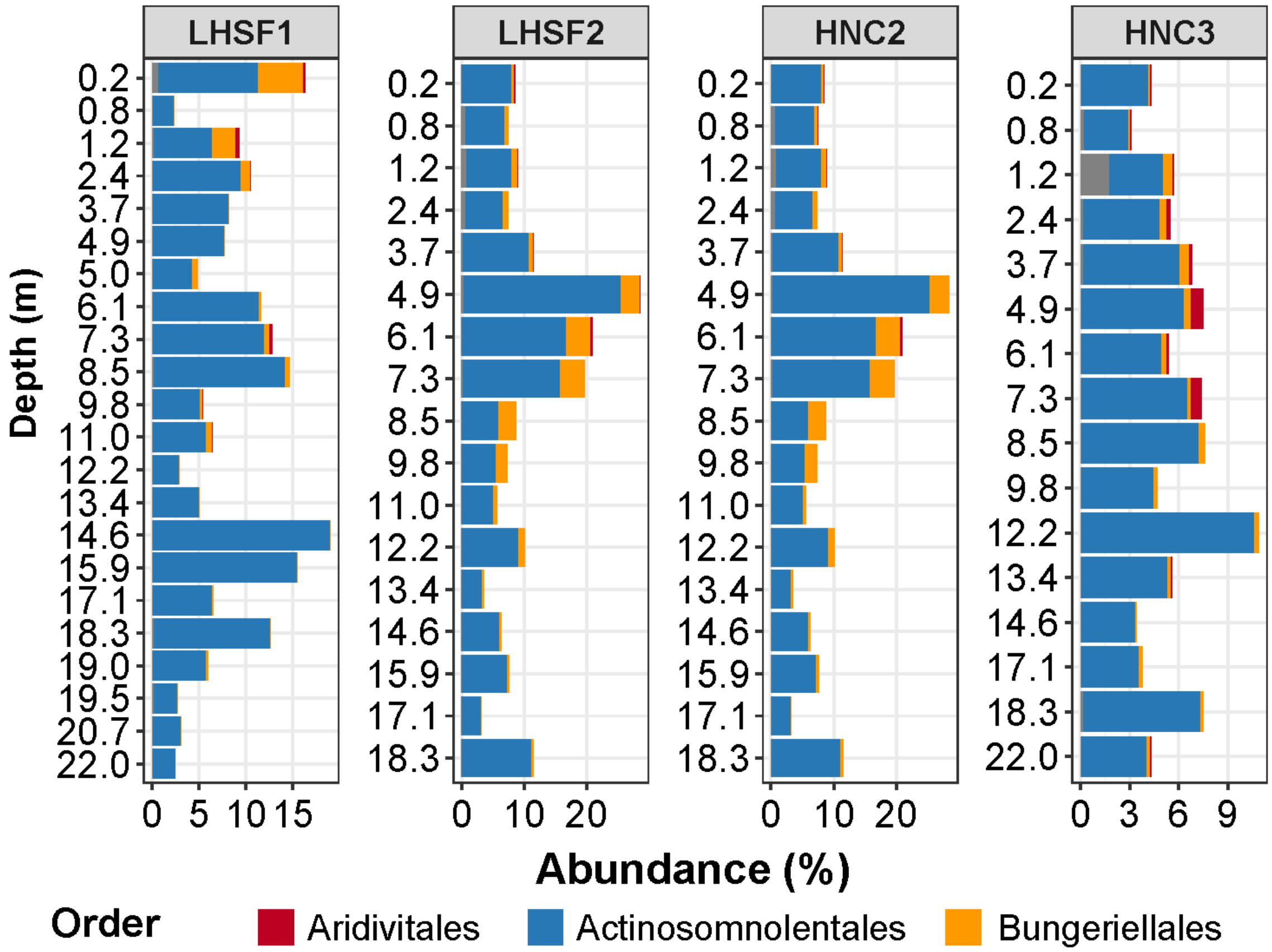

Supplementary Fig 5. Relative abundance of Aridivitia across additional soil profiles from USA sampling sites (LHSF1, 2 and HNC2, 3). Source data are provided as a Source data file.

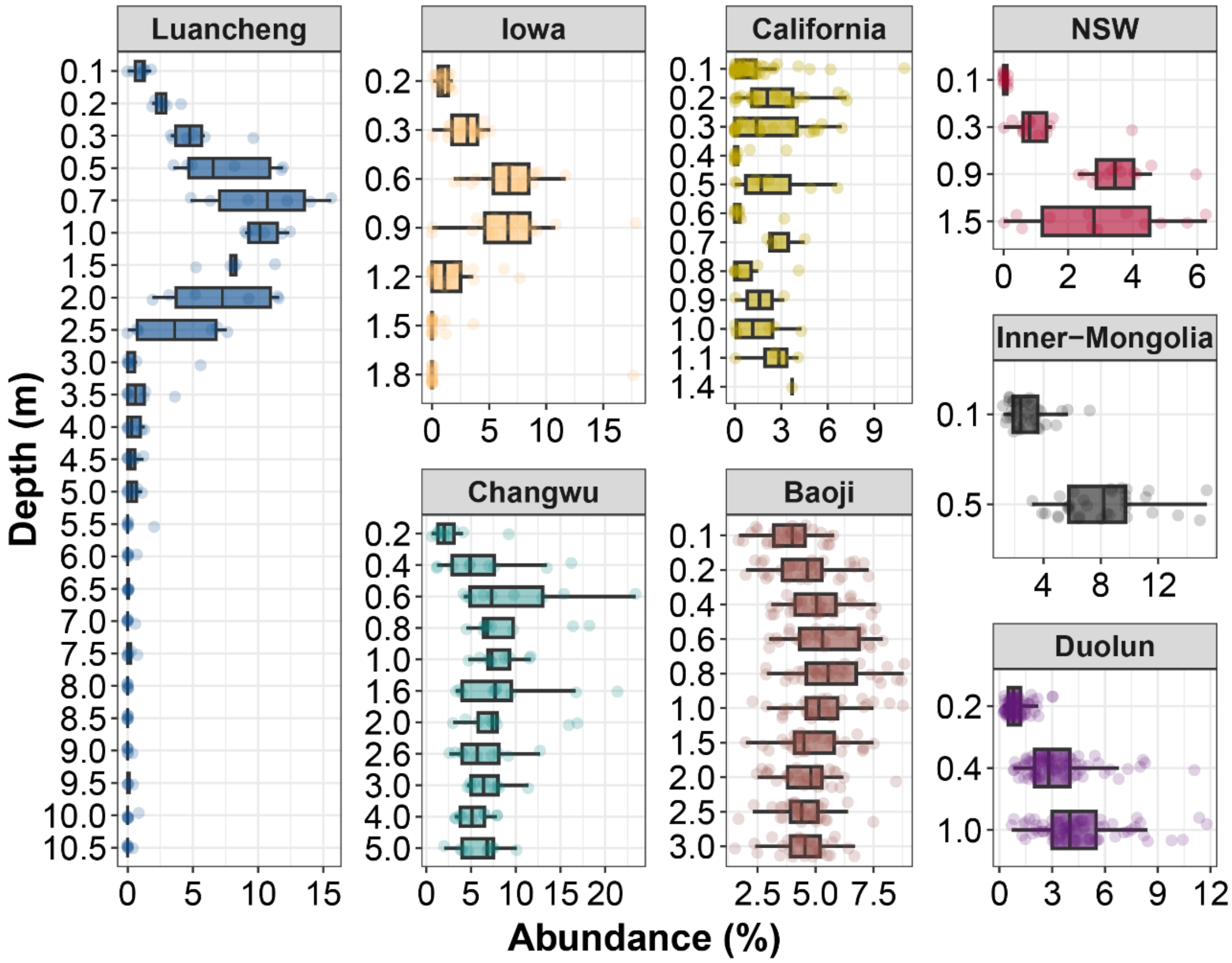

Supplementary Fig 6. Abundance distribution of Aridivitia across soil profiles from multiple published studies. Relative abundance of Aridivitia across soil profiles integrated from eight independent studies (n = 1,032 samples). Box plots show the median, 25th and 75th percentiles, with whiskers extending to the most extreme values within 1.5 × the interquartile range. Detailed information regarding sampling sites, geographic locations, and original data sources is provided in Supplementary Table 2. Source data are provided as a Source data file.

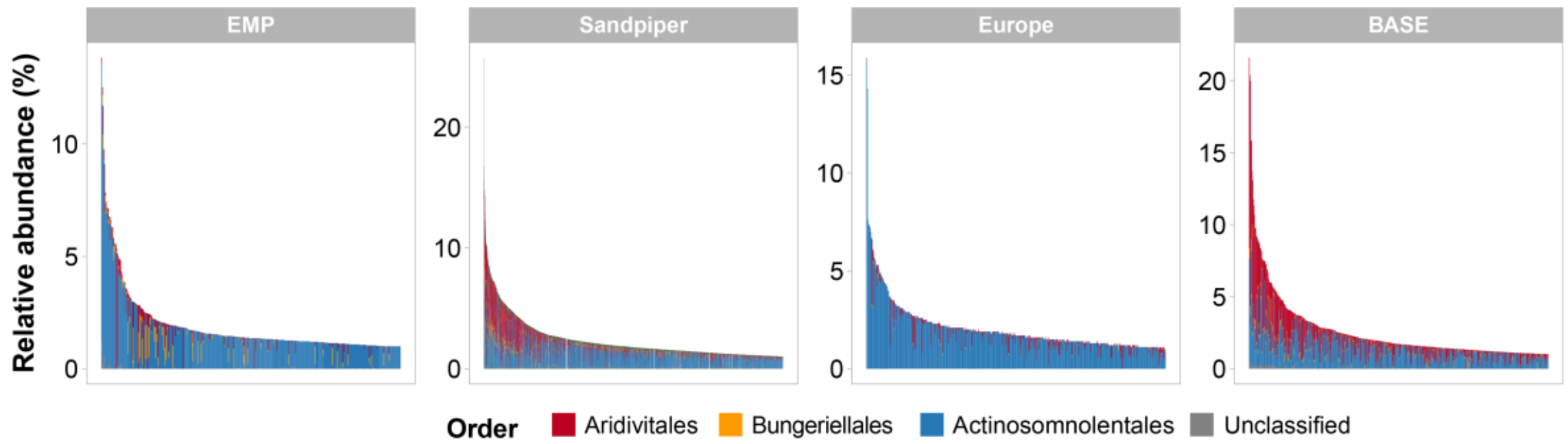

Supplementary Fig 7. Composition of orders within Aridivitia across diverse datasets. Relative abundance of orders within Aridivitia across soil samples from the EMP, Sandpiper, Europe, and BASE datasets. Each bar represents the taxonomic composition within an individual sample. Source data are provided as a Source data file.

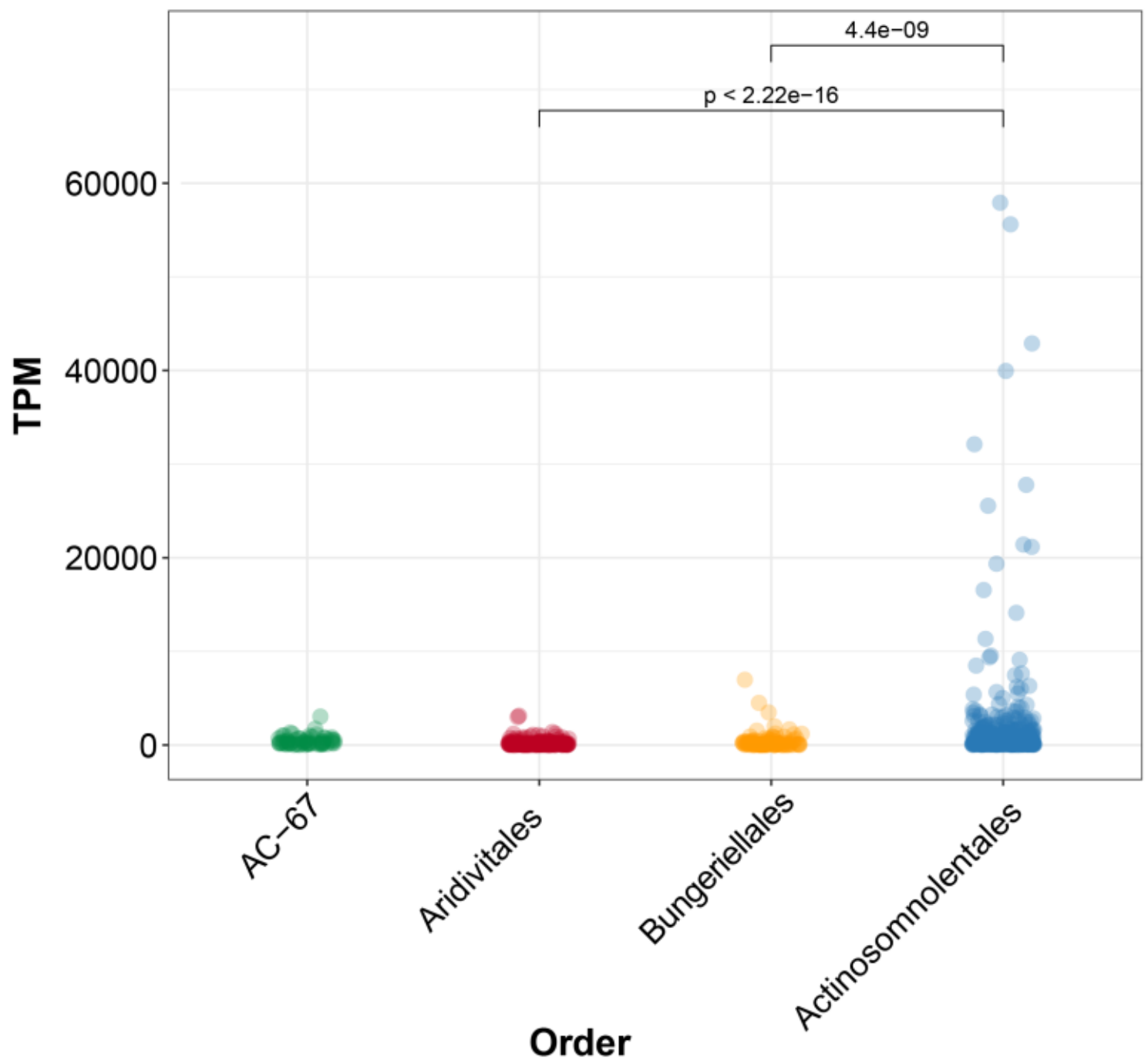

Supplementary Fig 8. Comparison of transcriptional activity across orders within Aridivitia. Transcriptional levels (TPM, Transcripts Per Million) of MAGs belonging to different orders (AC-67, Aridivitales, Bungeriellales, and Actinosomnolentales) within Aridivitia, as assessed by metatranscriptomic data. Each dot represents the transcriptional activity of an individual MAG (n=852). Statistical significance was assessed using two-sided Wilcoxon rank-sum tests, with exact P values shown in the figure. Source data are provided as a Source data file.

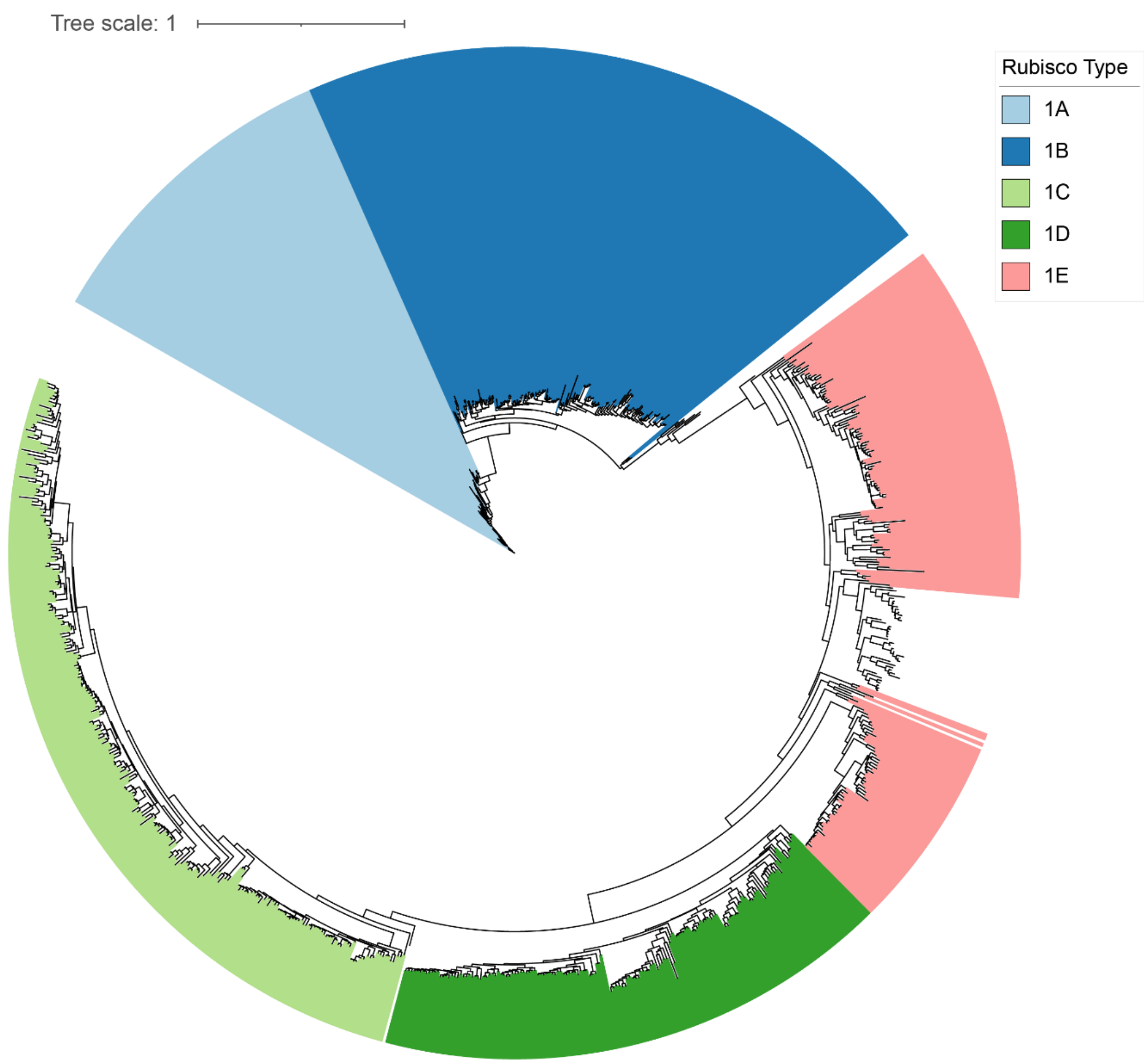

Supplementary Fig 9. Phylogenetic analysis of RuBisCO large subunit sequences in Aridivitia. Maximum-likelihood phylogenetic tree of RuBisCO sequences retrieved from Aridivitia genomes and known reference homologs. Colored clades indicate established Form I RuBisCO subtypes (1A, 1B, 1C, 1D, and 1E) from reference datasets, while uncolored (black) branches represent sequences from Aridivitia. The results demonstrate that RuBisCO sequences from Aridivitia primarily cluster within the Form 1E clade.

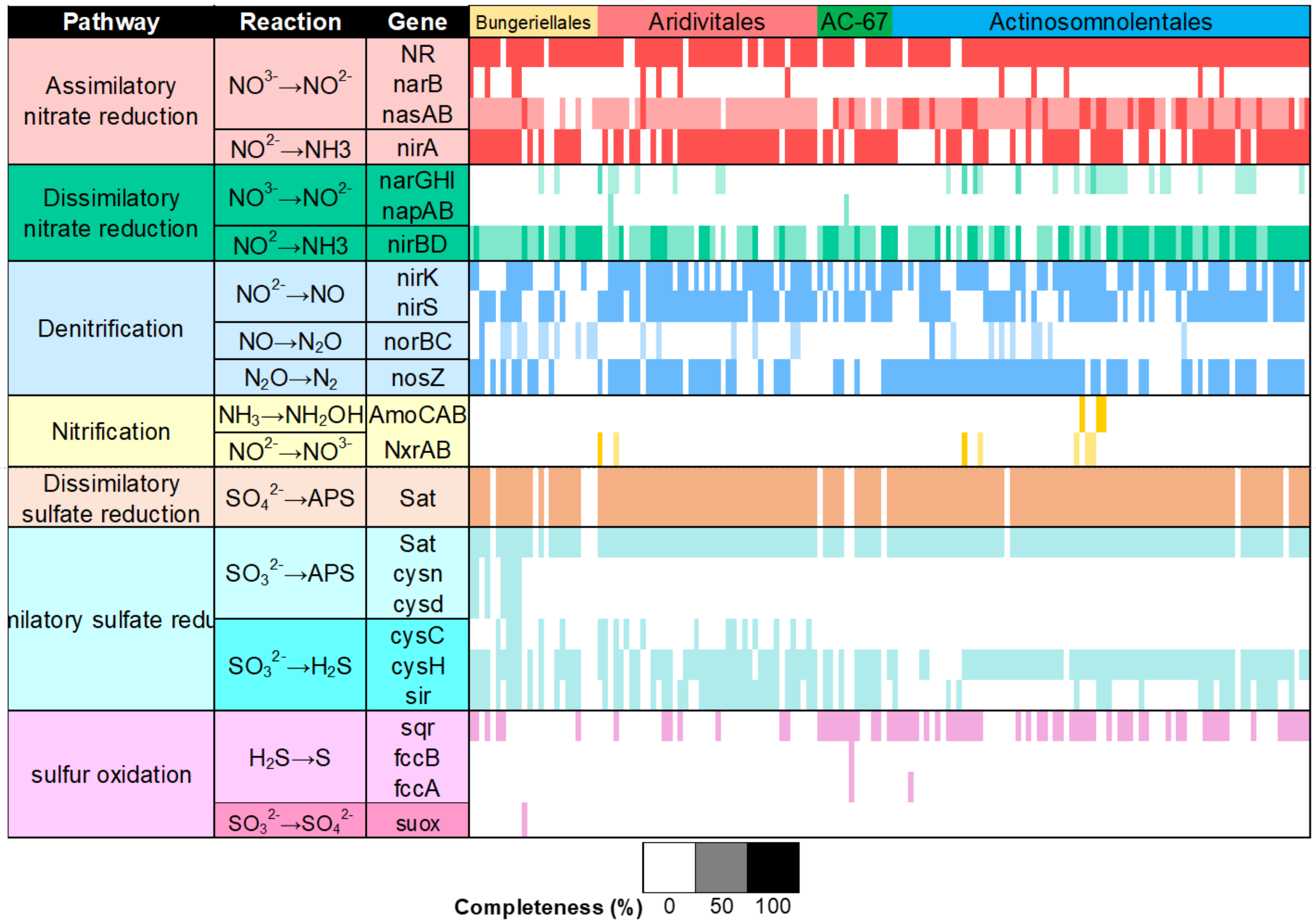

Supplementary Fig 10. Metabolic potential of nitrogen and sulfur cycling pathways in Aridivitia. Nitrogen metabolic pathways include assimilatory nitrate reduction, dissimilatory nitrate reduction, denitrification, and nitrification. Sulfur metabolic pathways include dissimilatory sulfate reduction, assimilatory sulfate reduction, and sulfur oxidation. The heatmap shows the distribution and completeness of key genes or proteins involved in these pathways across different genomes.

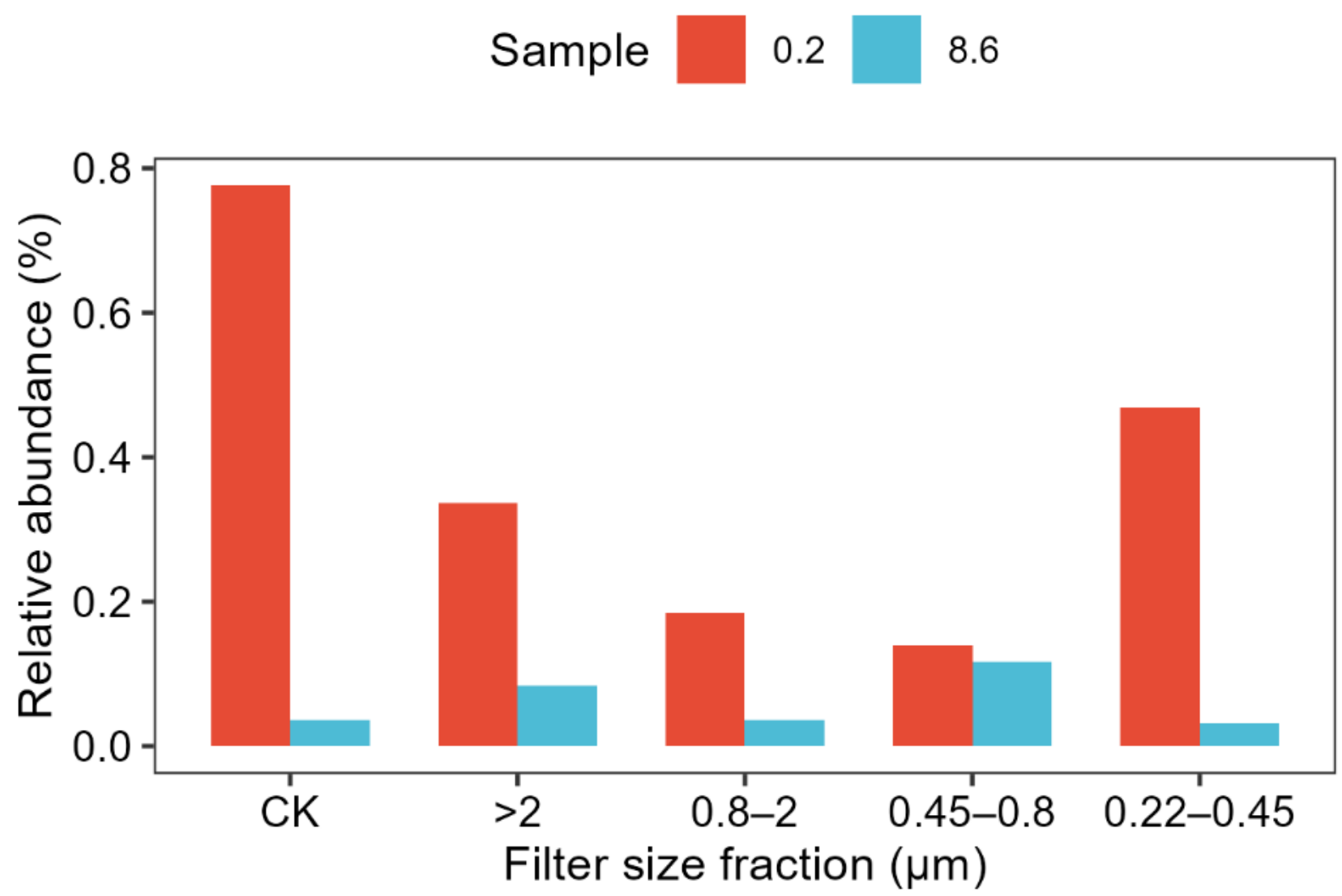

Supplementary Fig 11. Distribution of Aridivitia in tandem filtration experiments. Cell size distribution of Aridivitia was assessed via sequential filtration of soil microbial suspensions through membranes with decreasing pore sizes (2, 0.8, 0.45, and 0.22 μm). Samples were obtained from two depths (0.2 m and 8.6 m) at the Yangling sampling site. The bar plot illustrates the relative abundance of Aridivitia across different filtration fractions. "CK" represents the unfiltered original soil control. Source data are provided as a Source data file.

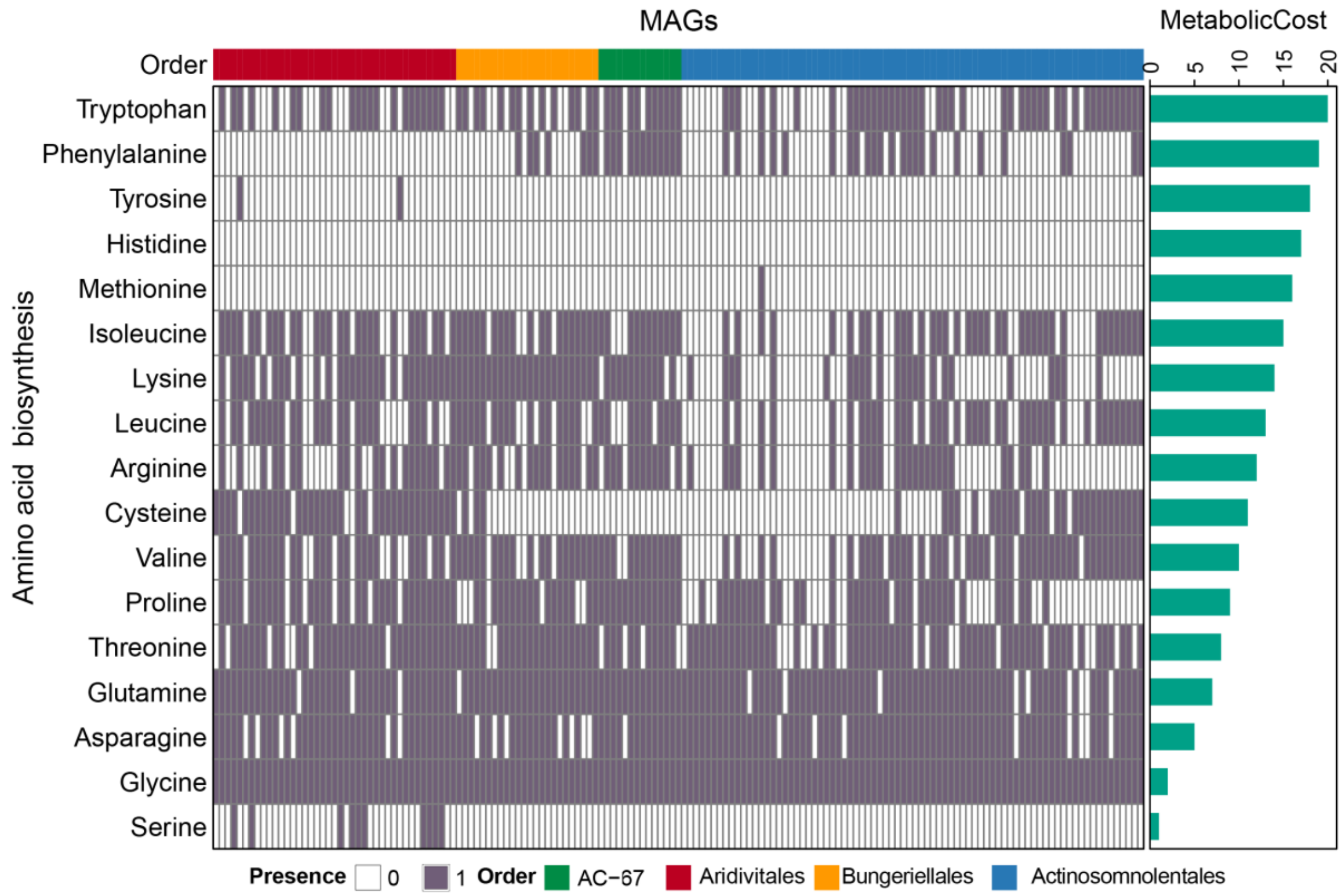

Supplementary Fig 12. Potential for essential amino acid biosynthesis across genomes within Aridivitia. The heatmap illustrates the completeness of biosynthetic pathways for 17 essential amino acids (1 for presence, 0 for absence) across MAGs from four orders (AC-67, Aridivitales, Bungeriellales, and Actinosomnolentales) within Aridivitia. The bar plot on the right indicates the average metabolic cost associated with the synthesis of each amino acid.

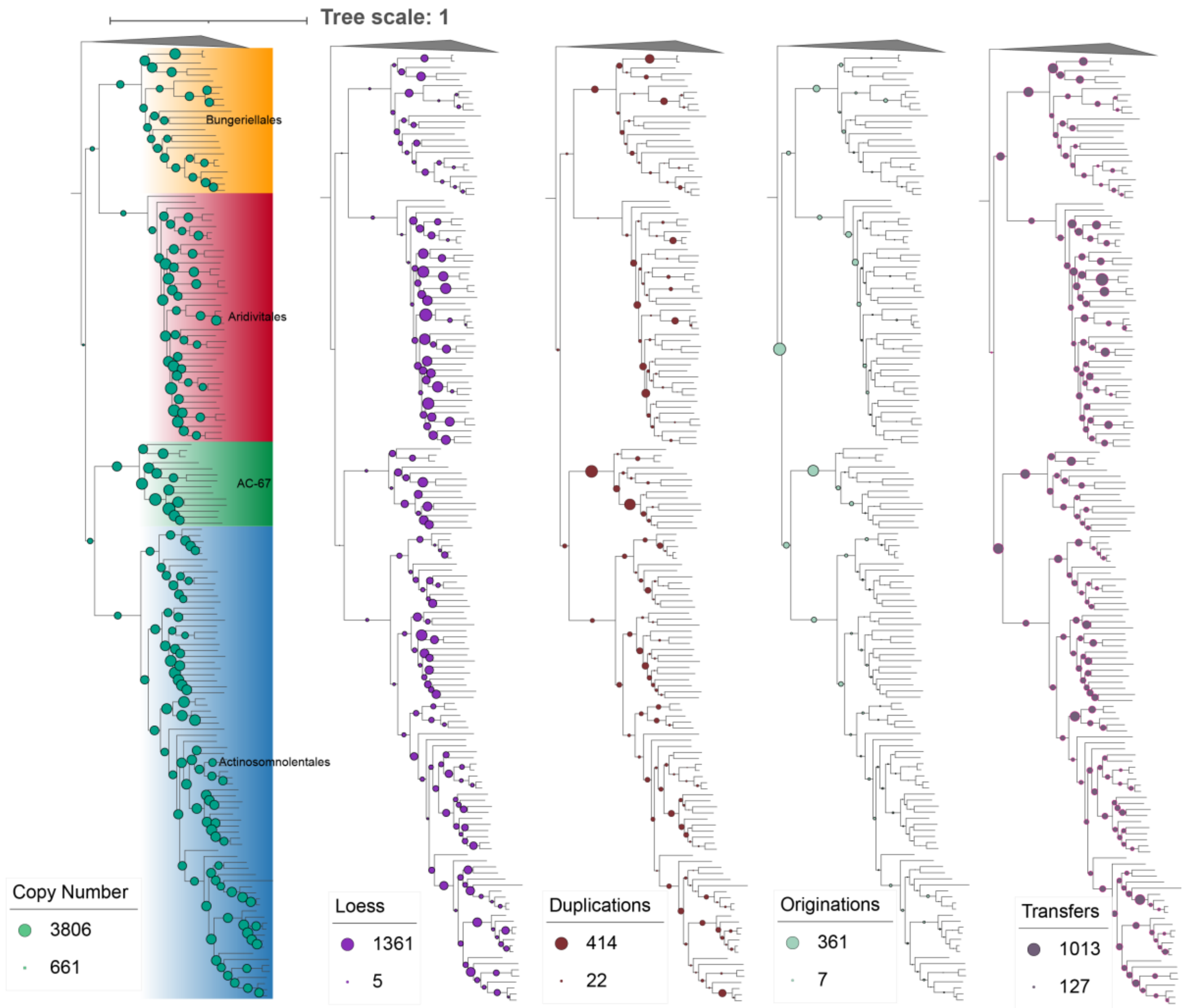

Supplementary Fig 13. Predicted genomic evolutionary events and copy numbers at the ancestral nodes within Aridivitia. The size of the circles on the phylogenetic tree nodes represents either the number of inferred evolutionary events (loss, duplication, origination, horizontal gene transfer) or gene copy numbers, depending on the panel.

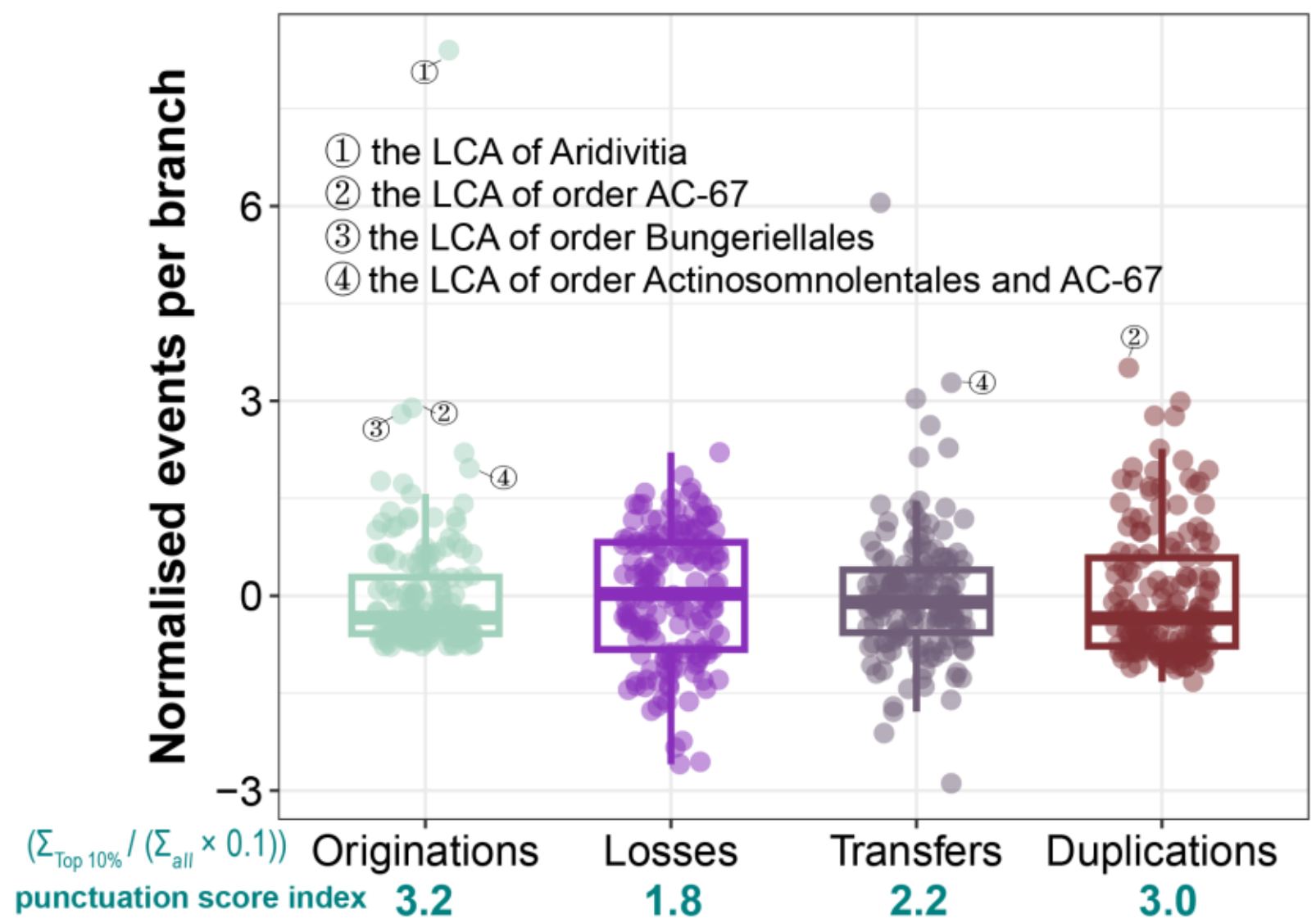

Supplementary Fig 14. Distribution of mechanisms of gene content change across the evolution of Aridivitia. Boxplots represent normalized events per branch [(events per branch − μ) / σ] for each evolutionary mechanism (n = 156 branches). Box plots show the median, 25th and 75th percentiles, with whiskers extending to the most extreme values within 1.5 × the interquartile range. Punctuation scores (bottom) represent the sum of events in the 10% of branches with the highest event numbers divided by 10% of the total events across all branches (Σ events in top 10% / (Σ events in all branches × 0.1)). Gene family origination exhibited the highest punctuation score (3.2), indicating that origination events were highly concentrated in a few branches, specifically along the stem lineages from the LCA of Aridivitia to the ancestors of the four orders. These concentrated origination events largely reflect horizontal gene transfer from lineages outside the group. Source data are provided as a Source data file.

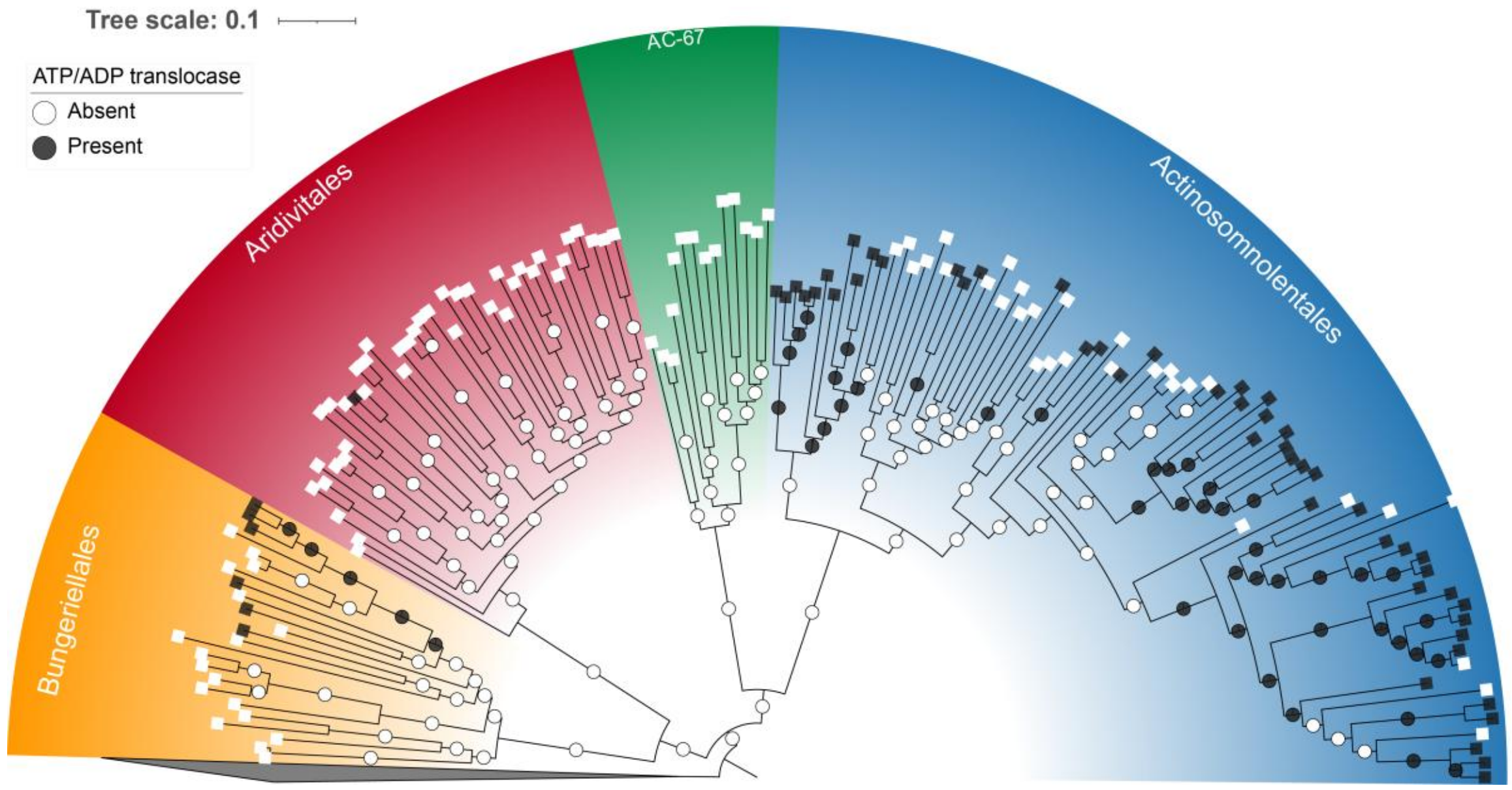

Supplementary Fig 15. Evolutionary history of ATP/ADP translocase in Aridivitia. The tree shows the presence (filled circles) or absence (open circles) of ATP/ADP translocase at ancestral nodes (circles) and extant members (squares). Background colors represent different orders.

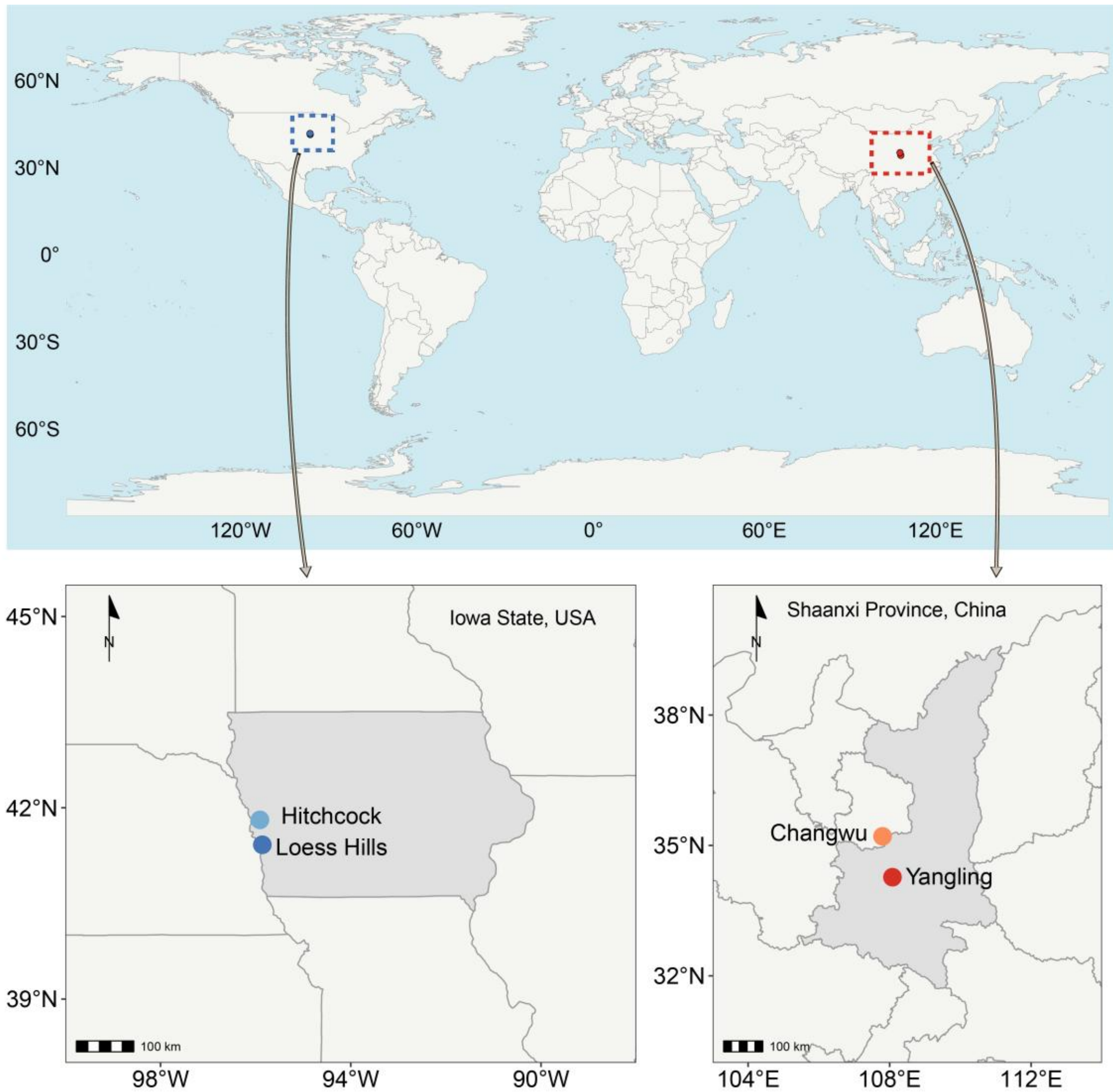

Supplementary Fig 16. Geographic locations of the soil profiles in this study. Sampling sites are located in Shaanxi Province, China, including Yangling and Changwu, and in Iowa, USA, including Hitchcock Nature Center and Loess Hills State Forest. This figure was generated using R, ggplot2, sf, rnaturalearth, maps, and ggspatial.

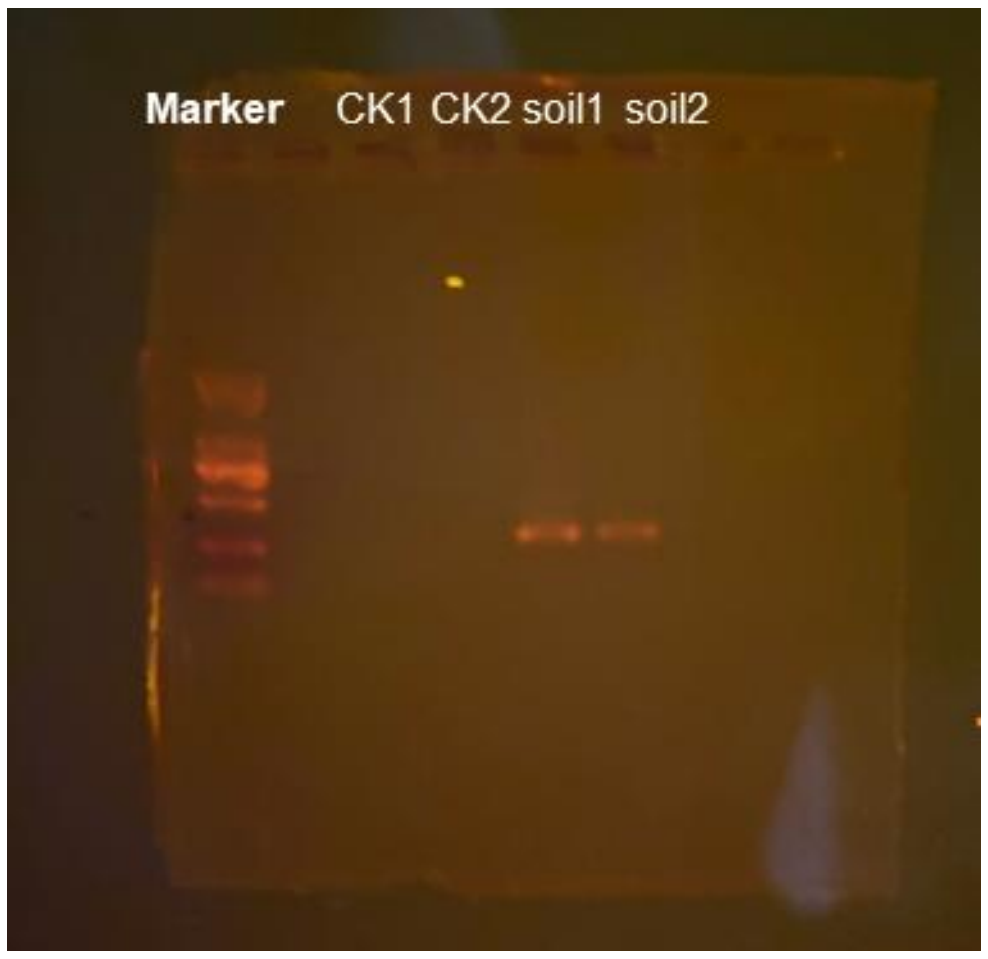

Supplementary Fig 17. Agarose gel electrophoresis of PCR products from blank extraction controls and soil samples. PCR amplification was performed using primers targeting the bacterial 16S rRNA gene V4 region for blank extraction controls and representative soil DNA samples. No visible amplification products were detected in the blank controls, whereas clear amplification bands were observed in the soil samples.

Supporting Information References

1. Thompson, L. R. *et al.* A communal catalogue reveals Earth's multiscale microbial diversity. *Nature* **551**, 457–463 (2017).
2. Woodcroft, B. J. *et al.* Comprehensive taxonomic identification of microbial species in metagenomic data using SingleM and Sandpiper. *Nat. Biotechnol.*, 1–6 (2025).
3. Labouyrie, M. *et al.* Patterns in soil microbial diversity across Europe. *Nat. Commun.* **14**, 3311 (2023).
4. Bissett, A. *et al.* Introducing BASE: the Biomes of Australian Soil Environments soil microbial diversity database. *GigaScience* **5**, 21 (2016).
5. Feng, W. *et al.* Diversification, niche adaptation, and evolution of a candidate phylum thriving in the deep Critical Zone. *Proc. Natl Acad. Sci. USA* **122**, e2424463122 (2025).
