# Supplementary material for: Genome-reduced Aridivitia evolved predation to thrive in Earth’s oligotrophic depths
Source: Nat Commun. 2026 Oct 14;17:11624. doi: 10.1038/s41467-026-78342-x (PMC13755416; doi:10.1038/s41467-026-78342-x)
Supplement: Supplementary file 2 [file 41467_2026_78342_MOESM2_ESM.pdf]

Description of Additional Supplementary Files

File Name: Supplementary Data 1
Description: Genomic information of the 958 Aridivitia MAGs.

File Name: Supplementary Data 2
Description: MetaSupplementary Data for the eight independent soil profile studies.

File Name: Supplementary Data 3
Description: Genomic information of the non-redundant representative Aridivitia MAGs.

File Name: Supplementary Data 4
Description: Pairwise amino acid identity (AAI) matrix of the Aridivitia MAGs.

File Name: Supplementary Data 5
Description: Pairwise average nucleotide identity (ANI) matrix of the Aridivitia MAGs.

File Name: Supplementary Data 6
Description: Summary of genomic features of the Aridivitia group.

File Name: Supplementary Data 7
Description: Completeness of KEGG metabolic modules in Aridivitia MAGs.

File Name: Supplementary Data 8
Description: Annotation and classification of carbohydrate-active enzymes (CAZymes) in Aridivitia MAGs.

File Name: Supplementary Data 9
Description: Predicted peptidases in Aridivitia MAGs (MEROPS Supplementary Database).

File Name: Supplementary Data 10
Description: Detailed KEGG Orthology (KO) annotations for Aridivitia MAGs.

File Name: Supplementary Data 11
Description: Classification and distribution of hydrogenases in Aridivitia MAGs.

File Name: Supplementary Data 12
Description: Genes associated with a potential predatory lifestyle in Aridivitia MAGs.

File Name: Supplementary Data 13
Description: Predicted secreted peptidases in Aridivitia MAGs.

File Name: Supplementary Data 14
Description: Quantification of genomic evolutionary events and proteome changes during the evolution of the Aridivitia group.

File Name: Supplementary Data 15
Description: Reconstructed metabolic states of key early ancestral nodes within the Aridivitia group.

File Name: Supplementary Data 16
Description: Relative abundance of Aridivitia MAGs across soil profile samples based on read recruitment.
