# Supplementary material for: Genome-reduced Aridivitia evolved predation to thrive in Earth’s oligotrophic depths
Source: Nat Commun. 2026 Oct 14;17:11624. doi: 10.1038/s41467-026-78342-x (PMC13755416; doi:10.1038/s41467-026-78342-x)
Supplement: Supplementary file 3 [file 41467_2026_78342_MOESM4_ESM.pdf]

nature portfolio

Corresponding author(s): Xuejun Yan; Xun Qian

Last updated by author(s): Aug 15, 2026

Reporting Summary

Nature Portfolio wishes to improve the reproducibility of the work that we publish. This form provides structure for consistency and transparency in reporting. For further information on Nature Portfolio policies, see our Editorial Policies and the Editorial Policy Checklist.

Statistics

For all statistical analyses, confirm that the following items are present in the figure legend, table legend, main text, or Methods section.

n/a	Confirmed	
☐	☒	The exact sample size (*n*) for each experimental group/condition, given as a discrete number and unit of measurement
☐	☒	A statement on whether measurements were taken from distinct samples or whether the same sample was measured repeatedly
☐	☒	The statistical test(s) used AND whether they are one- or two-sided<br>*Only common tests should be described solely by name; describe more complex techniques in the Methods section.*
☒	☐	A description of all covariates tested
☒	☐	A description of any assumptions or corrections, such as tests of normality and adjustment for multiple comparisons
☐	☒	A full description of the statistical parameters including central tendency (e.g. means) or other basic estimates (e.g. regression coefficient) AND variation (e.g. standard deviation) or associated estimates of uncertainty (e.g. confidence intervals)
☒	☐	For null hypothesis testing, the test statistic (e.g. *F*, *t*, *r*) with confidence intervals, effect sizes, degrees of freedom and *P* value noted<br>*Give P values as exact values whenever suitable.*
☒	☐	For Bayesian analysis, information on the choice of priors and Markov chain Monte Carlo settings
☒	☐	For hierarchical and complex designs, identification of the appropriate level for tests and full reporting of outcomes
☒	☐	Estimates of effect sizes (e.g. Cohen's *d*, Pearson's *r*), indicating how they were calculated

Our web collection on statistics for biologists contains articles on many of the points above.

Software and code

Policy information about availability of computer code

Data collection	No software was used for data collection. Soil samples were obtained through field sampling. Amplicon and metagenomic sequencing were performed on the Illumina NovaSeq platforms, and metatranscriptomic sequencing was conducted on the Illumina HiSeq platform.
Data analysis	Data analysis was performed using established bioinformatics tools, including QIIME2 v2023.7; Trimmomatic v0.39; MetaSPAdes v3.13.0; MetaWRAP v1.3.0; CheckM2 v1.0.2; GTDB-Tk v2.4.0; CoverM v0.7.0; Ribodetector v0.3.2; Bowtie2 v2.2.5; featureCounts v2.1.1; Kingfisher v0.4.1; iRep v1.10; dRep v3.2.2; Barrnap v0.9; tRNAscan-SE v2.0.12; IQ-TREE v2.3.3; CompareM v0.1.2; Prokka v1.14.6; Prodigal v2.6.3; KofamScan v1.3.0; METABOLIC v4.0; SignalP v6.0; MAFFT v7.525/v7.49; TrimAl v1.4; OrthoFinder v2.5.5; FastANI; gRodon; DIAMOND; HMMER; dbCAN_sub; TXSScan; GapMind; HydDB; iTOL; ALEobserve; ALEml_undated. No custom code central to the study was used.

For manuscripts utilizing custom algorithms or software that are central to the research but not yet described in published literature, software must be made available to editors and reviewers. We strongly encourage code deposition in a community repository (e.g. GitHub). See the Nature Portfolio guidelines for submitting code & software for further information.

Data

Policy information about availability of data

All manuscripts must include a data availability statement. This statement should provide the following information, where applicable:

- Accession codes, unique identifiers, or web links for publicly available datasets
- A description of any restrictions on data availability
- For clinical datasets or third party data, please ensure that the statement adheres to our policy

The raw sequencing data generated in this study have been deposited in the NCBI Sequence Read Archive (SRA) under the BioProject accession number PRJNA1417468. The MAGs affiliated with Aridivitia generated and analyzed during this study are publicly available on Figshare with the identifier https://doi.org/10.6084/m9.figshare.31046038. All other data supporting the findings of this study, including supplementary tables, figures, and other supporting information, are included in the published article and/or its supplementary materials. Source data are provided with this paper.The metagenome-assembled genomes (MAGs) generated and analyzed in this study are publicly available on Figshare at https://doi.org/10.6084/m9.figshare.31046038. All other data supporting the findings of this study are included in the Article and its Supplementary Information.

Research involving human participants, their data, or biological material

Policy information about studies with human participants or human data. See also policy information about sex, gender (identity/presentation), and sexual orientation and race, ethnicity and racism.

Reporting on sex and gender	n/a
Reporting on race, ethnicity, or other socially relevant groupings	n/a
Population characteristics	n/a
Recruitment	n/a
Ethics oversight	n/a

Note that full information on the approval of the study protocol must also be provided in the manuscript.

Field-specific reporting

Please select the one below that is the best fit for your research. If you are not sure, read the appropriate sections before making your selection.

☐ Life sciences ☐ Behavioural & social sciences ☒ Ecological, evolutionary & environmental sciences

For a reference copy of the document with all sections, see nature.com/documents/nr-reporting-summary-flat.pdf

Ecological, evolutionary & environmental sciences study design

All studies must disclose on these points even when the disclosure is negative.

Study description	This study investigated the distribution, activity, metabolic potential, and evolutionary history of Aridivitia in oligotrophic deep soils. Soil samples were collected from ten independent depth-resolved profiles across China and the United States, from which Aridivitia MAGs were recovered and analyzed together with publicly available datasets for comprehensive analyses.
Research sample	Soil samples were collected from ten soil profiles across China and the United States, generating 67 soil metagenomes, 2 metatranscriptomes, and 124 amplicon datasets. Publicly available soil amplicon datasets and MAGs from published studies and databases, including GTDB and JGI, were also integrated. These datasets were selected to expand environmental coverage and improve the robustness of large-scale comparative analyses.
Sampling strategy	In China, profiles were sampled at 0.2-m intervals, including a 20-m profile at Yangling and three 3-m profiles at Changwu. In Iowa, three profiles extending to 22 m were collected at each site at 1.22-m intervals. No statistical method was used to predetermine sample size; the sampling design was intended to capture variation across independent soil profiles and depths.
Data collection	Field sampling in the USA was conducted by Xun Qian on 25 August 2020, and sampling and metadata recording in China were conducted by Xiaonan Wan on 27 June 2022. Dataset curation and analysis were performed by Xiaonan Wan. Amplicon and metagenomic sequencing were performed on Illumina NovaSeq platforms, and metatranscriptomic sequencing was performed on an Illumina HiSeq platform. Publicly available datasets were retrieved from databases and reanalyzed.
Timing and spatial scale	Field sampling was conducted in two campaigns: soil samples from the USA were collected on 25 August 2020, and samples from China were collected on 27 June 2022. Samples were collected from depth-resolved soil profiles in China and the USA. Publicly available datasets were collected up to December 2024.

Data exclusions	No data were excluded from the analyses except for those failing standard quality control procedures.
Reproducibility	Field sampling included 10 independent soil profiles: one at Yangling, three at Changwu, three at Hitchcock Nature Center, and three at Loess Hills State Forest. For each sampled depth, a single soil sample was used for DNA or RNA extraction, generating one sequencing data for that depth.
Randomization	Randomization was not applicable, as samples were collected based on field locations and depth profiles rather than experimental group allocation.
Blinding	Blinding was not applicable to this study, as data collection and analysis were based on environmental samples and standardized computational procedures.

Did the study involve field work? ☒ Yes ☐ No

Field work, collection and transport

Field conditions	Environmental characteristics relevant to the study included soil depth, geological context, and soil physicochemical properties, including organic carbon, nitrogen, pH, and nutrient availability. These variables are described in the manuscript and Supplementary Information.
Location	Soil samples were collected from Yangling, China (34.27°N, 108.08°E), Changwu, China (35.21°N, 107.80°E), Hitchcock Nature Center, USA (41.42°N, 95.86°W), and Loess Hills State Forest, USA (41.81°N, 95.91°W).
Access & import/export	All samples were collected in accordance with local regulations and with appropriate site access permissions.
Disturbance	Soil sampling caused minimal disturbance to the environment and was limited to small-scale collection at each site.

Reporting for specific materials, systems and methods

We require information from authors about some types of materials, experimental systems and methods used in many studies. Here, indicate whether each material, system or method listed is relevant to your study. If you are not sure if a list item applies to your research, read the appropriate section before selecting a response.

Materials & experimental systems

n/a	Involved in the study
☒	☐ Antibodies
☒	☐ Eukaryotic cell lines
☒	☐ Palaeontology and archaeology
☒	☐ Animals and other organisms
☒	☐ Clinical data
☒	☐ Dual use research of concern
☒	☐ Plants

Methods

n/a	Involved in the study
☒	☐ ChIP-seq
☒	☐ Flow cytometry
☒	☐ MRI-based neuroimaging

Plants

Seed stocks	n/a
Novel plant genotypes	n/a
Authentication	n/a
