# Supplementary material for: Genome-reduced Aridivitia evolved predation to thrive in Earth’s oligotrophic depths
Source: Nat Commun. 2026 Oct 14;17:11624. doi: 10.1038/s41467-026-78342-x (PMC13755416; doi:10.1038/s41467-026-78342-x)
Supplement: Supplementary file 4 [file 41467_2026_78342_MOESM5_ESM.pdf]

nature portfolio

Peer Review File

Genome-reduced Aridivitia evolved predation to thrive in Earth’s oligotrophic depths

Corresponding Author: Professor Xun Qian

Parts of this Peer Review File have been redacted as indicated to remove third-party material.

This file contains all reviewer reports in order by version, followed by all author rebuttals in order by version.

Version 0:

Reviewer comments:

Reviewer #1

(Remarks to the Author)
The authors of “The evolution of predation underpins the ecological success of Subterricolia in Earth’s oligotrophic depths“ provide new insights to a so far understudied class of microorganisms. They used global sequencing datasets as well as samples from their own deep soil sampling campaign to reconstruct genomes and predict lifestyle as well as evolutionary changes. I found the study very interesting and easy to read in general, but also identified some weaknesses:
1) I found it very difficult to see how exactly the different data sets align, are they from the same depth, or just topsoils. Do the nutrient data complement the sequencing data sets or are they from different campaigns. A better overview about the specificities of the global datasets and the new created datasets would help.
2) The authors have a strong experimental emphasize on depths profiles, but they do not really pick up on depth related differences but just talk about deep soil. I would be interested if the properties they describe hold true for Subterricolia from all depth or if there is a gradual change in ecology. Also the depth analysed are now very deep in all cases, 30cm for example can still be very nutrient rich, depending on the plant coverage and management.
3) Methods and data quality need to be reported in much more detail e.g. sequencing output, read loss, library preparation, primers used etc. Particularly, I missed the mentioning of proper controls for DNA extraction and sequencing. Especially for low biomass samples this is mandatory practice and I urge the authors to include a proof of data quality regarding cross-contamination during PCR and extraction. If this is too much for the main text, I recommend to provide an extended method supplement.
4) I miss the ecological context of the findings, especially in the discussion mostly results are repeated without giving much context. Can this be transfered to oither oligotrophic habitats, what does it mean for soil development or nutrient cycling etc.

Generally, check the formatting of taxa names e.g. using italics.
Title: I feel the title is too specifically targeting the predation only
L75: Not a lot of sites. How representative can that be?
L79: compared to what?
L94: I am not sure if you can conclude that soils are the preferred habitat as long as similar amounts of samples from the other habitats were tested. I imagine simply much more soil samples were taken all over the globe.
Fig. 1b) I am not sure to what relative abundance refers to (also in Fig. S1), the proportion of reads in a sample or the proportion of samples in which the taxa were detected.
L100/Fig. 2a: How did you calculate the correlation? Based on the sample distribution I don’t think linear relationships apply and therefore simply linear correlations are not suitable to analyse the effect of environmental factors as they are mostly driven by the end-members.
L105ff: I think it is not surprising that nutrients are less in deep soils, but to a similar extent does the microbial biomass decline as well, thus competition is lower. I think it might make sense to at least consider that or calculate C/mirobail biomass.
L111: I wonder how the samples were normalized and how rare taxa were treated in the analysis, especially in such samples they might represent a large proportion of data, because of background noise in the PCR and the low biomass nature. It is therefore, critical whether those reads were removed or not.
L117: Are they really more active or is this a results of the shift in relative abundance. In other terms, are that absolute or

relative values.
L126: Define high quality.
L127: marker genes I assume.
L136: Is the genome size comparable to other oligotrophic taxa?y
L141: Compared to what? Other order of the class
L191: Please write taxa in italics.
Fig. 3b: Please add x-axis labels.
L303: Large parts of the discussion are very descriptive. I miss the ecological consequences as well as comparison to other groups from deep soils or other oligotrophic habitats e.g. deserts.
L336: Regarding the potential prey, I wonder if they might predate on themselves, like sacrifiying part of the community to enable survival of the rest.
L356: How many in total and per profile? How did you store and transport the samples, I assume they are very sensitive to changes especially for RNA analysis. How did you prevent contamination of deep soil with top soil microbes?
L358: Based on the description I am counting 7 sites not 10, please explain better how the sites are distributed. Moreover, I wonder how comparable the sampling intervals are and how you account for that later e.g. using volume related normalization.
L364: the method description is not sufficient it lacks the used primers, any implementation of extraction and PCR controls, which are mandatory for low biomass samples in particular.
L364: How did you do the libraries?
L367: That is outdated and not recommended to use anymore.
L371: Exactly mention what medium means.
L375: Did you control for leftover DNA and did you do cDNA synthesis? How did you do the libraries?
L381: normalized to mRNA reads only or to all reads including rRNA as well?
L391: Provide reference for SINGLEM.
L386: can you provide a map which shows the origin of your samples and how they are distributed globally?
L394 and L401: I do not understand how those datasets align with the EMP and Sandpiper datasets. I think it only makes sense if they are overlapping, or not?
L418: Do the 248 MAGs originate from your 10 sites or also the EMP, Sandsniper etc. It is generally difficult to follow your data sources and overview would be helpful, which data/datatypes come from which project/location.
L421: Did you find rRNA on each MAG? It is quite difficult in many cases.
L446: Not sure what you mean with ribosomal protein genes, as rRNA is not coding for proteins.
L454: You used different quality thresholds for every methods, e.g. here completeness above 90%. What was the rationale for that, it is quite difficult to compare the different analyses and I wonder if it is not better to go along one route consequently.
L476: From which samples? Your own 10 sites?

Reviewer #2

(Remarks to the Author)
This manuscript presents a genome-resolved metagenomic analysis of soil Actinobacteria, contributing valuable genomic data to a group previously known primarily through rRNA sequences and limited Metagenome-Assembled Genomes (MAGs). The most compelling finding is the relative abundance of the Subterricolales clade in oligotrophic soils and their streamlined genomes, which suggest a potentially parasitic lifestyle.
While the study is informative, several methodological limitations and missing ecological contexts should be addressed to strengthen the conclusions. Specifically, the limitations of MAG-based assembly regarding population microdiversity and the lack of direct visual evidence (microscopy) need to be better acknowledged.
General Observations:
A fundamental challenge with classical MAGs is their inherent incompleteness, particularly when microdiversity within a population interferes with the assembly process. The authors should explicitly discuss this limitation. To improve the robustness of the results, I suggest using individual MAGs for recruitment analysis at different depths. For instance, a population-level analysis of the 10 most abundant MAGs using parameters such as ANIr would provide much-needed depth.
The presence/absence of genes (as shown in Figure 3) is difficult to interpret definitively given the potential incompleteness of the MAGs. Conclusions drawn from these patterns should be framed with more caution.
The study would benefit significantly from a comparison with oligotrophic aquatic environments. For example, deep oligotrophic lakes are unusually rich in CPR bacteria—relatives of Actinobacteria that also exhibit streamlined genomes and parasitic-like features.
The lack of microscopy to confirm the suggested parasitic lifestyle is a notable disadvantage. While size fractionation is used, it is a limited substitute for direct observation of host-parasite interactions.
Specific comments by line:
Ln 46: Please provide a formal definition of "Earth’s Critical Zone."
Ln 79: The term "hypothesis" feels too strong here; "assumptions" may be more accurate.
Ln 85–95: This section would be more appropriate in the Introduction rather than the Results/Discussion.
Ln 106: "1.6-4.7 times" may not be perceived as a substantial difference by all readers; please provide further context for this comparison.
Ln 108: Briefly clarify how "relative abundance" was calculated. Specifically, it should be clear what the denominator

represents (e.g., % of total reads, % of total community).
Ln 110: Please address the inherent bias of amplicon technology. Because of the amplification step, it cannot be considered strictly quantitative.
Ln 134: High GC content is generally associated with large, "wasteful" genomes, whereas aquatic Actinobacteria (which are oligotrophic and streamlined) typically exhibit low GC content. The authors should comment on how their findings align with or diverge from this trend.
Ln 142: It is worth noting that this phenomenon mirrors observations in deep ocean waters.
Ln 187 onwards: Is there a documented relationship between Subterricolia and CPR bacteria? They share similar gene profiles; a brief discussion on potential relatedness is warranted.
Ln 207 onwards: The analogy to CPR bacteria is quite clear here, yet it seems to be introduced for the first time late in the manuscript. A comparative genomics analysis between these two groups, including the identification of the closest relative to Subterricolales within the CPR, would significantly strengthen the paper.

Version 1:

Reviewer comments:

Reviewer #1

(Remarks to the Author)
I am generally happy with the revisions made and the additional information provided regarding sampling, controls etc, as well as the improved discussion. I have only very few final comments.

Note S3 is not referenced in the text, please add at appropriate place.
L88: Still this global angle comes out of the blue, as it is also not so well introduced in the methods. Note S3 gives a nice summary of it, but it needs to be referenced in the main text. It might for example fit at the end of the introduction. Currently there you only explain your explerimental approach, but it would help to outline the different steps starting from bioinformatic over the specific new samplings etc. Alternatively, you might implement a short version of Note 3 at the beginning of the methods section.
L411: Also add the purpose of using both greengenes versions in the main text, maybe in brackets.

Reviewer #2

(Remarks to the Author)

Open Access This Peer Review File is licensed under a Creative Commons Attribution 4.0 International License, which permits use, sharing, adaptation, distribution and reproduction in any medium or format, as long as you give appropriate credit to the original author(s) and the source, provide a link to the Creative Commons license, and indicate if changes were made.
In cases where reviewers are anonymous, credit should be given to 'Anonymous Referee' and the source.
The images or other third party material in this Peer Review File are included in the article's Creative Commons license, unless indicated otherwise in a credit line to the material. If material is not included in the article's Creative Commons license and your intended use is not permitted by statutory regulation or exceeds the permitted use, you will need to obtain permission directly from the copyright holder.
To view a copy of this license, visit https://creativecommons.org/licenses/by/4.0/

Responses to Reviewers' Comments

Reviewer #1

The authors of "The evolution of predation underpins the ecological success of Subterricolia in Earth's oligotrophic depths" provide new insights to a so far understudied class of microorganisms. They used global sequencing datasets as well as samples from their own deep soil sampling campaign to reconstruct genomes and predict lifestyle as well as evolutionary changes. I found the study very interesting and easy to read in general, but also identified some weaknesses:

Comment 1: I found it very difficult to see how exactly the different data sets align, are they from the same depth, or just topsoils. Do the nutrient data complement the sequencing data sets or are they from different campaigns. A better overview about the specificities of the global datasets and the new created datasets would help.

Response 1: We sincerely thank the reviewer for the positive feedback and valuable suggestions. To improve the clarity of the relationships among different datasets, we have added Supplementary Note S3, which provides a systematic overview of all datasets used in this study, including their sources, sample types, sampling depths, and analytical purposes. We hope this additional information will help readers better understand how each dataset contributes to different aspects of this study. Briefly, the datasets integrated in this study were used to address different questions:

(1) First, to determine whether Subterricolia represents a geographically restricted lineage or a globally distributed microbial group, we assessed its global distribution patterns using two global-scale resources. These global datasets revealed that Subterricolia is widely distributed worldwide and is primarily associated with soil habitats.

- **The Earth Microbiome Project (EMP)** is an international open-science microbiome initiative aimed at characterizing microbial diversity across the Earth at a global scale. It integrates 27,751 16S rRNA gene samples from 97 studies spanning seven continents, 43 countries, and 17 environmental categories.
- **Sandpiper** is an online microbial community analysis platform developed by Queensland University of Technology, Australia, which provides microbial community composition profiles derived from 248,559 publicly available metagenomic datasets covering 217 habitat types, including marine, lake, freshwater, soil, hot spring, and wetland environments.

(2) After identifying its preference for soils, we further investigated the environmental factors associated with Subterricolia distribution using two large-scale soil 16S survey datasets. Both datasets provide 16S rRNA gene data together with comprehensive soil physicochemical measurements, enabling analyses of the associations between environmental factors and Subterricolia abundance. These analyses revealed that Subterricolia is significantly associated with soils containing lower organic carbon contents.

- **European soil dataset** organized by the Joint Research Centre (JRC) of the European Commission, which covers 715 sampling sites across 24 European countries, with sampling depths 0–20 cm.
- **The Biomes of Australian Soil Environments (BASE) dataset**, a nationwide soil microbiome survey across Australia comprising more than 1,400 samples collected from over 900 locations at depths of 0–30 cm.

(3) Consistent with its preference for low-organic-carbon soils, we further observed that Subterricolia is enriched in deeper, carbon-poor soil horizons, and that this pattern is widespread.

- **Soil profiles sampled in this study:** includes 16S, metagenome and soil organic carbon measurements from ten soil profiles collected in China and the United States. Analysis of the vertical distribution of Subterricolia showed that its relative abundance is consistently higher in low-organic-carbon deep soils compared to the organic-carbon-rich surface layers.
- **Independent validation:** To test whether this deep enrichment phenomenon is generalizable, we downloaded and reanalyzed 16S data from eight independent soil profile studies, covering different regions and research projects (see Supplementary Table 2). The results consistently showed that Subterricolia is also more abundant in the deep layers of these independent soil profiles.

(4) To understand how Subterricolia successfully colonizes and maintains predominance in oligotrophic soil such as deep soils, we comprehensively analyzed **the 248 MAGs recovered in this study together with publicly available genomes** of this lineage. These genomic resources allowed us to investigate the metabolic potential, ecological adaptation strategies, and evolutionary history of Subterricolia.

Comment 2: The authors have a strong experimental emphasize on depths profiles, but they do not really pick up on depth related differences but just talk about deep soil. I would be interested if the properties they describe hold true for Subterricolia from all depth or if there is a gradual change in ecology. Also the depth analysed are now very deep in all cases, 30cm for example can still be very nutrient rich, depending on the plant coverage and management.

Response 2: We thank the reviewer for these important and constructive suggestions.

We agree that a systematic analysis of depth-related changes in the entire soil microbiome would be an interesting and valuable research direction. However, **the primary focus of this study is not to describe the overall changes in soil microbial communities with depth**, but rather to use depth-resolved soil profiles to identify a bacterial lineage that successfully thrives in deep subsurface soils and to investigate the genomic and evolutionary strategies that may support its survival in low-resource environments.

Regarding whether these properties apply to Subterricolia across all depths, we would like to clarify that the features described here, including genome reduction, metabolic flexibility, and predatory capabilities, were derived from our comprehensive analysis of Subterricolia MAGs. **These features should be understood as the overall adaptive strategies of Subterricolia for persistence in low-resource environments.** As they represent the genomic potential of this lineage, we do not consider them to be restricted to a particular depth.

Regarding the reviewer's comment that a depth of 30 cm does not necessarily represent an oligotrophic environment, we would like to clarify that the 30-cm boundary mentioned in the Introduction is a conceptual distinction commonly used to describe surface and deep soils, rather than a definition of oligotrophic conditions. More importantly, the enrichment of Subterricolia in deep soils observed in this study is not based on this depth classification, but on the consistent pattern observed across multiple soil profiles, where Subterricolia was enriched in deeper layers with relatively limited resources compared with surface soils receiving higher resource inputs. For example, in the US soil profiles sampled in this study, Subterricolia reached up to 20% relative abundance even at a depth of 10 m, where soil organic carbon content was only approximately 0.2%.

Comment 3: Methods and data quality need to be reported in much more detail e.g. sequencing output, read loss, library preparation, primers used etc. Particularly, I missed the mentioning of proper controls for DNA extraction and sequencing. Especially for low biomass samples this is mandatory practice and I urge the authors to include a proof of data quality regarding cross-contamination during PCR and extraction. If this is too much for the main text, I recommend to provide an extended method supplement.

Response 3: We thank the reviewer for this important suggestion regarding methodological detail and data quality control.

(1) We have expanded the Materials and Methods accordingly by adding detailed information on primer sequences, library preparation, sequencing platforms, sequencing output, read retention statistics, and metatranscriptomic data processing. The revised sentences are presented below:

L398–L409: "The extracted DNA was subsequently sent to Biomarker Technologies (Beijing, China) for library preparation and sequencing. For amplicon sequencing, the V4 region of the 16S rRNA gene was amplified using primers 515F (5′-GTGYCAGCMGCCGCGGTAA-3′) and 806R (5′-GGACTACNVGGGTWTCTAAT-3′) for samples from China, whereas the V3–V4 region was amplified using primers 338F (5′-ACTCCTACGGGAGGCAGCA-3′) and 806R (5′-GGACTACHVGGGTWTCTAAT-3′) for samples from Iowa, USA. The amplified products were purified using magnetic beads, followed by PCR-based indexing to add sequencing adapters and sample-specific index sequences before sequencing on the Illumina NovaSeq 6000 platform (2×250 bp). For metagenomic sequencing, libraries were constructed using the VAHTS Universal Plus DNA Library Prep Kit for Illumina (Vazyme, ND617-02) and sequenced on the Illumina NovaSeq X Plus platform (2×150 bp)."

L411–L414: "For metagenomic analysis, sequencing generated an average of approximately 17 Gb of raw data per sample across 67 metagenomic libraries. Raw reads were quality-filtered using Trimmomatic v0.39[63], with an average of 96% of paired-end reads retained."

L425–L434: "The extracted RNA was subsequently sent to Majorbio (Shanghai, China), where rRNA was removed using the Ribo-Zero rRNA Removal Kit. The remaining RNA was fragmented and reverse-transcribed to generate double-stranded cDNA, followed by library construction using the TruSeq™ RNA Sample Prep Kit. Metatranscriptomic (2×150 bp) sequencing was performed on the Illumina HiSeq platform, generating approximately 11.3–11.6 Gb of raw data per sample across two metatranscriptomic libraries. Raw reads were quality-filtered and adapter-trimmed using Trimmomatic v0.39[63], retaining approximately 90% of paired-end reads. Ribosomal RNA (rRNA) reads were subsequently removed using Ribodetector v0.3.2[68], resulting in non-rRNA reads that accounted for approximately 60.0–66.9% of the raw reads."

(2) During DNA extraction of the deep-soil samples, blank extraction controls were included following our established practice to assess potential contamination. We have added a brief statement in the Materials and Methods section (L397): "… with blank extraction controls included to assess potential contamination (Supplementary Note S2; Supplementary Fig. 17)." We have also included Supplementary Note S2 to describe the setup and results of these blank extraction controls in detail. Briefly, the results are summarized as follows:

a, Qubit dsDNA High Sensitivity assay showed that all blanks were below the detection limit (< 0.005 ng/µL).

b, PCR amplification of the 16S rRNA gene followed by agarose gel electrophoresis revealed no visible bands in any of the blank controls, whereas representative deep-soil DNA samples yielded clear amplification products (Supplementary Fig. S17).

c, Quantitative PCR further confirmed the absence of bacterial 16S rRNA gene amplification signals in all blank controls (Cq = NA).

d, The blank controls were subjected to the same library preparation and sequencing workflow as the soil samples; library concentrations of all blanks were below the minimum requirement for library construction, and therefore no sequencing data were generated.

Collectively, these results indicate that contamination introduced during DNA extraction and PCR amplification was negligible.

Comment 4: I miss the ecological context of the findings, especially in the discussion mostly results are repeated without giving much context. Can this be transfered to oither oligotrophic habitats, what does it mean for soil development or nutrient cycling etc.

Response 4: We sincerely appreciate the reviewer's valuable feedback. We have revised the Discussion section accordingly, as outlined below.

(1) In the Discussion, we have removed descriptive language while retaining the key findings and their interconnections, comparisons with previous studies, and ecological implications.

(2) We have added comparisons between the metabolic strategies of Subterricolia and those of other oligotrophic lineages and habitats, to highlight the broader relevance of our findings for other nutrient-limited environments.

a. We have added a comparison between Subterricolia and oligotrophic marine and lacustrine microorganisms as well as CPR lineages. We point out their convergent adaptations in genome reduction, increased coding density, and ultra-small cell size, as well as the differences in GC content and proteomic C/N ratios between Subterricolia and these aquatic oligotrophs, and discuss the ecological mechanisms underlying these differences (L305–L315).

L305–L315: The reduced genomes, increased coding density, and ultra-small cell sizes lower non-essential metabolic costs and energy requirements during cell division and growth, resembling those observed in nutrient-poor epipelagic marine and lacustrine microorganisms as well as Candidate Phyla Radiation (CPR) lineages[40-44], suggesting convergent adaptation to energy-limited environments. However, unlike these aquatic oligotrophs that tend to have low GC content and increased proteomic C/N ratios, Subterricolales exhibits high GC content and reduced proteomic C/N ratios alongside genome reduction. This difference reflects contrasting nutrient constraints, with aquatic systems generally being nitrogen-limited whereas soil microbial growth is predominantly carbon-limited[45]. Because GC base pairs have lower C/N ratios, selection for higher GC content and reduced proteomic C/N ratios can reduce cellular carbon demand[42,45.]

b. We noted that the flexible carbon and energy metabolism observed in Subterricolia resembles metabolic features that similarly support the persistence of dominant microorganisms in other oligotrophic ecosystems, including glacier forelands, Antarctic desert soils, and permafrost (L331–L334).

L331–L334: Similar metabolic features also provide important support for the persistence of dominant microorganisms in other oligotrophic ecosystems, such as glacier forelands, Antarctic deserts, and permafrost[11,46,47].

c. In the predation section, we have also noted that the strategy of acquiring nutrients by lysing prey cells when resources are scarce is common in oligotrophic marine, groundwater, lake, and soil environments (L350–L353).

L350–L353: Although bacterial predation is often considered energy-intensive, this strategy is common in oligotrophic marine, groundwater and lake systems as well as soils, where the lysis of prey cells can provide predators with access to nutrients and energy that are otherwise scarce[53-55].

(3) We have strengthened the ecological discussion to clarify the broader implications of our findings.

a. We now indicate that through the degradation of complex organic substrates, Subterricolia may contribute to the slow transformation and turnover of buried organic carbon in deep soils, thereby influencing the long-term dynamics of organic carbon across soil profiles (L322–L325).

L322–L325: This metabolic capacity suggests that Subterricolia may contribute to the slow transformation and turnover of buried complex organic carbon including microbial necromass in deep soils, thereby influencing the long-term dynamics of organic carbon across soil profiles.

b. We have added a brief description and detailed presentation of nitrogen and sulfur cycling pathways in Subterricolia in the main text (L191–L193) and supplementary materials (Supplementary Note S1; Supplementary Fig. 10). We also summarize that the combination of genome reduction, predatory lifestyle, metabolic flexibility, and diverse metabolic potential for carbon, nitrogen, and sulfur cycling collectively suggests that Subterricolia likely plays important roles in subsurface energy and nutrient cycling, as well as in regulating microbial community structure (L371–L374).

L191–L193: Subterricolia members also broadly encode diverse pathways involved in nitrogen and sulfur cycling (Supplementary Fig. 10; Supplementary Note S1;).

L371–L374: Combined with its diverse metabolic potential for carbon, nitrogen, and sulfur, these features also suggest that Subterricolia may play important roles in subsurface energy and nutrient cycling, as well as in controlling microbial populations.

Comment 5: Generally, check the formatting of taxa names e.g. using italics.

Response 5: We thank the reviewer for this suggestion. We have carefully checked and corrected the formatting of taxonomic and gene names in the revised manuscript, ensuring that genus names, species names, and gene names are italicized where appropriate and consistently formatted throughout the manuscript.

Comment 6: Title: I feel the title is too specifically targeting the predation only

Response 6: We thank the reviewer for this suggestion. We have revised the title from "The evolution of predation underpins the ecological success of Subterricolia in Earth's oligotrophic depths" to "Genome-reduced Subterricolia evolved predation to thrive in Earth's oligotrophic depths"

Comment 7: L75: Not a lot of sites. How representative can that be?

Response 7: We appreciate the reviewer's concern about the number of sampling sites. As noted in our response to Comment 1, **the primary focus of this study is to identify a bacterial lineage that is enriched in deep subsurface and similar nutrient-poor soils through continuous depth profiling, and to characterize the genomic and evolutionary strategies that underpin its success in such oligotrophic environments.** The ten profiles collected in this study provide complete and continuous vertical sampling (down to 22 m), from which 16S data, metagenomic,

metatranscriptomic, and soil physicochemical data were generated. **These rich datasets robustly support the enrichment of Subterricolia in deeper soils.** This pattern was further validated by 16S data from eight independent soil profile studies, covering different geographic regions and research projects (Supplementary Table 2). Moreover, the 248 Subterricolia MAGs recovered from the metagenomic data of these profiles provided valuable genomic resources for dissecting its metabolic potential, ecological adaptation strategies, and evolutionary history.

Comment 8: L79: Compared to what?

Response 8: Our original statement that “Subterricolia possesses unique strategies for nutrient and energy acquisition” was intended to describe strategies that differ from those of many microorganisms commonly found in soils, particularly taxa whose growth is constrained in oligotrophic deep soils. Given the predominance of Subterricolia in such nutrient-limited environments, we hypothesized that its success in these habitats may be supported by specialized strategies for nutrient and energy acquisition. To avoid ambiguity, we have revised the wording from “unique strategies” to “specialized strategies” to more accurately convey our meaning.

Comment 9: L94: I am not sure if you can conclude that soils are the preferred habitat as long as similar amounts of samples from the other habitats were tested. I imagine simply much more soil samples were taken all over the globe.

Response 9: We thank the reviewer for the comment. As noted in our response to Comment 1, the two global datasets we used, the Earth Microbiome Project (EMP) and Sandpiper, represent large-scale public resources with extensive habitat coverage in current microbial ecology research and have been widely used to investigate habitat distributions and preferences of microbial lineages. For example, Omnitrophota are enriched in both aquatic and soil environments, Woesearchaeota show the highest relative abundance in salt marshes, Bathyarchaeia are enriched in hot springs and hydrothermal sediments, whereas Venenivibrio is mainly restricted to geothermal springs in New Zealand. These cases demonstrate that habitat preferences can be successfully detected using these datasets, and that not all lineages preferentially occur in soils.

In the EMP dataset (n = 23,828), soil samples (n = 4,279) were not the most abundant category; freshwater (n = 4,915) and host-associated samples (n = 4,158) were comparable or even more numerous. Similarly, in the Sandpiper metagenomic dataset (n = 248,559), host-associated environments (particularly the human gut) were dominant, while marine (n = 10,563), sediment (n = 3,674), and freshwater (n = 2,887) all had sample sizes comparable to soils (n = 8,945).

For Subterricolia, both global 16S and metagenomic surveys consistently show that it is most frequently detected and exhibits the highest relative abundance in soils. We have revised the wording in the main text from “indicating” to “suggesting” (L98): “suggesting that soils are the primary habitat of Subterricolia.”

Comment 10: Fig. 1b) I am not sure to what relative abundance refers to (also in Fig. S1), the proportion of reads in a sample or the proportion of samples in which the taxa were detected.

Response 10: Relative abundance refers to the proportion of reads assigned to Subterricolia relative to the total reads within each sample, rather than the proportion of samples in which this lineage was detected. We revised the relevant sentence in the main text as follows:

L92-L94: "Among these Subterricolia-positive samples, 62% were from soils, which together contributed 83% of the total Subterricolia reads across the EMP dataset (Fig. 1b)."

We have added to the Figure 1c legend (L841): "…… relative abundance, calculated as the proportion of reads assigned to Subterricolia within each sample, ……"

The Supplementary Fig. 1 legend was revised to state: "Relative abundance was calculated as the proportion of reads assigned to Subterricolia within each sample."

Comment 11: L100/Fig. 2a: How did you calculate the correlation? Based on the sample distribution I don't think linear relationships apply and therefore simply linear correlations are not suitable to analyse the effect of environmental factors as they are mostly driven by the end-members.

Response 11: We used Spearman's rank correlation to evaluate the relationships between Subterricolia relative abundance and environmental variables. Spearman's correlation is a non-parametric method that does not assume linearity; instead, it assesses overall monotonic trends based on ranked values and is suitable for non-normally distributed ecological data. Because Spearman's correlation is based on ranks rather than raw values, it is less sensitive to the magnitude of extreme values than correlations calculated directly from raw values.We therefore interpreted these correlations cautiously as general associations between Subterricolia abundance and soil properties.

Comment 12: L105ff: I think it is not surprising that nutrients are less in deep soils, but to a similar extent does the microbial biomass decline as well, thus competition is lower. I think it might make sense to at least consider that or calculate C/mirobail biomass.

Response 12: We sincerely thank the reviewer for raising this insightful point. We fully agree that microbial biomass generally decreases as SOC declines with soil depth. In fact, as reported by Sun et al. (2020), microbial biomass decreases more rapidly than SOC. Therefore, mathematically, the C/microbial biomass ratio does increase in deeper soils.

However, we would caution against interpreting this higher mathematical ratio as evidence that individual microorganisms in deeper soils have access to more usable carbon or experience weaker resource limitation. As we pointed out in the introduction (L50–L52), **organic carbon in deep soils is not only low in quantity but also exists in highly stable, recalcitrant forms that decompose and turn over very slowly, making it largely inaccessible to microbes**. Spohn et al. (2016) directly measured microbial C uptake rates using ^{18}O labeling and found that deep-soil microbes take up carbon about 100 times slower than surface microbes. Fierer et al. (2003) also showed that physiological stress indicators in microbes increase with depth, indicating that carbon limitation actually becomes more severe in deeper layers.

We have also taken the reviewer's suggestion and revised the wording throughout the manuscript to avoid interpreting our observations as evidence of a competitive advantage. The specific changes are listed below:

L106: "suggesting competitive advantage in nutrient-poor, neutral-to-alkaline soils" has been revised to "suggesting that it is relatively more abundant in nutrient-poor, neutral-to-alkaline soils."

L234: "potentially conferring a competitive advantage…" has been revised to "potentially providing a key ecological advantage …"

L289: "…may confer a competitive advantage in oligotrophic soils" has been revised to "… may have contributed to the ecological success in oligotrophic soils."

L352: "where predators can gain a competitive advantage by efficiently lysing prey cells to acquire nutrients when these are scarce" has been revised to "where the lysis of prey cells can provide predators with access to nutrients and energy that are otherwise scarce"

References:

Sun T, Wang Y, Hui D, et al. Soil properties rather than climate and ecosystem type control the vertical variations of soil organic carbon, microbial carbon, and microbial quotient[J]. Soil Biology and Biochemistry, 2020, 148: 107905.

Spohn M, Klaus K, Wanek W, et al. Microbial carbon use efficiency and biomass turnover times depending on soil depth–Implications for carbon cycling[J]. Soil Biology and Biochemistry, 2016, 96: 74-81.

Fierer N, Schimel J P, Holden P A. Variations in microbial community composition through two soil depth profiles[J]. Soil Biology and Biochemistry, 2003, 35(1): 167-176.

Comment 13: L111: I wonder how the samples were normalized and how rare taxa were treated in the analysis, especially in such samples they might represent a large proportion of data, because of background noise in the PCR and the low biomass nature. It is therefore, critical whether those reads were removed or not.

Response 13: For the 16S data from the eight independent soil profile studies, we processed each study independently, including quality filtering, primer removal, sequence denoising, and feature table construction. Within each study, all samples were rarefied to the same sequencing depth before relative abundance was calculated. Because sequencing depth differed among studies, the rarefaction depth was determined separately for each study based on the sequencing depth distribution and rarefaction curves (Figure below). Specifically, the selected rarefaction depth was chosen to meet two criteria: (i) retaining most samples while excluding samples with very low sequencing depth; and (ii) ensuring that most samples approached the plateau phase of the rarefaction curves, thereby providing sufficient coverage of microbial diversity. Samples with sequencing depths below the corresponding rarefaction threshold were excluded. The rarefaction curves and selected rarefaction depths for each study are shown below.

We did not apply an arbitrary abundance threshold to remove rare ASVs. Rare ASVs represented a certain proportion of the total ASV count; however, because they collectively contributed only a very small number of reads, their impact on the relative abundance estimates of dominant taxa such as Subterricolia was negligible. In addition, in oligotrophic deep soils, some low-abundance ASVs may represent real but rare microbial taxa with potential ecological significance (Sogin et al., 2006). Removing them could lead to the loss of important biological signals. Therefore, after DADA2 denoising and rarefaction, we retained all rare ASVs.

Reference:

Sogin M L, Morrison H G, Huber J A, et al. Microbial diversity in the deep sea and the underexplored "rare biosphere"[J]. Proceedings of the National Academy of Sciences, 2006, 103(32): 12115-12120.

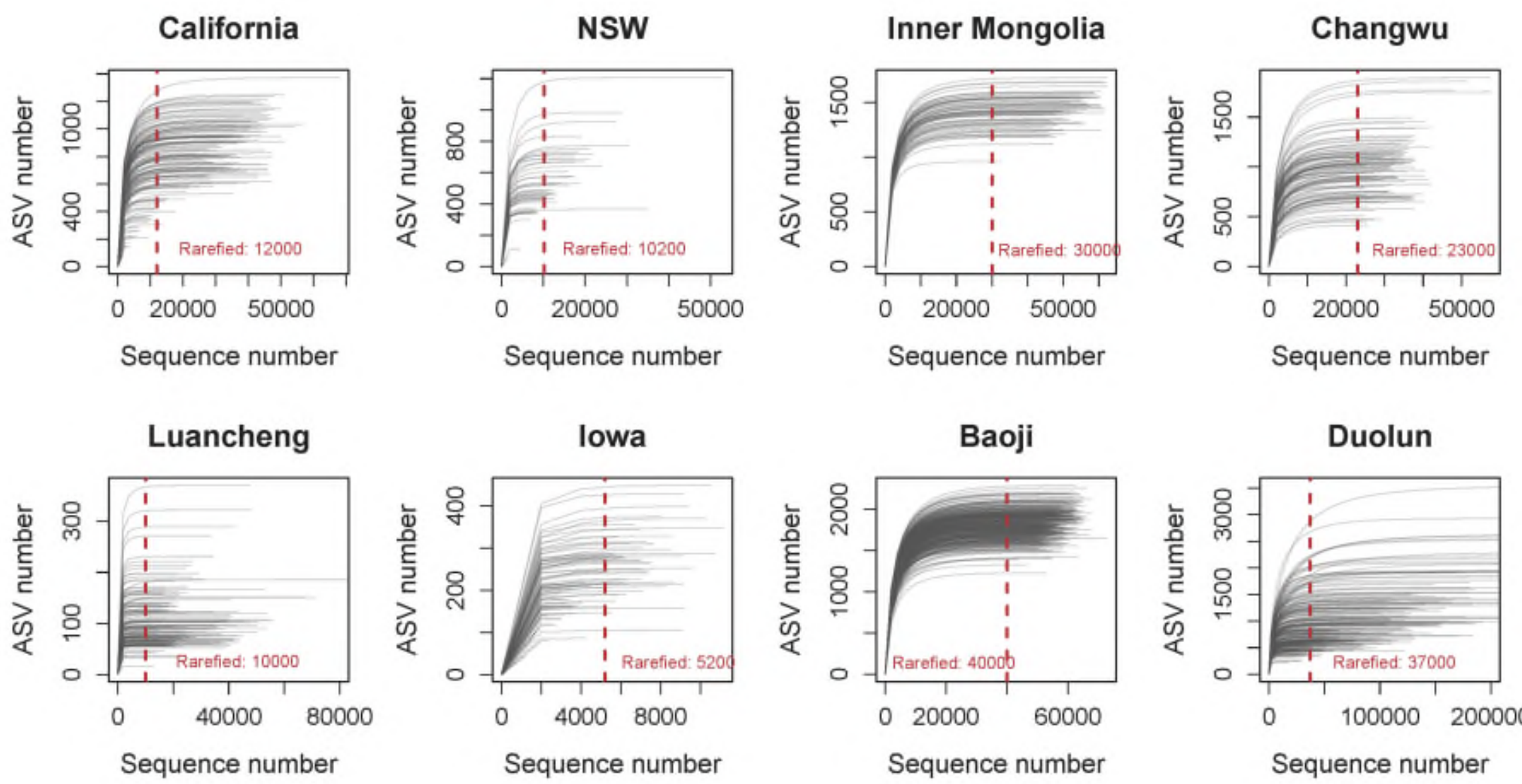

Response Figure 1. Rarefaction curves and selected rarefaction depths for each of the eight independent soil profile studies.

Comment 14: L117: Are they really more active or is this a results of the shift in relative abundance. In other terms, are that absolute or relative values.

Response 14: We thank the reviewer for raising this important point. The transcriptional activity shown here is a relative measure based on TPM (transcripts per million), reflecting the relative contribution of Subterricolia transcripts within each metatranscriptomic dataset rather than absolute transcriptional activity. To avoid ambiguity, we have revised the sentence (L122) by replacing "activity" with "relative transcriptional contribution". The revised sentence now reads:

“Metatranscriptomic analysis of the Yangling profile similarly placed Subterricolia among the top 10 most transcriptionally active lineages, with its median relative transcriptional contribution at 0.8 m being 2.3-fold higher than at 0.2 m (Fig. 2d).”

Comment 15: L126: Define high quality.

Response 15: We have now provided a definition of “high-quality” in the revised manuscript (L131). The sentence has been revised as follows:

“Species-level dereplication produced 423 non-redundant representatives (85 from this study), averaging 83% completeness and 2.1% contamination, with 52 meeting the MIMAG high-quality draft genome standard (>90% completeness, <5% contamination, the presence of 23S, 16S, and 5S rRNA genes, and at least 18 tRNAs).”

Comment 16: L127: marker genes I assume.

Response 16: Yes. We used the GTDB bac120 approach (Park et al., 2018), which identifies 120 conserved bacterial single-copy marker genes from each genome and infers phylogeny from the

concatenated alignment of their encoded protein sequences. We have revised “marker proteins” to “marker genes” in the manuscript (L134).

Reference

Parks D H, Chuvochina M, Waite D W, et al. A standardized bacterial taxonomy based on genome phylogeny substantially revises the tree of life[J]. Nature biotechnology, 2018, 36(10): 996-1004.

Comment 17: L136: Is the genome size comparable to other oligotrophic taxa?

Response 17: Yes. The average genome size of Subterricolia MAGs in our dataset (2.89 Mb) is comparable to that of other oligotrophic soil taxa with compact genomes. For example, Candidatus Udaeobacter copiosus, an abundant soil bacterium proposed to have undergone genome reduction, has an estimated genome size of ~2.81 Mb (Brewer et al., 2016). This value is similar to that of Subterricolia and both are substantially smaller than the predicted effective mean genome size of soil bacteria (~4.74 Mb).

In addition, as described in our response to Comment 4, we have expanded the Discussion to compare the genome reduction strategies of Subterricolia with those observed in other oligotrophic lineages, including nutrient-poor marine and lacustrine microorganisms and Candidate Phyla Radiation (CPR) lineages (L305–L315).

Reference

Brewer, T. E., Handley, K. M., Carini, P., Gilbert, J. A. & Fierer, N. Genome reduction in an abundant and ubiquitous soil bacterium ‘Candidatus Udaeobacter copiosus’. Nat. Microbiol. 2, 1–7 (2016).

Comment 18: L141: Compared to what? Other order of the class

Response 18: Yes, the comparison here was made among different orders within the class Subterricolia. Compared with the other orders within Subterricolia, namely JAHWKV01, CADDZG01, and AC-67, the dominant order Subterricolales has the smallest genomes, highest GC content, and highest coding density. We have added "Compared with other orders within Subterricolia" at the beginning of the sentence (L146) to clarify the comparison context.

Comment 19: L191: Please write taxa in italics.

Response 19: Thank you for pointing this out. We have italicized *Bdellovibrio bacteriovorus* in the revised manuscript (L199).

Comment 20: Fig. 3b: Please add x-axis labels.

Response 20: We appreciate the reviewer’s suggestion. Considering that adding repeated x-axis labels to each panel would make the figure more crowded, we have added a legend in Fig. 3b to clearly indicate the correspondence between colors and taxonomic groups.

Comment 21: L303: Large parts of the discussion are very descriptive. I miss the ecological consequences as well as comparison to other groups from deep soils or other oligotrophic habitats e.g. deserts.

Response 21: This concern has been addressed in our response to Comment 4. Briefly, we have substantially revised the Discussion section by reducing descriptive language, adding comparisons between Subterricolia and other oligotrophic lineages and ecosystems, and expanding the discussion of its potential ecological roles in carbon, nitrogen, and sulfur cycling.

Comment 22: L336: Regarding the potential prey, I wonder if they might predate on themselves, like sacrifiying part of the community to enable survival of the rest.

Response 22: We thank the reviewer for raising this interesting question.

Under nutrient-limited conditions, some bacteria can obtain nutrients through cannibalism. For example, in myxobacteria and Bacillus subtilis, programmed cell death of a subpopulation can release nutrients that support the survival of the remaining cells (Gonzalez-Pastor et al., 2003; Claessen et al., 2014). We therefore agree that a similar intra-population process cannot be fully excluded in Subterricolia. However, direct evidence supporting or refuting this possibility is currently lacking. Accordingly, we have strengthened the acknowledgment of limitations in the revised Discussion section. Despite multiple lines of evidence from genomic, transcriptomic, and cell-size data supporting a predatory lifestyle, direct microscopic observations of predator–prey interactions are currently lacking (L356–L359). We have also noted that future targeted cultivation combined with microscopic observations will help further resolve these unknowns (L378–L379).

L356–L359 "It should be noted that, in the absence of direct microscopic observations of predator–prey interactions, the predatory behavior of Subterricolia and the identity of its potential prey remain to be experimentally validated."

L378–L379 "Future targeted cultivation under both laboratory and in situ conditions, combined with direct microscopic observations, will help further validate these strategies, especially predation."

References:

González-Pastor J E, Hobbs E C, Losick R. Cannibalism by sporulating bacteria[J]. Science, 2003, 301(5632): 510-513.

Claessen D, Rozen D E, Kuipers O P, et al. Bacterial solutions to multicellularity: a tale of biofilms, filaments and fruiting bodies[J]. Nature Reviews Microbiology, 2014, 12(2): 115-124.

Comment 23: L356: How many in total and per profile? How did you store and transport the samples, I assume they are very sensitive to changes especially for RNA analysis. How did you prevent contamination of deep soil with top soil microbes?

Response 23:

Sample numbers:

Shaanxi, China

- Yangling: One 20-m soil profile was sampled at 0.2-m intervals, yielding 100 samples.
- Changwu: Three 3-m soil profiles were sampled at 0.2-m intervals. Each profile yielded 15 samples, resulting in 45 samples in total.

Iowa, USA

- Three soil profiles were collected at Hitchcock Nature Center and three at Loess Hills State Forest, giving a total of six profiles. For each profile, 18 long-core sections were collected at 1.22-m intervals to a final depth of approximately 22 m. In addition, two shallow-depth samples (0.2 m

and 0.8 m) were collected from an adjacent short core. Therefore, each Iowa profile contained 20 samples, resulting in 120 samples across the six profiles.

Thus, A total of 265 soil samples were collected from the ten soil profiles. When selecting samples for nucleic acid extraction and sequencing, we aimed to cover each meter of depth to ensure representative coverage of the continuous depth profiles (Fig. 2b; Supplementary Fig. 9).

To minimize cross-contamination among depths, separate soil cores were collected for each depth interval during drilling. For each recovered core section, only the interior portion of the core was retained for downstream analyses, whereas material from the top and bottom of the core was discarded. This is because the ends of the core are more susceptible to disturbance from drilling, contact with the coring device, and mixing with adjacent soil layers, whereas the interior portion of the core is unlikely to be affected by contamination and is therefore more representative of in situ soil at the target depth.

After collection, one portion of each sample was placed in an insulated container with ice packs and transported to the laboratory within 2 h for further processing, while another portion was immediately flash-frozen in liquid nitrogen for RNA-related analyses. Upon return to the laboratory, samples were stored at 4°C, −20°C, or −80°C depending on the requirements of subsequent analyses.

We thank the reviewer for the comment. In response, we have revised and expanded the Materials and Methods section to include additional details on the measures taken to minimize cross-contamination among depths, as well as the procedures used for sample transport and storage (L390–L393): "Each soil sample was divided into two portions: one was placed in an insulated container with ice packs and transported to the laboratory as soon as possible, while the other was immediately flash-frozen in liquid nitrogen on site for subsequent RNA extraction. Upon arrival at the laboratory, samples were stored at 4°C, −20°C, or −80°C."

Comment 24: L358: Based on the description I am counting 7 sites not 10, please explain better how the sites are distributed. Moreover, I wonder how comparable the sampling intervals are and how you account for that later e.g. using volume related normalization.

Response 24: We have revised the description of the sampling sites to more clearly indicate how the ten soil profiles were distributed (L383–L388) "Soil samples were obtained from a total of ten soil profiles across Shaanxi, China, and Iowa, USA. These included one 20-m profile from Yangling and three 3-m profiles from Changwu in Shaanxi, China, as well as three 22-m profiles from Hitchcock Nature Center and three 22-m profiles from Loess Hills State Forest in Iowa, USA. Soil profiles in China were sampled at 0.2-m intervals, whereas those in Iowa were sampled at 1.22-m intervals."

Regarding the reviewer's concern about sampling intervals, we acknowledge that the Chinese profiles (0.2 m) and the Iowa profiles (1.22 m) were sampled at different depth intervals. However, we would like to clarify that **this does not affect the main conclusions of our study**. Each sample represents a specific depth within a given soil profile, and our analysis focuses on changes in the relative abundance of Subterricolia across depths within individual profiles. Importantly, we do not directly compare samples from identical absolute depths across different profiles (e.g., 1 m in a Chinese profile versus 1.22 m in an Iowa profile). Instead, we focus on the distribution pattern of this lineage along the depth gradient within each profile. In other words, we examine whether Subterricolia becomes enriched with increasing depth relative to surface soils within the same profile. Therefore, differences in sampling interval may affect depth resolution but do not affect the

calculation of relative abundance within individual samples or the interpretation of vertical distribution patterns.

Comment 25: L364: the method description is not sufficient it lacks the used primers, any implementation of extraction and PCR controls, which are mandatory for low biomass samples in particular.

Response 25: This issue has been addressed in our response to Comment 3. We have added the primer sequences and library preparation details to the Materials and Methods section (L397–L409; L423–L432). In addition, we have stated in the main text (L397) that blank extraction controls were included during DNA extraction, and have included Supplementary Note S2 and Supplementary Fig. S17 to present the corresponding results. We believe these additions adequately address the reviewer's concerns.

Comment 26: L364: How did you do the libraries?

Response 26: This issue has been addressed in our response to Comment 3. Briefly, DNA samples were sent to Biomarker Technologies (Beijing, China) for library preparation and sequencing. For 16S sequencing, PCR products were purified and indexed before sequencing (L405). For metagenomic sequencing, libraries were constructed using the VAHTS Universal Plus DNA Library Prep Kit for Illumina (L408). For metatranscriptomic sequencing, extracted RNA was sent to Majorbio (Shanghai, China), where rRNA was removed, cDNA was synthesized by reverse transcription, and libraries were constructed using the TruSeq™ RNA Sample Prep Kit (L425).

Comment 27: L367: That is outdated and not recommended to use anymore.

Response 27: We thank the reviewer for this suggestion. As noted in the Introduction (L61), the focal lineage of this study corresponds to the 16S rRNA sequence name 0319-7L14, which is widely used in Greengenes 13.8 and has been adopted in many previous studies. Therefore, to maintain consistency with earlier work, we retained Greengenes 13.8 for taxonomic assignment. At the same time, we also performed taxonomic assignment using Greengenes 2 (where this lineage is designated as UBA4738), and the results were consistent between the two databases. We have revised the Methods section (L411) to indicate that both Greengenes 13.8 and Greengenes 2 were used.

Comment 28: L371: Exactly mention what medium means.

Response 28: We have added the definition of “medium quality” according to your comment (L418). The sentence now reads: “The quality of reconstructed MAGs was assessed using CheckM2 v1.0.25[6], and only genomes of medium quality or higher (⩾50% completeness and <10% contamination) were retained.”

Comment 29: L375: Did you control for leftover DNA and did you do cDNA synthesis? How did you do the libraries?

Response 29: The RNeasy PowerSoil Total RNA Kit (Qiagen) used in this study effectively separates RNA from DNA during the extraction process. Briefly, total nucleic acids (RNA and DNA) extracted from soil are co-bound to an anion-exchange gravity-flow column. RNA is subsequently

eluted using a specific salt solution, whereas DNA remains bound to the column. Therefore, no additional DNA removal steps were taken. **More importantly, the Agilent 5300 electropherograms of the RNA samples showed clear RNA peaks and no detectable DNA peak (see figure below), further confirming the absence of DNA contamination.**

The questions regarding cDNA synthesis and library construction have also been addressed in our response to Comment 3. Briefly, the extracted RNA was sent to Majorbio (Shanghai, China), where rRNA was removed, cDNA was synthesized by reverse transcription, and libraries were constructed using the TruSeq™ RNA Sample Prep Kit (L426).

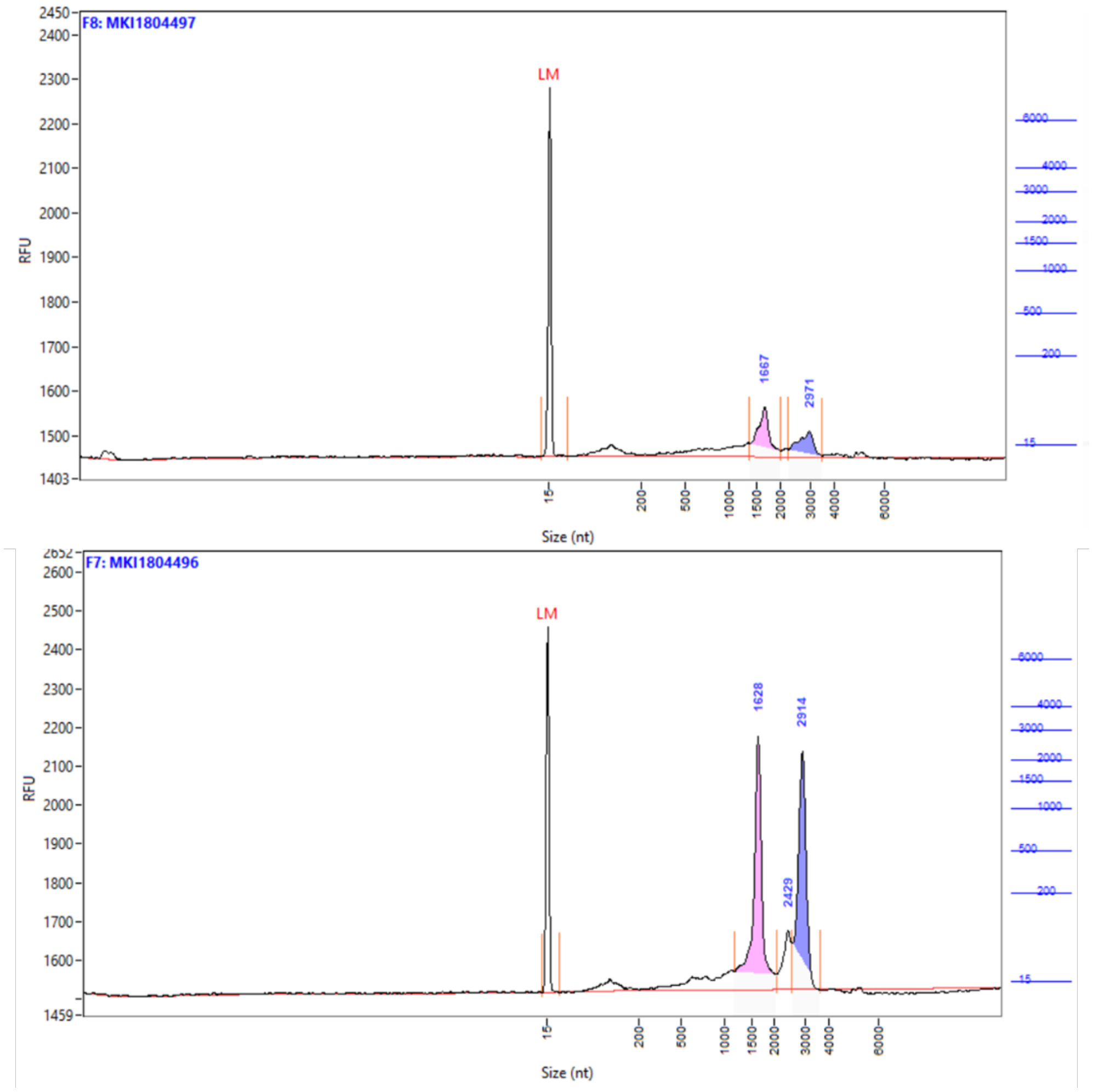

Response Figure 2. Agilent 5300 electropherograms of RNA samples. Both samples showed clear RNA peaks with no detectable DNA peaks, indicating high RNA purity and no DNA contamination.

Comment 30: L381: normalized to mRNA reads only or to all reads including rRNA as well?

Response 30: TPM values were calculated using only the non-rRNA reads. As described in the Methods (L432), rRNA reads were first removed using Ribodetector, and only the resulting non-rRNA reads were mapped to MAGs and used for gene expression quantification with FeatureCounts. Therefore, TPM normalization was based exclusively on non-rRNA reads and did not include rRNA reads. These non-rRNA reads predominantly represent mRNA transcripts.

Comment 31: L391: Provide reference for SINGLEM.

Response 31: We have now added a citation for SingleM. Both SingleM and Sandpiper are described in reference 17.

Comment 32: L386: can you provide a map which shows the origin of your samples and how they are distributed globally?

Response 32: Thank you for this suggestion. As L386 (now L441 in the revised manuscript) specifically refers to the EMP and Sandpiper datasets, the global geographic distribution of samples in which Subterricolia was detected is already shown in Figure 1a. In this figure, each point represents a sampling location where Subterricolia was detected, and point size reflects its relative abundance.

If the intention of this comment was to request the geographic locations of the soil profiles sampled in this study, we have additionally added Supplementary Fig. 16, showing the locations of the sampling sites in China and the United States. This figure provides readers with a clearer overview of the geographic origins of the samples analyzed in this study.

Comment 33: L394 and L401: I do not understand how those datasets align with the EMP and Sandpiper datasets. I think it only makes sense if they are overlapping, or not?

Response 33: This point has been clarified in our response to Comment 1 and in the newly added Supplementary Note S3. **These datasets were not intended to overlap at the sample level. Instead, they were used to address different but complementary questions.** EMP and Sandpiper were used to assess the global distribution of Subterricolia. The European and BASE soil datasets, which include both 16S data and soil physicochemical properties, were used to examine relationships between Subterricolia relative abundance and environmental factors. The soil profiles we sampled, together with additional soil profile data from eight independent studies, were used to evaluate its vertical distribution across soil profiles. We have clarified the overview and analytical purpose of each dataset in Supplementary Note S3.

Comment 34: L418: Do the 248 MAGs originate from your 10 sites or also the EMP, Sandsniper etc. It is generally difficult to follow your data sources and overview would be helpful, which data/datatypes come from which project/location.

Response 34: We thank the reviewer for this suggestion. The 248 Subterricolia MAGs were recovered from the metagenomic data generated from the ten soil profiles sampled in this study, and were not derived from EMP, Sandpiper, or other datasets. EMP and Sandpiper were used only to assess the global distribution and habitat association of Subterricolia. We have added Supplementary Note S3 to provide an overview of all datasets used in this study.

Comment 35: L421: Did you find rRNA on each MAG? It is quite difficult in many cases.

Response 35: No. Our intention was to indicate that all MAGs were screened for potential rRNA genes, not that every MAG successfully identified rRNA genes. We have revised the sentence (L477) for clarity: "Potential rRNA genes in these MAGs were identified using Barrnap v0.9 (https://github.com/tseemann/barrnap)"

Comment 36: L446: Not sure what you mean with ribosomal protein genes, as rRNA is not coding for proteins.

Response 36: Here, "ribosomal protein-coding genes" refers to genes encoding ribosomal proteins, rather than rRNA genes. Examples include rps genes encoding small-subunit ribosomal proteins, such as rpsL, rpsC, and rpsD, and rpl genes encoding large-subunit ribosomal proteins, such as rplB, rplC, and rplD. In gRodon, codon usage bias in these genes is used to predict maximal microbial growth rates. To avoid ambiguity, we have revised "ribosomal protein-coding genes" to "genes encoding ribosomal proteins" in the manuscript (L500).

Comment 37: L454: You used different quality thresholds for every methods, e.g. here completeness above 90%. What was the rationale for that, it is quite difficult to compare the different analyses and I wonder if it is not better to go along one route consequently.

Response 37: Different quality thresholds were applied because different analyses have different sensitivities to genome incompleteness.

For phylogenetic and taxonomic analyses, we included all MAGs with completeness ⩾ 50% and contamination ⩽ 10%. This threshold follows the MIMAG standard for medium-quality genomes (Bowers et al., 2017) and maximizes genomic representation.

For genomic feature analyses (e.g., genome size, GC content, and coding density), we adopted a more stringent threshold following previous studies (Seymour et al., 2023) to reduce biases introduced by missing genomic regions. In this study, we selected a threshold of completeness ⩾ 80% and contamination ⩽ 5% to balance data reliability and phylogenetic diversity.

For functional annotation and orthologous group analyses, we further restricted the dataset to high-quality genomes (⩾90% completeness and ⩽5% contamination). These analyses rely on accurate gene presence/absence calls and are sensitive to false inferences of gene absence caused by incomplete assemblies. Adopting stricter quality cutoffs for such analyses is a common practice (e.g., Sheridan et al., 2020, 2022, 2023; Seymour et al., 2023).

We would like to clarify that, **although the use of different quality thresholds resulted in partially different datasets for different analyses, these analyses address distinct research questions and were not intended for direct comparison.** For example, phylogenetic analyses aim to capture broad lineage diversity; genomic feature analyses aim to accurately estimate genome characteristics of this lineage; and orthologous group and functional annotation analyses aim to infer the presence and absence of functional traits both in extant genomes and throughout their evolutionary history. Therefore, each analysis employed the quality threshold most appropriate for its specific objective, and the use of different thresholds does not affect the validity of the conclusions drawn from individual analyses.

References:

Bowers R M, Kyrpides N C, Stepanauskas R, et al. Minimum information about a single amplified genome (MISAG) and a metagenome-assembled genome (MIMAG) of bacteria and archaea[J]. Nature biotechnology, 2017, 35(8): 725-731.

Seymour C O, Palmer M, Becraft E D, et al. Hyperactive nanobacteria with host-dependent traits pervade Omnitrophota[J]. Nature Microbiology, 2023, 8(4): 727-744.

Sheridan, P.O., Raguideau, S., Quince, C. et al. Gene duplication drives genome expansion in a major lineage of Thaumarchaeota. Nat Commun 11, 5494 (2020).

https://doi.org/10.1038/s41467-020-19132-x

Sheridan P O, Raguideau S, Quince C, et al. Gene duplication drives genome expansion in a major lineage of Thaumarchaeota[J]. Nature communications, 2020, 11(1): 5494.

Sheridan P O, Meng Y, Williams T A, et al. Recovery of Lutacidiplasmatales archaeal order genomes suggests convergent evolution in Thermoplasmatota[J]. Nature Communications, 2022, 13(1): 4110.

Baker B A, Gutiérrez-Preciado A, Rodriguez del Rio A, et al. Expanded phylogeny of extremely halophilic archaea shows multiple independent adaptations to hypersaline environments[J]. Nature Microbiology, 2024, 9(4): 964-975.

Comment 38: L476: From which samples? Your own 10 sites?

Response 38: We have revised the Methods section (L530) to clarify the samples used in the tandem filtration experiment: "…on two fresh soil samples collected from the Yangling profile (0.2 m and 8.6 m depth)…". These samples were collected from the Yangling profile in Shaanxi, China, representing surface and deep soils, respectively. DNA was extracted from the unfiltered control and each filtration fraction, followed by 16S rRNA gene amplicon sequencing to assess the distribution of Subterricolia across size fractions (Supplementary Fig. 11).

Responses to Reviewers' Comments

Reviewer #2

This manuscript presents a genome-resolved metagenomic analysis of soil Actinobacteria, contributing valuable genomic data to a group previously known primarily through rRNA sequences and limited Metagenome-Assembled Genomes (MAGs). The most compelling finding is the relative abundance of the Subterricolales clade in oligotrophic soils and their streamlined genomes, which suggest a potentially parasitic lifestyle.

Comment 1: While the study is informative, several methodological limitations and missing ecological contexts should be addressed to strengthen the conclusions. Specifically, the limitations of MAG-based assembly regarding population microdiversity and the lack of direct visual evidence (microscopy) need to be better acknowledged.

A fundamental challenge with classical MAGs is their inherent incompleteness, particularly when microdiversity within a population interferes with the assembly process. The authors should explicitly discuss this limitation. To improve the robustness of the results, I suggest using individual MAGs for recruitment analysis at different depths. For instance, a population-level analysis of the 10 most abundant MAGs using parameters such as ANIr would provide much-needed depth.

Response 1: We sincerely thank the reviewer for the positive feedback and valuable suggestions. In response, we have added a statement to the Discussion section acknowledging that genomic microdiversity can interfere with metagenomic assembly and affect the completeness of reconstructed MAGs: L375–L378: "Given that genomic microdiversity can complicate metagenomic assembly and result in incomplete genomic reconstructions, MAG-based metabolic inferences may not fully capture the functional potential of this lineage."

In addition, we have also acknowledged in the Discussion that, despite multiple lines of evidence from genomic, transcriptomic, and cell-size data, direct microscopic observations of predator–prey interactions are currently lacking:

L356–L359: "It should be noted that, in the absence of direct microscopic observations of predator–prey interactions, the predatory behavior of Subterricolia and the identity of its potential prey remain to be experimentally validated."

L378–L379: "Future targeted cultivation under both laboratory and in situ conditions, combined with direct microscopic observations, will help further validate these strategies, especially predation."

Regarding the reviewer's suggestion of cross-depth recruitment analyses, we calculated the recruitment rates of the 248 Subterricolia MAGs recovered in this study across all metagenomic samples using CoverM v0.7.0 and provided these results in Supplementary Table 16 (L421). These analyses show the representation of individual MAGs across samples from different soil depths.

Comment 2: The presence/absence of genes (as shown in Figure 3) is difficult to interpret definitively given the potential incompleteness of the MAGs. Conclusions drawn from these patterns should be framed with more caution.

Response 2: We thank the reviewer for highlighting this important issue. In response, we have revised the manuscript to ensure that all conclusions based on MAG-derived gene presence/absence patterns are expressed in a more cautious manner. The specific revisions are as follows:

(1) "…possess…" was changed to "…encode…" (L154; L168);

(2) "…possess…" was changed to "…was inferred to have possessed…" (L262);

(3) "…indicate…" was changed to "…suggest…" (L193; L272);

(4) "…conferring the ability…" was changed to "…suggesting the potential…" (L160);

(5) "…already possessed…" was changed to "…was inferred to have possessed…" (L262);

(6) "…capacity.." was changed to "…potential…" (L162; L185);

(7) "…showed…" was changed to "…indicated…" (L165);

(8) "…supporting…" was changed to "…consistent with…" (L196);

(9) "…and are consistent with our hypothesis of unique strategies for nutrient and energy acquisition" was changed to "…support potential specialized nutrient and energy acquisition strategies." (L239);

(10) "…the predatory lifestyle…" was changed to "…the putative predatory lifestyle…" (L242);

(11) "…possessed a relatively small proteome," was changed to "…is inferred to have had a relatively small proteome," (L247);

(12) "…was widely present…" was changed to "…is inferred to have been widely present…" (L280).

Comment 3: The study would benefit significantly from a comparison with oligotrophic aquatic environments. For example, deep oligotrophic lakes are unusually rich in CPR bacteria—relatives of Actinobacteria that also exhibit streamlined genomes and parasitic-like features.

Response 3: We sincerely thank the reviewer for highlighting the importance of comparing this study with oligotrophic aquatic systems and Candidate Phyla Radiation (CPR) bacteria. We agree that such comparisons provide valuable ecological context for interpreting our findings. **We would like to clarify that CPR bacteria are not closely related to Actinomycetota but instead represent a distinct deep branching lineage within the bacterial domain.** Phylogenomic analyses indicate that Actinomycetota fall outside the CPR radiation (Figure below). Nevertheless, Subterricolia and CPR lineages show clear functional convergence, including ultra-small cell size, genome reduction, loss of biosynthetic pathways, and dependence on other microorganisms. These similarities are likely driven by similar selective pressures in energy-limited environments.

In the revised Results section, we now note that toxins encoded in Subterricolia are also present in the known CPR predator Vampirococcus lugosii (L206): "These toxins are also present in Vampirococcus lugosii[31], a Candidate Phyla Radiation (CPR) bacterium with a predatory lifestyle, suggesting a potentially similar predatory strategy."

In the Discussion section, we have expanded the comparison between Subterricolia and oligotrophic aquatic microorganisms, including CPR lineages. We point out their shared features such as genome reduction, increased coding density, and ultra-small cell size, as well as the differences in GC content and proteomic C/N ratios between Subterricolia and these aquatic oligotrophs, and further discuss the reasons underlying these similarities and differences (L305–L315). "The reduced genomes, increased coding density, and ultra-small cell sizes lower non-essential metabolic costs and energy requirements during cell division and growth, resembling

those observed in nutrient-poor epipelagic marine and lacustrine microorganisms as well as Candidate Phyla Radiation (CPR) lineages[40-44], suggesting convergent adaptation to energy-limited environments. However, unlike these aquatic oligotrophs that tend to have low GC content and increased proteomic C/N ratios, Subterricolales exhibits high GC content and reduced proteomic C/N ratios alongside genome reduction. This difference reflects contrasting nutrient constraints, with aquatic systems generally being nitrogen-limited whereas soil microbial growth is predominantly carbon-limited[45]. Because GC base pairs have lower C/N ratios, selection for higher GC content and reduced proteomic C/N ratios can reduce cellular carbon demand[42,45]."

[Figure Redacted]

Response Figure 3. The phylogenetic tree was obtained from Hug et al. (2016), and the positions of Actinomycetota and Candidate Phyla Radiation (CPR) are highlighted.

Reference:

Hug L A, Baker B J, Anantharaman K, et al. A new view of the tree of life[J]. Nature microbiology, 2016, 1(5): 1-6.

Comment 4: The lack of microscopy to confirm the suggested parasitic lifestyle is a notable disadvantage. While size fractionation is used, it is a limited substitute for direct observation of host-parasite interactions.

Response 4: We have addressed this point. As noted in our response to Comment 1, we have better acknowledged this limitation in the Discussion section (L356–L359) and stated that future targeted cultivation combined with microscopy will be needed to validate the proposed predatory lifestyle

(L378–L379).

Comment 5: Ln 46: Please provide a formal definition of "Earth's Critical Zone."

Response 5: We have revised the Introduction to provide a formal definition of the Earth's Critical Zone (CZ) upon its first mention: L43: "The Earth's Critical Zone (CZ) is the near-surface layer of the Earth extending from the top of the vegetation canopy to subsurface groundwater, linking the atmosphere, pedosphere, biosphere, hydrosphere, and lithosphere, and supporting the functioning of terrestrial ecosystems."

Comment 6: Ln 79: The term "hypothesis" feels too strong here; "assumptions " may be more accurate.

Response 6: Thank you for this suggestion. We have changed (L82) "...we tested the following hypotheses:" into "... we investigated whether…"

Comment 7: Ln 85–95: This section would be more appropriate in the Introduction rather than the Results/Discussion.

Response 7: We thank the reviewer for this suggestion. We have carefully considered this point, but retained this section in the Results because **it presents results derived from our analyses rather than background information**. In this section (L88–L99), we examined the detection frequency, habitat preference, and global distribution pattern of Subterricolia using two global-scale 16S and metagenomic datasets, and found that it is predominantly distributed in soil habitats. This result then led us to further examine the relationship between Subterricolia relative abundance and environmental factors using soil 16S datasets with physicochemical measurements, thereby revealing its preference for nutrient-poor soils.

Comment 8: Ln 106: "1.6-4.7 times" may not be perceived as a substantial difference by all readers; please provide further context for this comparison.

Response 8: Thank you for pointing this out. We revised the original "1.6–4.7 times" description to a comparison between the average organic carbon contents of surface and deep soils, showing that surface soils contained approximately 3.6 times more organic carbon than deep soils. We also included the average organic carbon concentration in deep soils (0.27 ± 0.10%) to help readers better appreciate the resource-limited nature of the deep-soil environment.

We have changed the sentence into (L109) "In these profiles, the average organic content in surface soils was approximately 3.6-fold higher than in deep soils (Supplementary Fig. 4), while deep soils contained only 0.27 ± 0.10% organic carbon on average, indicating substantial resource limitation."

Comment 9: Ln 108: Briefly clarify how "relative abundance" was calculated. Specifically, it should be clear what the denominator represents (e.g., % of total reads, % of total community).

Response 9: We added "of total reads" after "relative abundance" to make the denominator explicit (L113). Here, relative abundance represents the proportion of reads assigned to Subterricolia relative to the total reads within each sample.

Comment 10: Ln 110: Please address the inherent bias of amplicon technology. Because of the amplification step, it cannot be considered strictly quantitative.

Response 10: We agree that amplicon sequencing is subject to amplification biases and cannot be considered strictly quantitative. For this reason, we have carefully used the term "relative abundance" throughout the manuscript. Moreover, the higher relative abundance of Subterricolia in deep soils was consistently observed across 1,032 samples from eight independent studies, which employed different primer sets targeting various 16S rRNA gene regions (V3, V3–V4, V4, V4–V5, and V1–V4). The consistency of this pattern across studies using different amplification conditions and primer combinations suggests that the observed enrichment in deep soils is robust.

Comment 11: Ln 134: High GC content is generally associated with large, "wasteful" genomes, whereas aquatic Actinobacteria (which are oligotrophic and streamlined) typically exhibit low GC content. The authors should comment on how their findings align with or diverge from this trend.

Response 11: We thank the reviewer for this insightful comment. We agree that the "small-genome/high-GC" pattern observed in Subterricolia differs from the classical genome-reduction model reported for many oligotrophic aquatic microorganisms, where genome reduction is typically associated with low GC content.

In marine and other aquatic environments, GC content and genome size are generally positively correlated, so reduced genomes tend to exhibit low GC content. In contrast, soil microbial communities often show a negative correlation between genome size and GC content (Chuckran et al., 2022). This contrast likely reflects different resource limitation regimes: oligotrophic marine systems are often nitrogen-limited, whereas microbial growth in soils is predominantly carbon-limited (Hobbie and Hobbie, 2013; Soong et al., 2020). Because GC base pairs have a lower C:N ratio (1.13) than AT base pairs (1.42), elevated GC content can reduce carbon demand per unit DNA. Therefore, under carbon-limited soil conditions, selection for smaller genomes is frequently accompanied by higher GC content.

We have incorporated this discussion into the revised manuscript (L309–L315): "However, unlike these aquatic oligotrophs that tend to have low GC content and increased proteomic C/N ratios, Subterricolales exhibits high GC content and reduced proteomic C/N ratios alongside genome reduction. This difference reflects contrasting nutrient constraints, with aquatic systems generally being nitrogen-limited whereas soil microbial growth is predominantly carbon-limited[45]. Because GC base pairs have lower C/N ratios, selection for higher GC content and reduced proteomic C/N ratios can reduce cellular carbon demand[42,45]."

References:

Chuckran P F, Hungate B A, Schwartz E, et al. Variation in genomic traits of microbial communities among ecosystems[J]. FEMS microbes, 2021, 2: xtab020.

Chuckran P F, Flagg C, Propster J, et al. Edaphic controls on genome size and GC content of bacteria in soil microbial communities[J]. Soil Biology and Biochemistry, 2023, 178: 108935.

Hobbie J E, Hobbie E A. Microbes in nature are limited by carbon and energy: the starving-survival lifestyle in soil and consequences for estimating microbial rates[J]. Frontiers in microbiology, 2013, 4: 324.

Soong J L, Fuchslueger L, Marañon‐Jimenez S, et al. Microbial carbon limitation: The need for integrating microorganisms into our understanding of ecosystem carbon cycling[J]. Global change biology, 2020, 26(4): 1953-1961.

Giovannoni S J, Cameron Thrash J, Temperton B. Implications of streamlining theory for microbial ecology[J]. The ISME journal, 2014, 8(8): 1553-1565.

Comment 12: Ln 142: It is worth noting that this phenomenon mirrors observations in deep ocean waters. (reduce carbon but increase nitrogen usage (Fig. 3b).

Response 12: We have expanded the Discussion (L309-L315) to compare Subterricolales with oligotrophic aquatic microorganisms, including epipelagic marine lineages, and highlighted that different nutrient constraints drive differences in GC content and proteomic C/N ratios. Accordingly, deep-ocean microorganisms exhibit a similar "more N, less C" protein composition pattern to Subterricolales, suggesting that they experience comparable nutrient constraints. Previous studies have reported that, unlike nitrogen-limited surface oceans, deep oceans are primarily carbon-limited.

Comment 13: Ln 187 onwards: Is there a documented relationship between Subterricolia and CPR bacteria? They share similar gene profiles; a brief discussion on potential relatedness is warranted.

Response 13: We thank the reviewer for this question. As noted in our Response 3, to our knowledge, there is currently no evidence for a close evolutionary relationship between Subterricolia and CPR lineages. In the revised Discussion, we interpret the shared features between them as convergent adaptations to energy-limited environments, whereas the difference in GC content reflects contrasting nutrient constraints between aquatic and soil environments. The specific revisions are detailed in Response 3.

Comment 14: Ln 207 onwards: The analogy to CPR bacteria is quite clear here, yet it seems to be introduced for the first time late in the manuscript. A comparative genomics analysis between these two groups, including the identification of the closest relative to Subterricolales within the CPR, would significantly strengthen the paper.

Response 14: This point has been addressed in our responses to Comments 3 and 13. Subterricolia and CPR lineages are not closely related evolutionarily, and there is currently no evidence for a close phylogenetic relationship between them. In the revised Discussion (L308–L315), we have expanded the comparison between Subterricolia and CPR lineages, highlighting their shared features, including genome reduction, increased coding density, and ultra-small cell size, while also discussing their differences in GC content and the potential ecological drivers underlying these patterns.

Responses to Reviewers' Comments

Reviewer #1

I am generally happy with the revisions made and the additional information provided regarding sampling, controls etc, as well as the improved discussion. I have only very few final comments.

Comment 1: Note S3 is not referenced in the text, please add at appropriate place.

Response 1: We sincerely thank the reviewer for the positive feedback and valuable suggestions. We have now cited Supplementary Note S1 (formerly Supplementary Note S3) at the appropriate positions in the main text, including when mentioning global distribution (L88), environmental drivers (L100), vertical distribution (L114), and the integrated Aridivitia MAG collection (L126).

Comment 2: L88: Still this global angle comes out of the blue, as it is also not so well introduced in the methods. Note S3 gives a nice summary of it, but it needs to be referenced in the main text. It might for example fit at the end of the introduction. Currently there you only explain your experimental approach, but it would help to outline the different steps starting from bioinformatic over the specific new samplings etc. Alternatively, you might implement a short version of Note 3 at the beginning of the methods section.

Response 2: We have followed the reviewer's suggestion and made the following revisions.

(1) We revised the Methods section "Environmental distribution and ecological drivers" to better introduce the rationale for analyzing the global distribution of Aridivitia. The revised section now explicitly states that EMP and Sandpiper were used to assess the global distribution of Aridivitia (L436), the European and Australian soil survey datasets were used to examine its environmental associations (L446), and independent soil profile datasets were used to test whether its deep-soil enrichment represents a widespread pattern (L453). Thus, this section now serves as a brief version of Supplementary Note S1 (former Supplementary Note S3).

(2) We cited Supplementary Note S1 at the beginning of the Results section (L85) "Global soil distribution of Aridivitia" so that readers can obtain more detailed dataset information when first encountering the global perspective.

Comment 3: L411: Also add the purpose of using both greengenes versions in the main text, maybe in brackets.

Response 3: In response to the reviewer's suggestion, we have clarified the rationale for employing both Greengenes 13.8 and Greengenes 2 in the parenthetical statement at L408 "……. both the Greengenes 13.8 and Greengenes2 databases (to maintain consistency with the historical 0319-7L14 designation while also leveraging the updated taxonomy)"

Notably, during the review of our manuscript, SeqCode names became available for the focal class-level lineage and three of its constituent orders. Following discussions with microbial taxonomy expert James R. Cole (colejr@gmail.com) and Luis M. Rodriguez-R from the SeqCode

Registry team (mr@bio.aau.dk), we followed their recommendation and adopted the SeqCode-validated name Aridivitia instead of Subterricolia. We also added the following clarification in the Methods section (L491–L500) to explain the nomenclature and facilitate its interpretation by future readers:

L491-L500 "In the current SeqCode Registry, the class Aridivitia1 contains three named orders: Aridivitales1 (later subjective synonym Actinopolaridales2, Bungeriellales2, and Actinosomnolentales2. These three lineages correspond to o__CADDZG01, o__JAHWKV01, and o__UBA4738, respectively, within c__UBA4738 in GTDB R220. Later GTDB releases revised the taxonomic circumscription of these clades; for example, in GTDB R232, the lineage corresponding to Actinosomnolentales was promoted to the class level, whereas Aridivitales and Bungeriellales were retained within c__CADDZG01 together with o__AC-67. In this paper, we refer to Aridivitia and this sister class collectively as the Aridivitia group, or simply Aridivitia."
